# Supplementary material for: Elp3‐mediated codon‐dependent translation promotes mTORC2 activation and regulates macrophage polarization
Source: EMBO J. 2022 Aug 3;41(18):e109353. doi: 10.15252/embj.2021109353 (PMC9475509; doi:10.15252/embj.2021109353)
Supplement: Supplementary file 1 — Expanded View Figures PDF [file EMBJ-41-e109353-s004.pdf]

Expanded View Figures

Figure EV1. Expression of Elongator subunits and Ctu1/2 by M1 polarization signals.

A, B M1 polarization signals downregulate the expression of tRNA-modifying enzymes at both mRNA (A) and protein (B) levels. Peritoneal macrophages were untreated or stimulated with IFNγ (50 ng/ml), LPS (100 ng/ml) or with both IFNγ and LPS *ex-vivo* for the indicated periods of time and mRNA levels of the indicated candidates were assessed in all experimental conditions by Real-Time PCR (A). mRNA levels in unstimulated cells were set to 1 and levels in other experimental conditions were relative to that after normalization with Gapdh mRNA levels ($n = 3$ mice; mean ± SD, Student t-test, *$P < 0.05$; **$P < 0.01$; ***$P < 0.001$). Protein levels (B) were assessed by western blot analyses using the indicated antibodies.

C BMDMs and intestinal epithelial cells (IECs) lacking *Elp3* show decreased thiolated tRNA levels. Northern blots were carried out with enriched small RNAs extracted from BMBMs of $Elp3^{Control}$ and $Elp3^{\Delta Mye}$ mice or with total RNAs from IECs of $Elp3^{Control}$ and $Elp3^{\Delta IEC}$ mice, using the indicated probes. Values are from three independent measurements (mean ± SD, ****$P < 0.0001$, Welch one-way Anova test).

D Elp3 expression in myeloid cells is dispensable in the architecture of intestinal crypts. Colons from both $Elp3^{Control}$ and $Elp3^{\Delta Mye}$ mice were subjected to IHC analyses.

E Colon length is not altered upon *Elp3* deficiency in myeloid cells. Data at day 6 from 8 mice per group are illustrated (mean ± SD, Student t-test, nonsignificant).

F mRNA levels of pro-inflammatory cytokines in peritoneal macrophages lacking Elp3 do not change in unstimulated mice. mRNAs of the listed pro-inflammatory cytokines in peritoneal macrophage from $Elp3^{Control}$ or $Elp3^{\Delta Mye}$ mice were assessed by Real Time PCR. mRNA levels in $Elp3^{Control}$ mice were set to 1 and levels in other experimental conditions were relative to that after normalization with Gapdh mRNA levels ($n = 5$ mice; mean ± SD, nonsignificant).

Source data are available online for this figure.

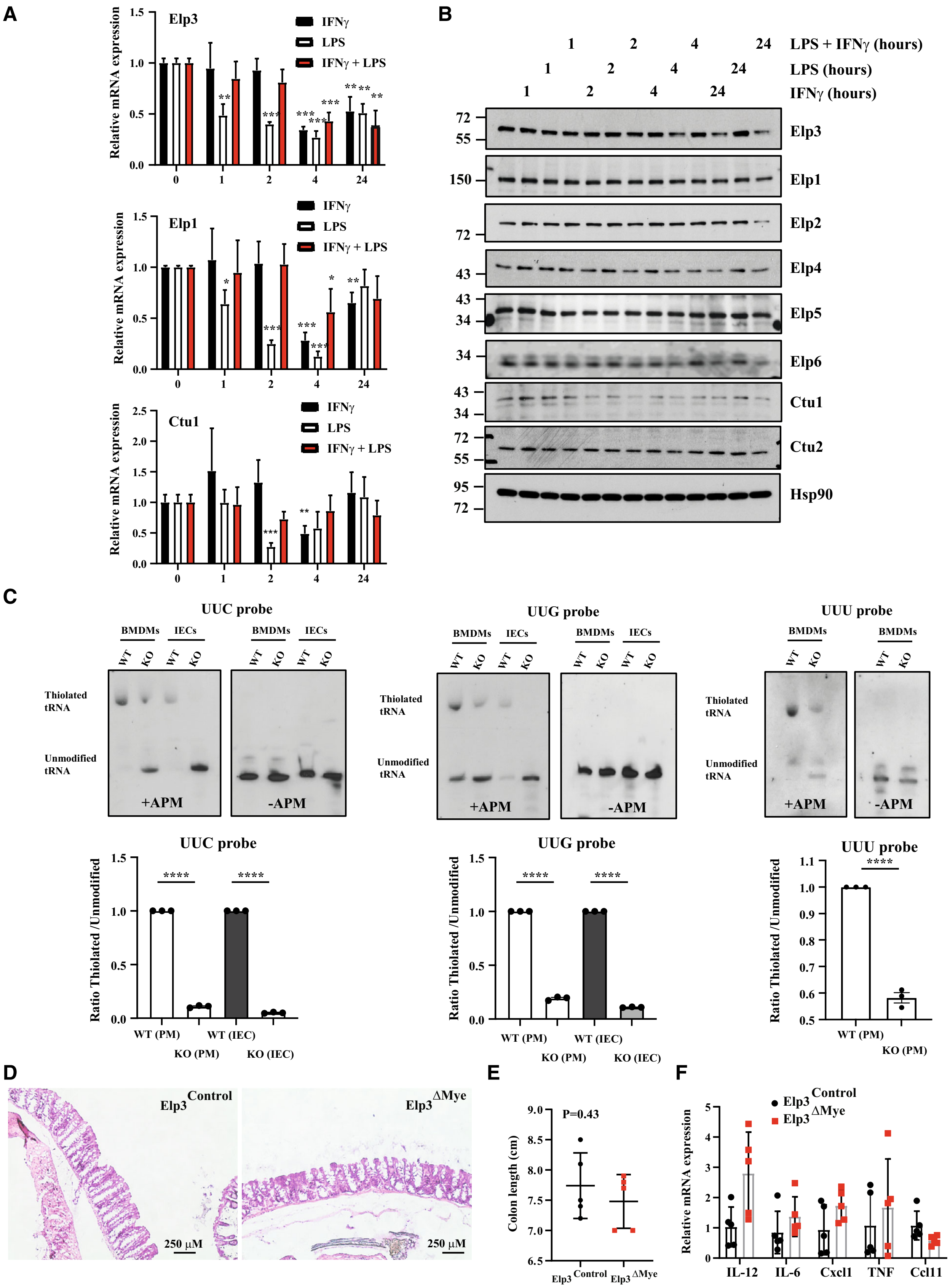

Figure EV1.

 © 2022 The Authors

Figure EV2. Ctu2 promotes IL-4-dependent mTORC2 activation and M2 macrophage polarization.

A Elp1/3 and Ctu1/2 expression are induced by M2 polarization signals. Peritoneal macrophages were untreated or stimulated with IL-4 (20 ng/ml), IL-13 (20 ng/ml) or with both IL-4 and IL-13 *ex-vivo* for the indicated periods of time and mRNA levels of the indicated candidates were assessed in all experimental conditions by Real-Time PCR. mRNA levels in unstimulated cells were set to 1 and levels in other experimental conditions were relative to that after normalization with Gapdh mRNA levels ($n = 3$ mice; mean ± SD, Student t-test, *$P < 0.05$; **$P < 0.01$; ***$P < 0.001$).

B IL-4-dependent mTORC2 activation relies on Ctu2. Control and Ctu2-depleted bone marrow-derived macrophages (BMDMs) were treated or not with IL-4 (10 ng/ml) and the resulting cell extracts were subjected to western blot (WB) analyses.

C Ctu2 deficiency impairs M2 macrophage polarization. Control and Ctu2-depleted BMDMs were treated or not with IL-4 (10 ng/ml) for 24 h and the resulting mRNAs of the listed M2 markers were assessed by Real Time PCR. mRNA levels in control BMDMs were set to 1 and levels in other experimental conditions were relative to that after normalization with Gapdh mRNA levels ($n = 3$ mice; mean ± SD, Student t-test, *$P < 0.05$, **$P < 0.01$, ***$P < 0.001$).

Source data are available online for this figure.

© 2022 The Authors

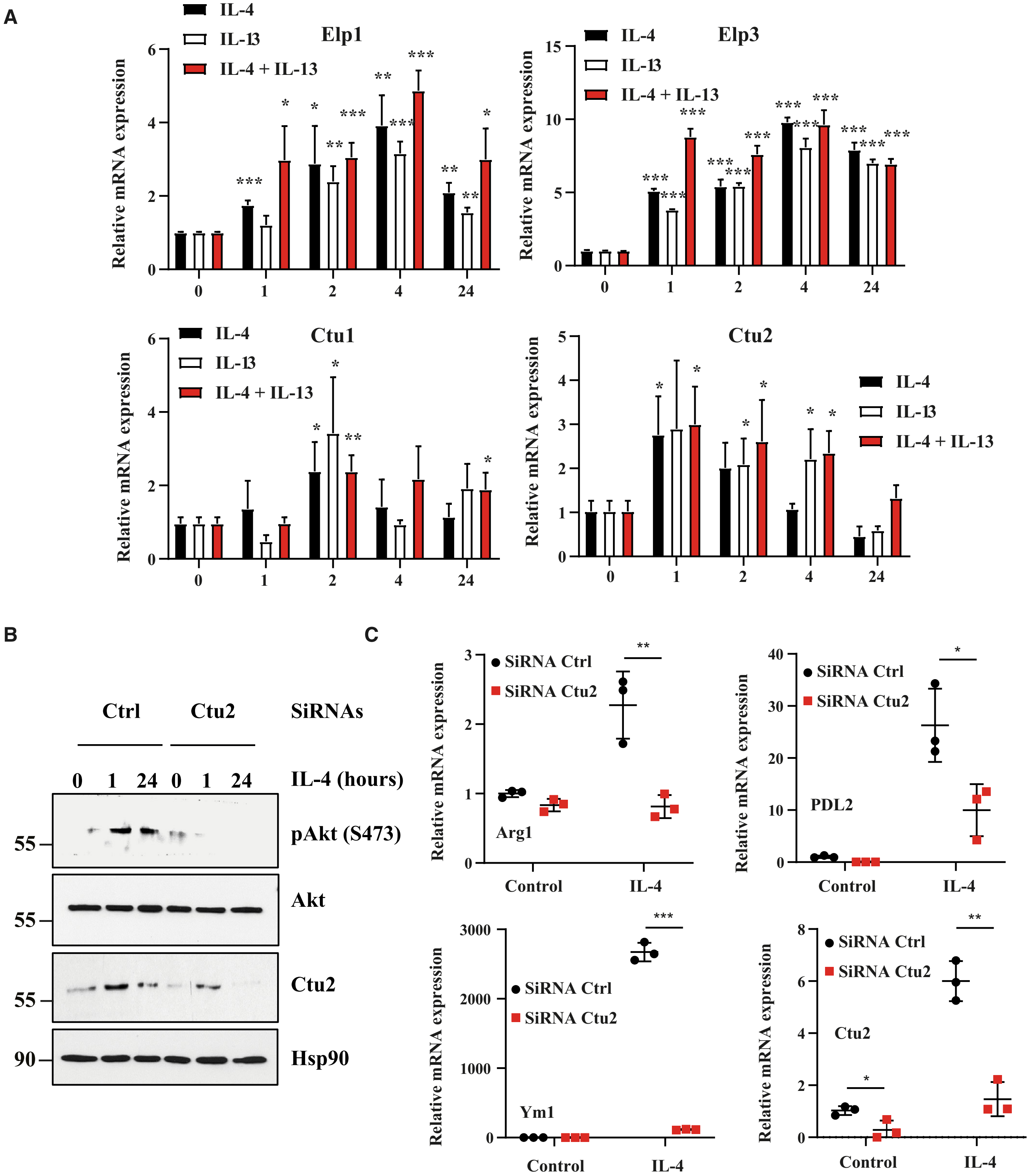

Figure EV2.

 © 2022 The Authors

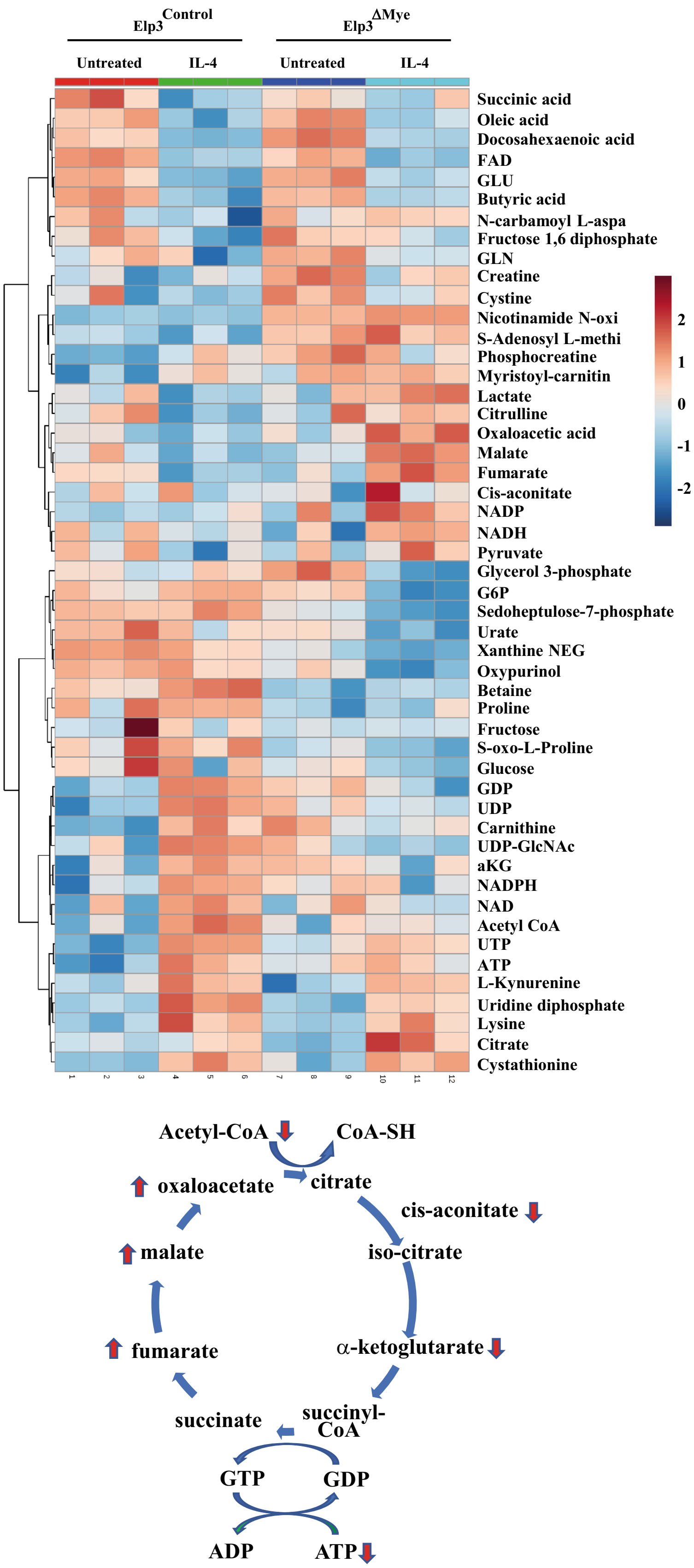

Figure EV3. Metabolic reprogramming by IL-4 relies on Elp3.

Peritoneal macrophages from *Elp3*^{Control} and *Elp3*$^{\Delta Mye}$ mice (n = 3 per genotype) were treated with IL-4 for 24 h *ex-vivo* and the resulting extracts were subjected to Mass Spec analyses to extensively quantify metabolites. At the bottom, defective TCA cycle upon *Elp3* deficiency in IL-4-treated macrophages. Red arrows identify TCA metabolites whose levels change upon *Elp3* deficiency.

© 2022 The Authors

Figure EV4. Elp3 promotes Ric8b expression.

A Elp3 does not regulate Ric8b mRNA levels. mRNAs of BMDMs from the indicated genotypes and stimulated *ex-vivo* with IL-4 or with PBS (control) were subjected to Real-Time PCRs. mRNA levels of Ric8b in BMDMs treated with PBS from $Elp3^{Control}$ mice were set to 1 and levels in other experimental conditions were relative to that after normalization with Gapdh mRNA levels ($n = 3$ mice per genotype (mean ± SD).

B Ric8b promotes LPS-dependent mTORC2 activation. Control or Ric8b-depleted BMDMs were treated or not with IFNγ (50 ng/ml) and LPS (100 ng/ml) for the indicated periods of time and the resulting cell extracts were subjected to WB analyses.

C Increased aggregate formation by IL-4 in macrophages. Flow cytometry of aggresomes with extracts of BMDMs from $Elp3^{Control}$ and $Elp3^{\Delta Mye}$ mice treated with IL-4 (10 ng/ml) for 24 h are illustrated. Experiments were conducted in duplicates.

D Visualization of protein aggregates. Coomassie blue staining of aggregate samples from $Elp3^{Control}$ and $Elp3^{\Delta Mye}$ BMDMs treated with IL-4 or PBS (control; $n = 8$ mice).

E Identification of a proteomic signature of candidates whose expression is increased upon IL-4 stimulation and in BMDMs lacking Elp3 (Venn diagram).

F Identification of proteins enriched in aggregates in macrophages lacking Elp3. Dot plot of protein aggregates from IL-4-treated BMDMs cells lacking or not *Elp3* are shown. Genes enriched in Lys^{AAA}, Gln^{CAA}, and Glu^{GAA} codons are shown in blue. Mrpls are shown in Red.

G The expression of selected Mrpl proteins is controlled by Elp3 in macrophages. Peritoneal macrophages from $Elp3^{Control}$ and $Elp3^{\Delta Mye}$ mice were treated or not with IL-4/IL-13 (10 ng/ml) for the indicated periods of time and cell extracts from resulting cells were subjected to WBs using the indicated antibodies.

H Elp3 does not regulate mRNA levels of Mrpl proteins. PMs from the indicated genotypes were stimulated or not with IL-4/IL-13 (10 ng/ml) for 24 h and mRNA levels of the indicated candidates were quantified by Real-Time PCR. mRNA levels of all candidates in untreated cells from $Elp3^{Control}$ mice were set to 1 and levels in other experimental conditions were relative to that after normalization with Gapdh mRNA levels ($n = 3$ mice, mean ± SD, Student t-test, nonsignificant).

I Elp3 controls Mt-co3 expression in macrophages. Protein extracts from PMs of the indicated genotypes treated or not with IL-4/IL-13 (10 ng/ml) for the indicated periods of time were subjected to WB analyses.

Source data are available online for this figure.

© 2022 The Authors

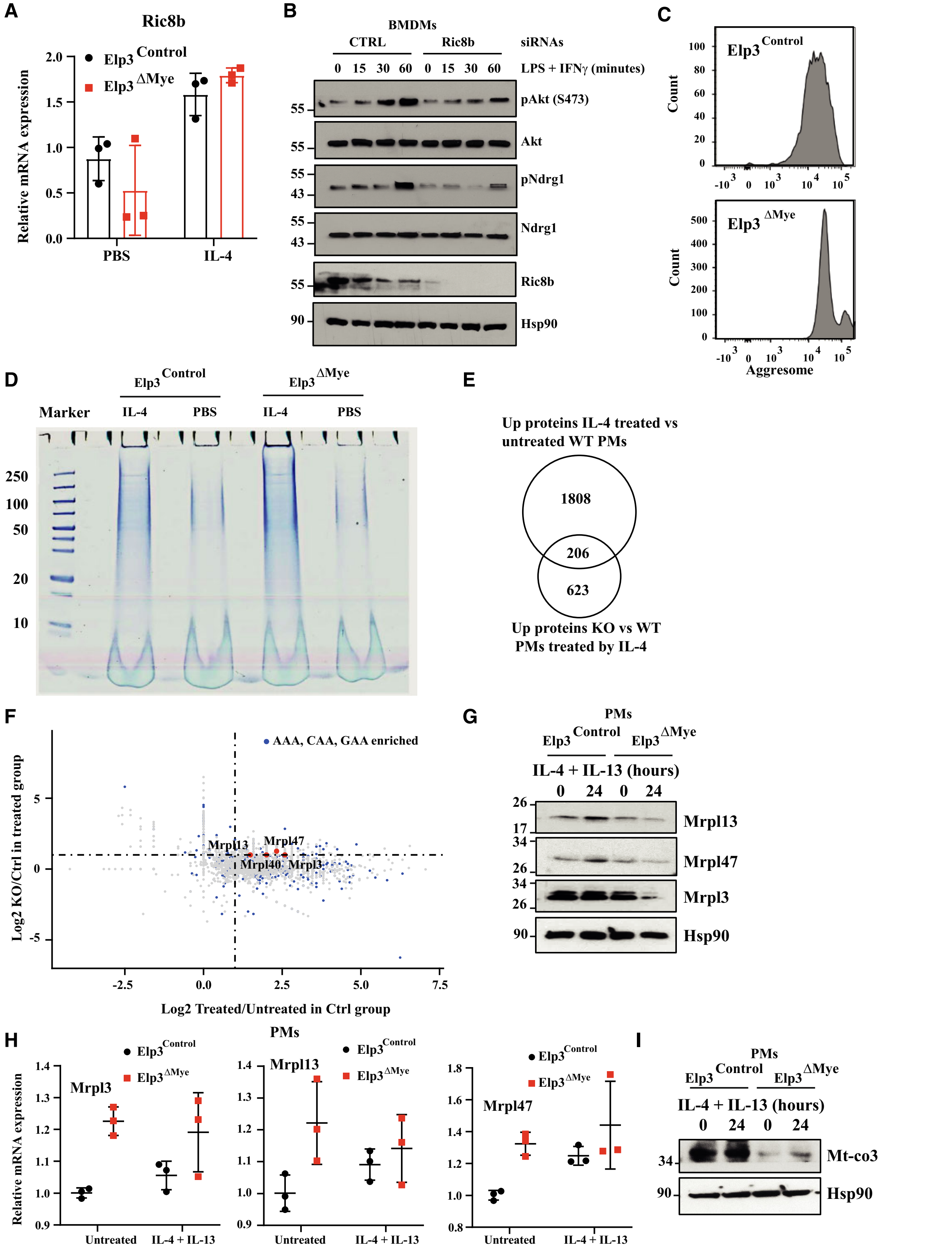
A
Ric8b
Elp3 Control
Elp3 ΔMye
Relative mRNA expression
PBS
IL-4
B
BMDMs
CTRL
Ric8b
siRNAs
0 15 30 60 0 15 30 60
LPS + IFNγ (minutes)
pAkt (S473)
Akt
pNdrg1
Ndrg1
Ric8b
Hsp90
C
Elp3 Control
Count
Elp3 ΔMye
Aggresome
D
Elp3 Control
Elp3 ΔMye
Marker
IL-4
PBS
E
Up proteins IL-4 treated vs untreated WT PMs
1808
206
623
Up proteins KO vs WT PMs treated by IL-4
F
AAA, CAA, GAA enriched
Mrpl13
Mrpl47
Mrpl40
Mrpl3
Log2 KO/Ctrl in treated group
Log2 Treated/Untreated in Ctrl group
G
PMs
IL-4 + IL-13 (hours)
Mrpl13
Mrpl47
Mrpl3
Hsp90
H
PMs
Mrpl3
Mrpl13
Mrpl47
Relative mRNA expression
Untreated
IL-4 + IL-13
I
PMs
Mt-co3
Hsp90

Figure EV4.

Figure EV5. Elp3 deficiency in peritoneal macrophages does not stabilize Atf4.

A Peritoneal macrophages from the indicated genotypes were stimulated or not with IL-4/IL-13 (10 ng/ml) for the indicated periods of time and the resulting extracts were subjected to WB analyses.

B *Elp3* deficiency does not potentiates Atf4-driven transcription in myeloid cells. Peritoneal macrophages from the indicated genotypes were stimulated or not with IL-4 (10 ng/ml) for 24 h and mRNA levels of the indicated Atf4 target genes were quantified by Real-Time PCR. mRNA levels of all candidates in untreated cells from *Elp3*^{Control} mice were set to 1 and levels in other experimental conditions were relative to that after normalization with Gapdh mRNA levels (n = 4 mice, mean ± SD, Student t-test, nonsignificant).

Source data are available online for this figure.

 © 2022 The Authors

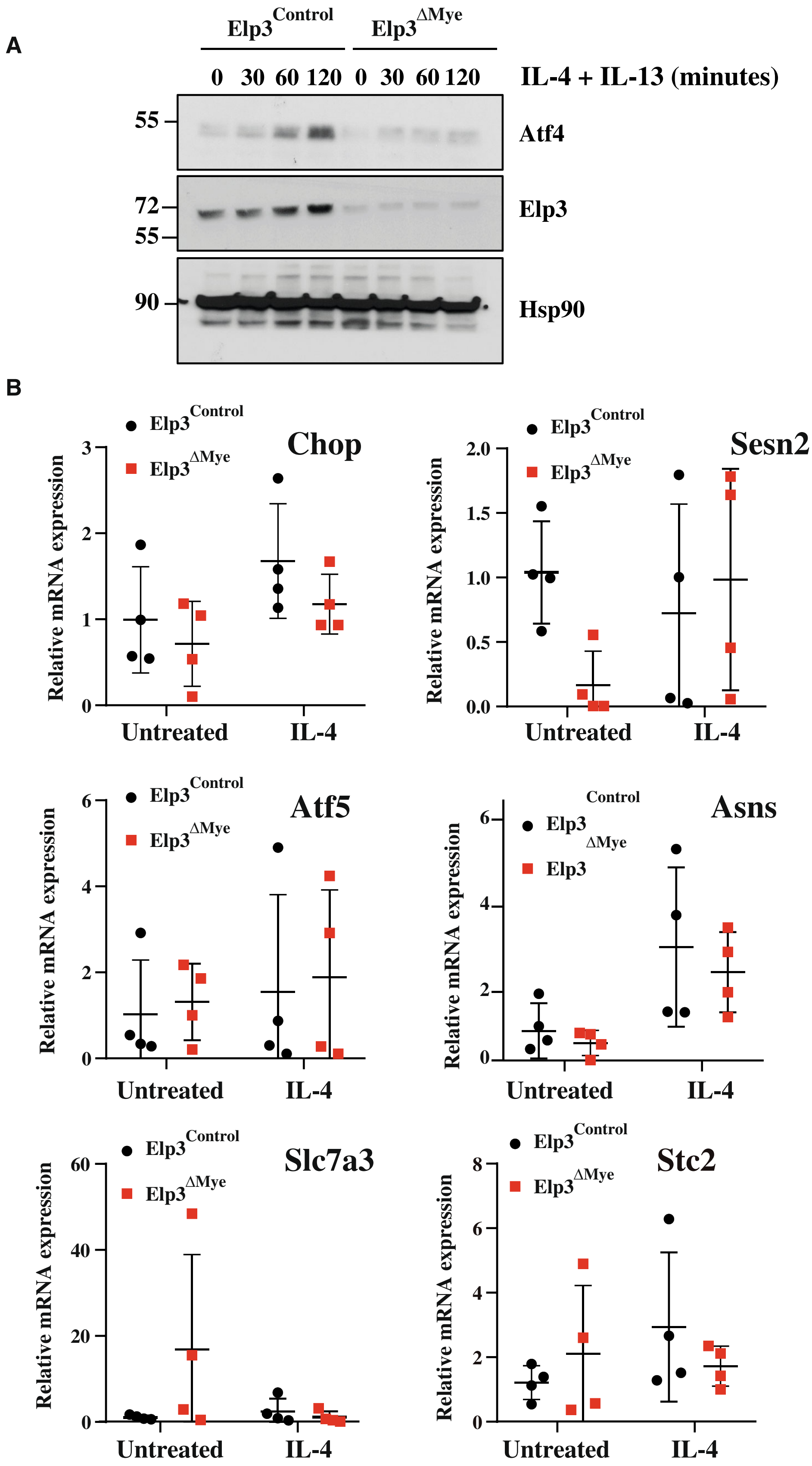
A
Elp3^Control
Elp3^ΔMye
0 30 60 120 0 30 60 120
IL-4 + IL-13 (minutes)
55
Atf4
72
55
Elp3
90
Hsp90
B
Elp3^Control
Elp3^ΔMye
Chop
Sesn2
Atf5
Asns
Slc7a3
Stc2
Relative mRNA expression
Untreated
IL-4

Figure EV5.
