# Supplementary material for: Elp3‐mediated codon‐dependent translation promotes mTORC2 activation and regulates macrophage polarization
Source: EMBO J. 2022 Aug 3;41(18):e109353. doi: 10.15252/embj.2021109353 (PMC9475509; doi:10.15252/embj.2021109353)
Supplement: Supplementary file 2 — Table EV1 [file EMBJ-41-e109353-s008.docx]

| Antibody | Company | Catalogue number | Dilution |
| --- | --- | --- | --- |
| Elp3 | Cell Signaling Technology | 5728 | 1/1000 |
| Ctu1 | Abcam | ab185473 | 1/1000 |
| Ctu2 | Abcam | ab177160 | 1/1000 |
| Hsp90 | Santa Cruz Biotechnology | Sc-13119 | 1/10000 |
| Gapdh | Cell Signaling Technology | 5174 | 1/1000 |
| pFoxo1 (S256) | Cell Signaling Technology | 9461 | 1/1000 |
| Foxo1 | Cell Signaling Technology | 2880 | 1/1000 |
| pAkt (S473) | Cell Signaling Technology | 4060 | 1/1000 |
| Akt | Cell Signaling Technology | 2920 | 1/1000 |
| Rictor | Cell Signaling Technology | 9476 | 1/1000 |
| Raptor | Cell Signaling Technology | 2280 | 1/1000 |
| α-Tubulin | Sigma | T6199 | 1/10000 (WB) |
| pStat6 | Cell Signaling Technology | 9361 | 1/1000 |
| Stat6 | Cell Signaling Technology | 5397 | 1/1000 |
| Socs1 | Cell Signaling Technology | 3950 | 1/1000 |
| pS6K | Cell Signaling Technology | 9204 | 1/1000 |
| S6K | Cell Signaling Technology | 9202 | 1/1000 |
| Arg1 | GeneTex | GTX109242 | 1/1000 |
| CD71 | Abcam | Ab84036 | 1/1000 |
| Pparγ | Santa Cruz Biotechnology | Sc7273 | 1/1000 |
| Vdac1 | Abcam | Ab14734 | 1/1000 |
| F4/80 | Cell Signaling Technology | 70076 | 1/100 (IF) |
| CD301 | Bio-Rad | MCA2392 | 1/100 |
| Ric8b | Atlas | HPA042746 | 1/1000 |
| Ndrg1 | Cell signaling | 5196 | 1/1000 |
| p-Ndrg1 | Cell signaling | 5482 | 1/1000 |
| Elp1 | Santa Cruz Biotechnology | Sc8336 | 1/1000 |
| Atf4 | Cell Signaling | 11815 | 1/1000 |
| Il13ra1 | Abcam | ab79277 | 1/1000 |
| pTyk2 | Cell Signaling | 9321 | 1/1000 |
| Tyk2 | Cell Signaling | 35615 | 1/1000 |
| Mrpl3 | Atlas | HPA043665 | 1/1000 |
| Mrpl13 | Thermo Fisher | PA5-51007 | 1/1000 |
| Mrpl47 | Thermo Fisher | PA5-101365 | 1/1000 |
| Mt-co3 | Thermo Fisher | 55082-1-AP | 1/1000 |
| Sdha | Cell Signaling | 11998 | 1/1000 |
| Puromycin | DSHB | PMY-2A4 | 1/60 |
| Tubulin | Abcam | ab6160 | 1/1000 (IF) |
| Elp2 | Abclonal | A15857 | 1/1000 |
| Elp4 | Thermo Fisher | PA5-29307 | 1/1000 |
| Elp5 | Abclonal | A14862 | 1/1000 |
| Elp6 | Novus | NBP1-91733 | 1/1000 |
| CD11b | Biolegend | 101206 | 1/1000 |
| F4/80 | eBiosciences | 48-4801-82 | 1/500 (FACS) |
| CD115 | Biolegend | 165003 | 1/500 |
| MHCII | Biolegend | 107629 | 1/500 |
| Ki67 | eBiosciences | 46-5698 | 1/200 |
| ICAM2 | BD Biosciences | 564677 | 1/500 |

**EV Table 1: List of antibodies used in this study.**
