# Supplementary material for: Elp3‐mediated codon‐dependent translation promotes mTORC2 activation and regulates macrophage polarization
Source: EMBO J. 2022 Aug 3;41(18):e109353. doi: 10.15252/embj.2021109353 (PMC9475509; doi:10.15252/embj.2021109353)
Supplement: Supplementary file 3 — Table EV2 [file EMBJ-41-e109353-s013.docx]

| **Oligonucleotides** | | |
| --- | --- | --- |
| Arg1 for | IDT DNA | CTCCAAGCCAAAGTCCTTAGAG |
| Arg1 rev | IDT DNA | AGGAGCTGTCATTAGGGACATC |
| Elp3 for | IDT DNA | CAGTCCCTCCTCACTATCGAA |
| Elp3 rev | IDT DNA | TCTGTGGGGTTTGCACATCAC |
| Elp1 for | IDT DNA | TCGGCGGTTCCTTTCCAAAC |
| Elp1 rev | IDT DNA | GGTCCGATGCAACTTCAGATT |
| Il6 for | IDT DNA | TAGTCCTTCCTACCCCAATTTCC |
| Il6 rev | IDT DNA | TTGGTCCTTAGCCACTCCTTC |
| Il1b for | IDT DNA | GCAACTGTTCCTGAACTCAACT |
| Il1b rev | IDT DNA | ATCTTTTGGGGTCCGTCAACT |
| Il12b for | IDT DNA | TGGTTTGCCATCGTTTTGCTG |
| Il12b rev | IDT DNA | ACAGGTGAGGTTCACTGTTTCT |
| Cxc11 for | IDT DNA | CTGGGATTCACCTCAAGAACATC |
| Cxc11 rev | IDT DNA | CAGGGTCAAGGCAAGCCTC |
| Ccl11 for | IDT DNA | GAATCACCAACAACAGATGCAC |
| Ccl11 rev | IDT DNA | ATCCTGGACCCACTTCTTCTT |
| Tnf for | IDT DNA | CCCTCACACTCAGATCATCTTCT |
| Tnf rev | IDT DNA | GCTACGACGTGGGCTACAG |
| Chil3 for | IDT DNA | CAGGTCTGGCAATTCTTCTGAA |
| Chil3 rev | IDT DNA | GTCTTGCTCATGTGTGTAAGTGA |
| Pdcd11g2 for | IDT DNA | CTGCCGATACTGAACCTGAGC |
| Pdcd11g2 rev | IDT DNA | GCGGTCAAAATCGCACTCC |
| Ucp1 for | IDT DNA | AGGCTTCCAGTACCATTAGGT |
| Ucp1 rev | IDT DNA | CTGAGTGAGGCAAAGCTGATTT |
| Irf4 for | IDT DNA | TCCGACAGTGGTTGATCGAC |
| Irf4 rev | IDT DNA | CCTCACGATTGTAGTCCTGCTT |
| Rictor for | IDT DNA | GCTGCGCTATCTCATCCAAGA |
| Rictor rev | IDT DNA | GGGTTCTGAAGTGCTAGTTCAC |
| Slc2a1 for | IDT DNA | CAGTTCGGCTATAACACTGGTG |
| Slc2a1 rev | IDT DNA | GCCCCCGACAGAGAAGATG |
| Pfkp for | IDT DNA | GAAACATGAGGCGTTCTGTGT |
| Pfkp rev | IDT DNA | CCCGGCACATTGTTGGAGA |
| Gpi1 for | IDT DNA | TCAAGCTGCGCGAACTTTTTG |
| Gpi1 rev | IDT DNA | GGTTCTTGGAGTAGTCCACCAG |
| Ric8b for | IDT DNA | GACAAGCATAGGGCTACTTTCAA |
| Ric8b rev | IDT DNA | GGCATGTCGTTGGGACCTC |
| Ctu2 for | IDT DNA | GGATTCTGCGAAACGGCTAC |
| Ctu2 rev | IDT DNA | CACCTCTTCTAAGGCAACCAC |
| Sens2 for | IDT DNA | CGCCACTCAGAGAAGGTTCA |
| Sens2 rev | IDT DNA | ACGGGGTAGTCAGGTCATGT |
| Chop for | IDT DNA | GGAGAGAGTGTTCCAGAAGGAAG |
| Chop rev | IDT DNA | CGTCTCCAAGGTGAAAGGCA |
| Asns for | IDT DNA | GACTCTAAGGTGGGAAGCGG |
| Asns rev | IDT DNA | CAGGCACTCTGAGCACTAGC |
| Atf5 for | IDT DNA | AGATGAGGTCCTCCACCTTCG |
| Atf5 rev | IDT DNA | AATGGAGGCTGCACCAACAA |
| Slc7a3 for | IDT DNA | CATTGAAGCACACCCAACCC |
| Slc7a3 rev | IDT DNA | TAGCTAGGTTCGGAGAGCCC |
| Stc2 for | IDT DNA | GCTGTGGTGTGTTTGAGTGTT |
| Stc2 rev | IDT DNA | ATGAATGACTTTCCCTGGGCAT |
| Mrpl3 for | IDT DNA | TGACCCCGGCTTAGAGAGAAG |
| Mrpl3 rev | IDT DNA | ACCAGTTGCTTAACAAAGGAGAG |
| Mrpl13 for | IDT DNA | GCAAACTTGCGGTTATAGCATC |
| Mrpl13 rev | IDT DNA | GAGGAGTACACTTTTTGTTCCCA |
| Mrpl47 for | IDT DNA | AGAAGAGCCTCAGCGTTCCT |
| Mrpl47 rev | IDT DNA | GAGCAGCGTCCTTGGGATTTA |
| Pparg for | IDT DNA | TCGCTGATGCACTGCCTATG |
| Pparg rev | IDT DNA | GAGAGGTCCACAGAGCTGATT |
| Vdac1 for | IDT DNA | CCCACATACGCCGATCTTGG |
| Vdac1 rev | IDT DNA | GTGGTTTCCGTGTTGGCAGA |
| CHIP for | IDT DNA | ATTATTCGTCAGGGCTGCAC |
| CHIP rev | IDT DNA | AGGTGGTCAAAGTCTTTATCGCT |

**EV Table 2: List of primers used in this study.**
