# Supplementary material for: Elp3‐mediated codon‐dependent translation promotes mTORC2 activation and regulates macrophage polarization
Source: EMBO J. 2022 Aug 3;41(18):e109353. doi: 10.15252/embj.2021109353 (PMC9475509; doi:10.15252/embj.2021109353)
Supplement: Supplementary file 4 — Table EV3 [file EMBJ-41-e109353-s001.docx]

| Target | SiRNA Sequences |
| --- | --- |
| Elp3 | CAUCCGAAGUUUACACGAU |
| Ctu2 | \| Sequence 1 \| GAGUCAGGAUUCUGCGAAA \| \| --- \| --- \| \| Sequence 2 \| UGUUAUAGCUGCCGAGUGA \| \| Sequence 3 \| AUCCAGGAGUAUCUGAUUA \| \| Sequence 4 \| CCGUGUACAGGACGAGUGA \| |
| Ric8b | \| Sequence 1 \| ACAUAAAGGUCAUUCGUAU \| \| --- \| --- \| \| Sequence 2 \| ACUAAAACCUGACGGGACA \| \| Sequence 3 \| AGUGUGUCAUUUAGCGAAA \| \| Sequence 4 \| GUGCAUAAAGAGAGCGAUU \| |
| Rictor | \| Sequence 1 \| CUUAGAAGAUCUCGUGAAA \| \| --- \| --- \| \| Sequence 2 \| AUGUAGAAUUAGAGCGAAU \| \| Sequence 3 \| CAUUAAUUGCGGUUGGAAA \| \| Sequence 4 \| CGAUAUUGGCCAUAGUGAA \| |
| Raptor | \| Sequence 1 \| UAGAGGUAGCUGCGAUUAA \| \| --- \| --- \| \| Sequence 2 \| AUACUGACCGGGAGACGAA \| \| Sequence 3 \| AGAAUGAAGGAUCGGAUGA \| \| Sequence 4 \| CUGAGGAACACUCGAGUCA \| |
| Mrpl13 | \| Sequence 1 AGACGAGGAUAUUCCGGAA \| \| --- \| \| Sequence 2 CUGCGCAGCUUCACCGGAA \| \| Sequence 3 CCGUGGCGAUUGUGAAUU \| \| Sequence 4 AGACCACGUUGUCAUAAUA \| |
| Control | UAGCGACUAAACACAUCAA |

**EV Table 3: List of SiRNAs and their sequences used in this study.**
