# Supplementary figures and images for: Elp3‐mediated codon‐dependent translation promotes mTORC2 activation and regulates macrophage polarization
Source: EMBO J. 2022 Aug 3;41(18):e109353. doi: 10.15252/embj.2021109353 (PMC9475509; doi:10.15252/embj.2021109353)

**EV. 1B**

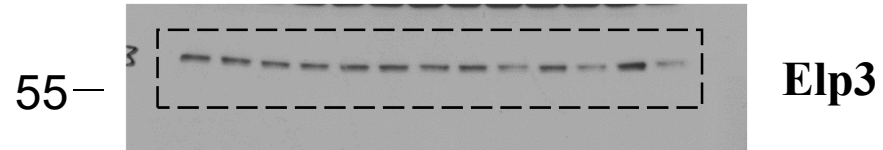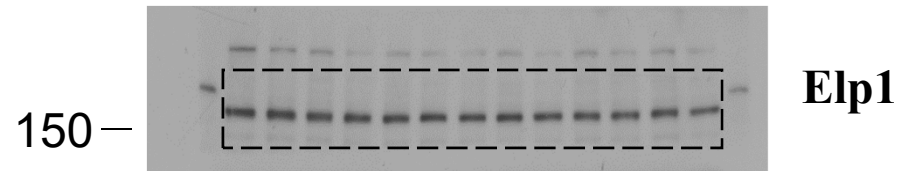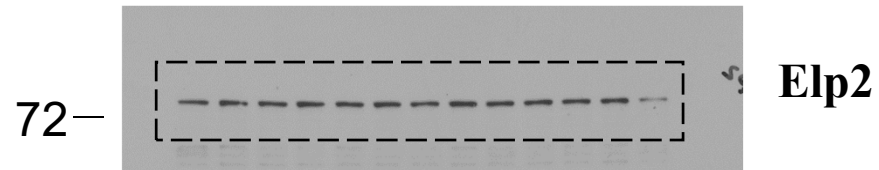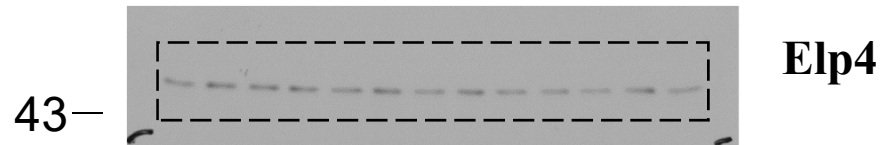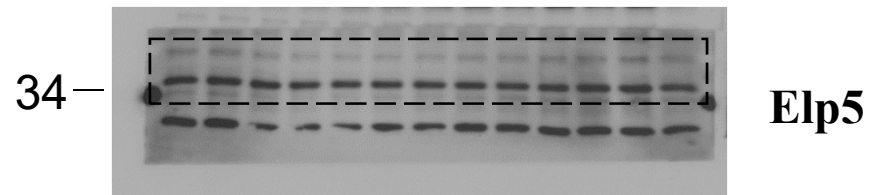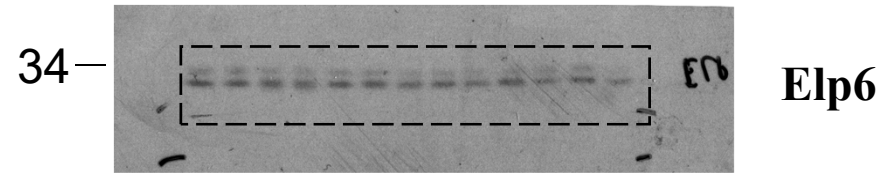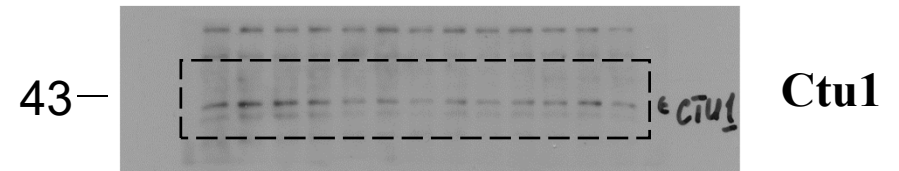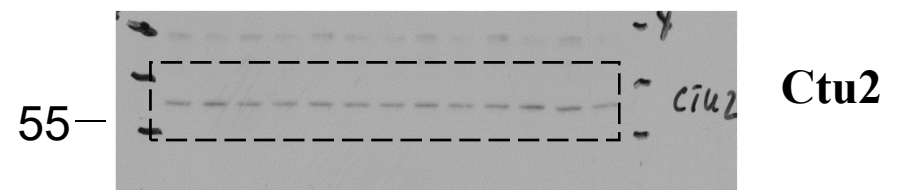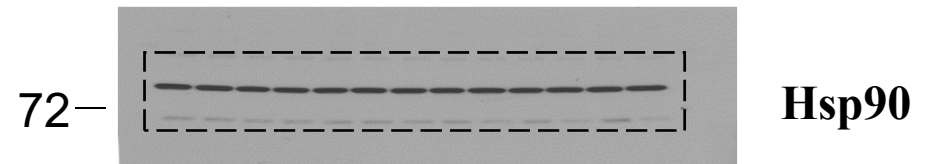

EV.1C

UUC

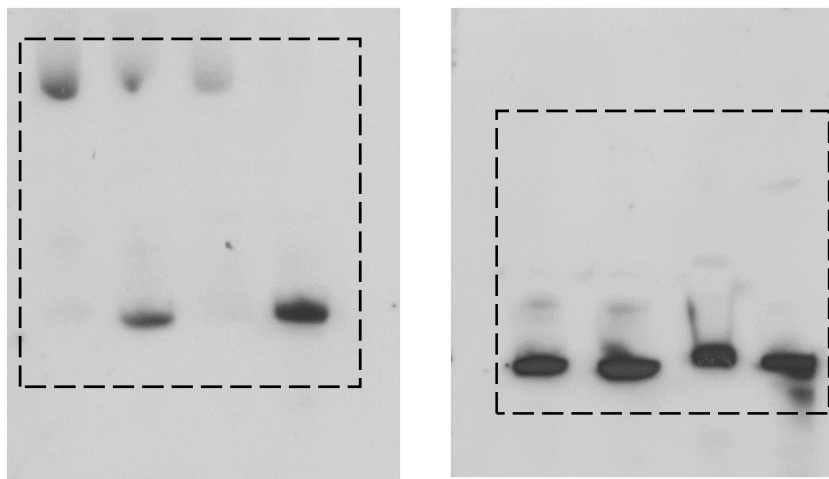

UUU

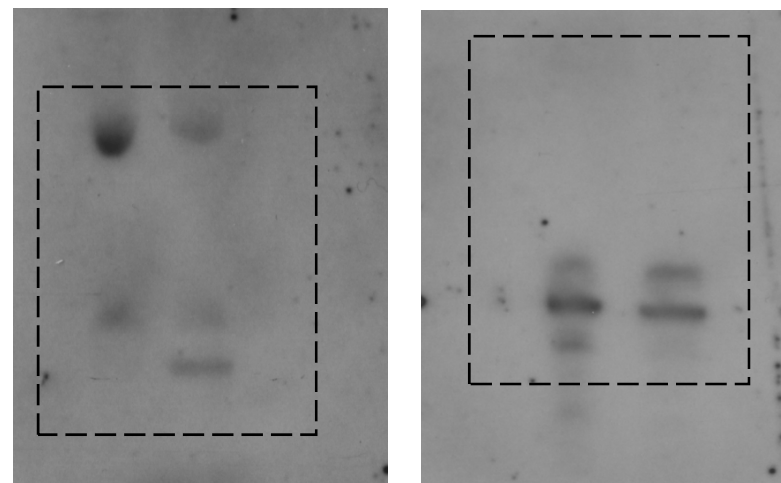

UUG

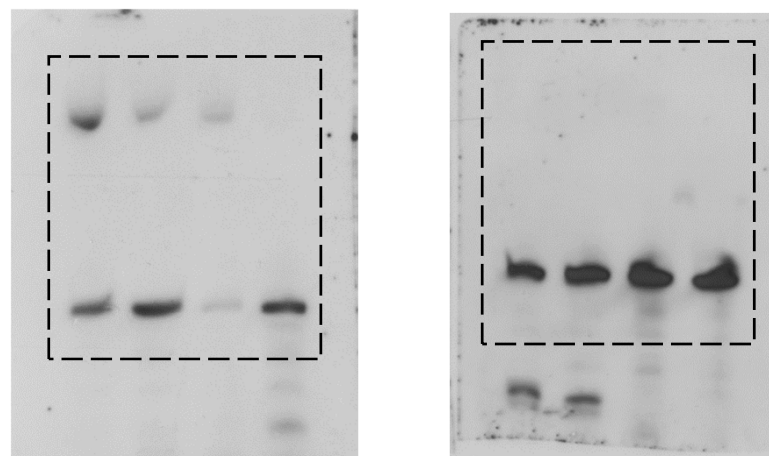

Supplement: Supplementary file 5 — Source Data for Expanded View [file EMBJ-41-e109353-s010.zip › Expanded figures/Source data_Expanded Figure 1.pdf]

**EV. 2B**

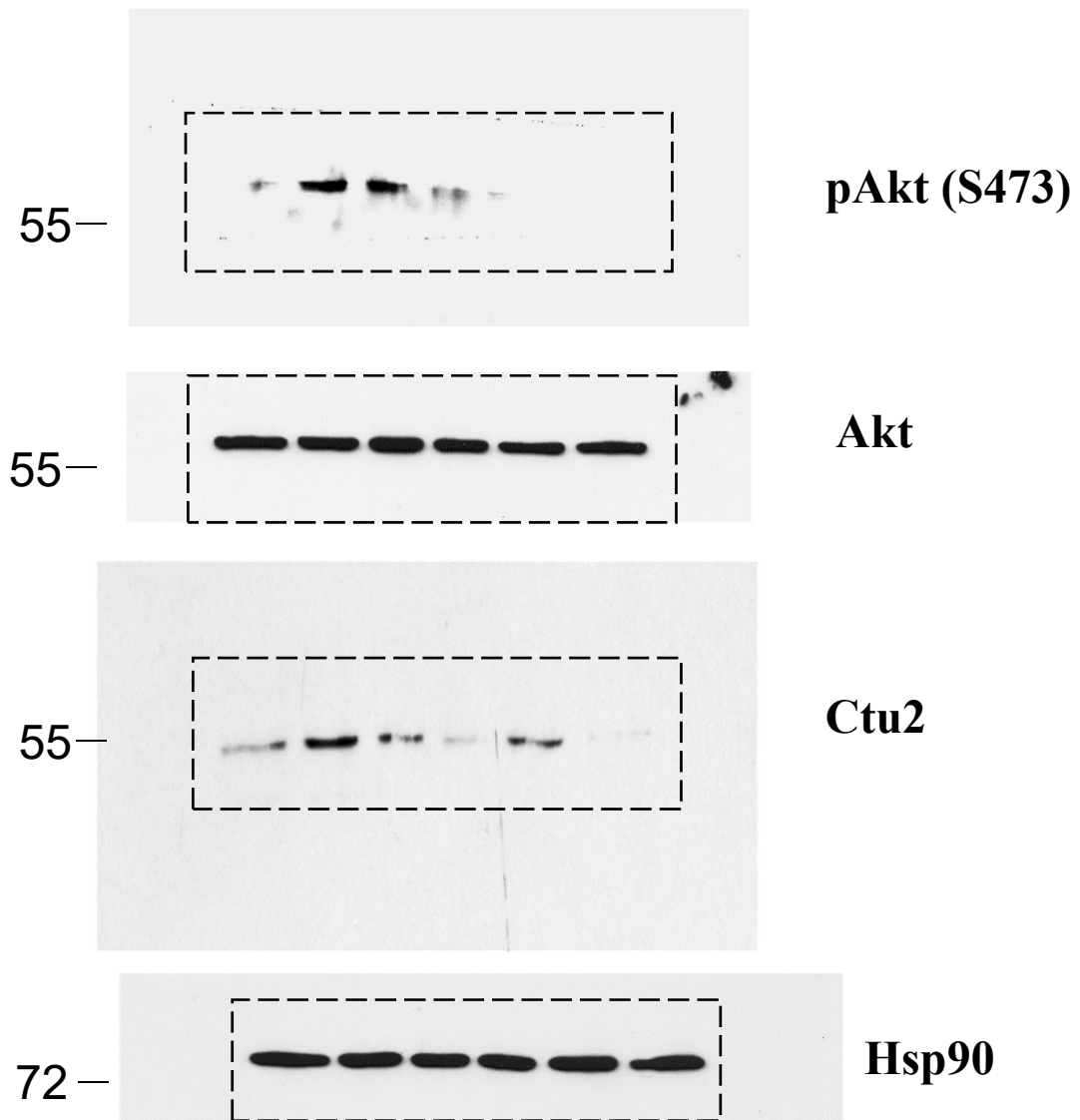

Supplement: Supplementary file 5 — Source Data for Expanded View [file EMBJ-41-e109353-s010.zip › Expanded figures/Source data_Expanded Figure 2.pdf]

## EV. 4B

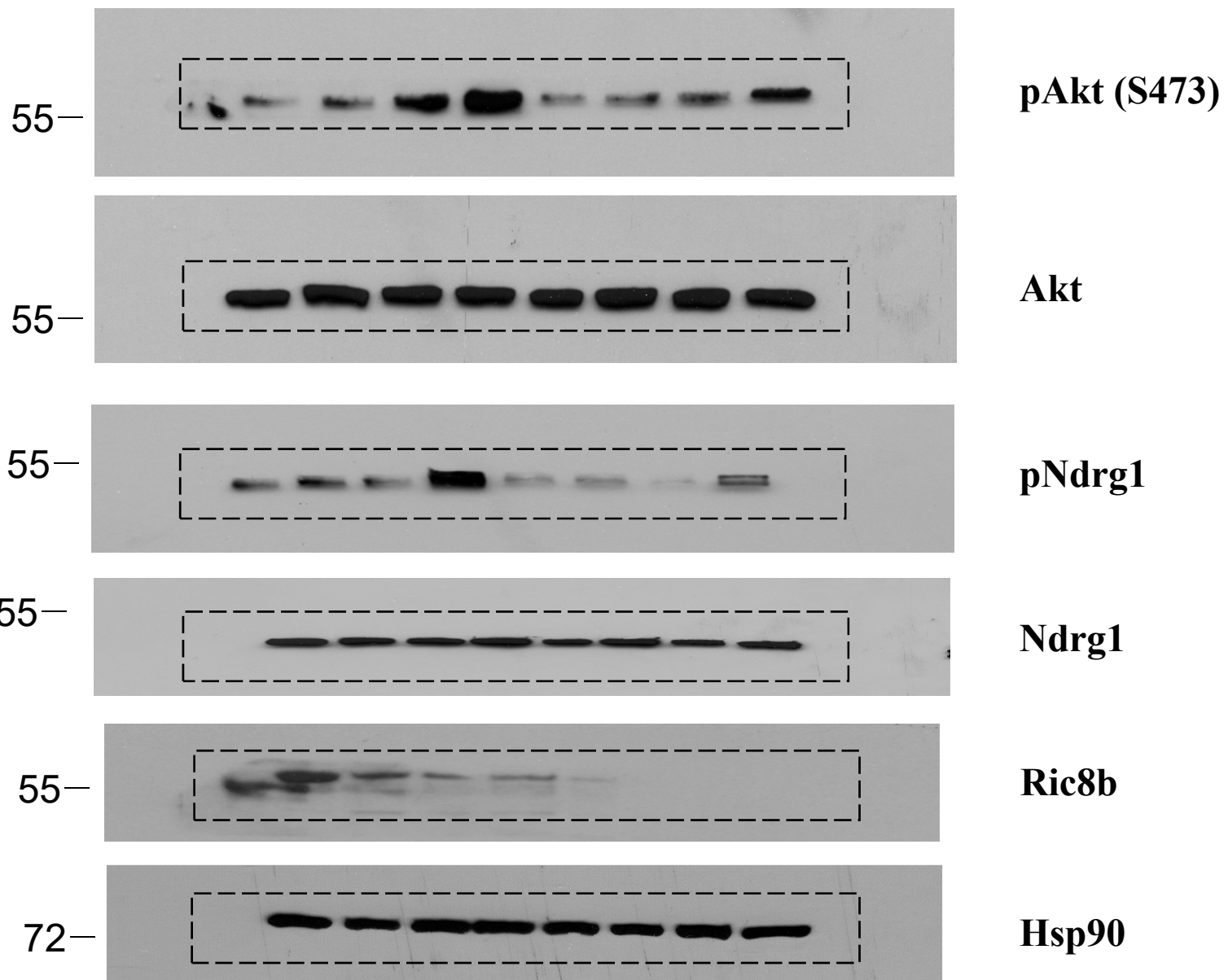

**EV. 4G**

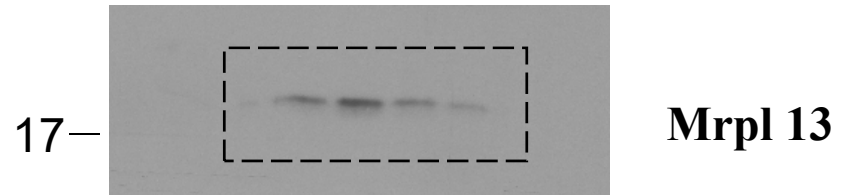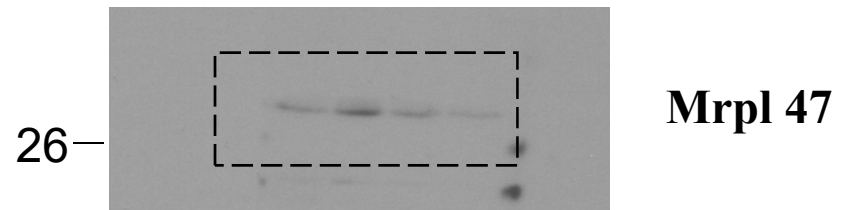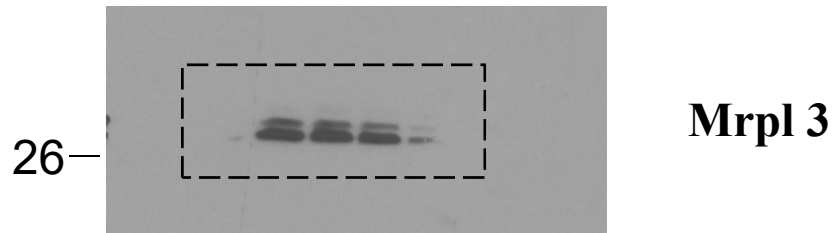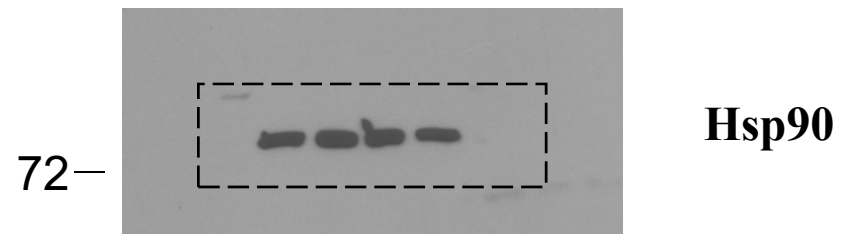

**EV. 4I**

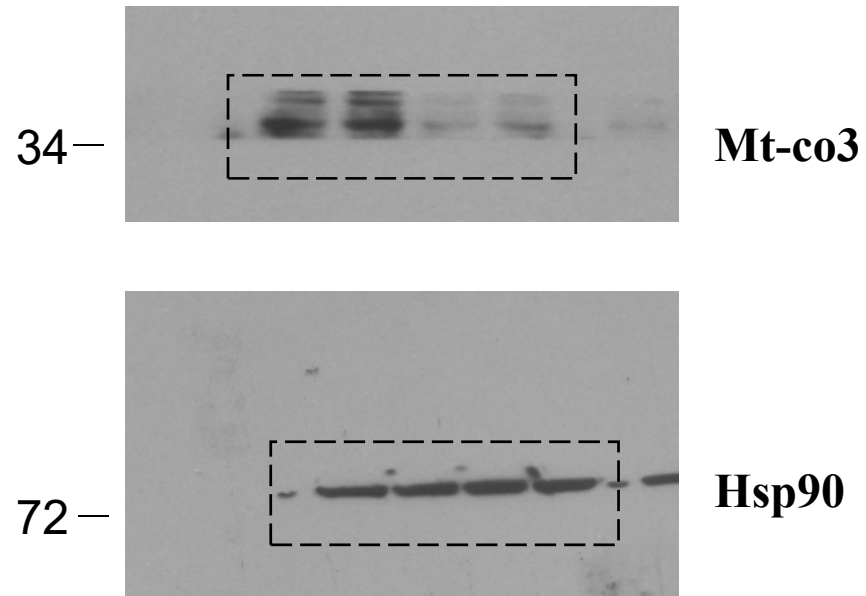

Supplement: Supplementary file 5 — Source Data for Expanded View [file EMBJ-41-e109353-s010.zip › Expanded figures/Source data_Expanded Figure 4.pdf]

**EV. 5A**

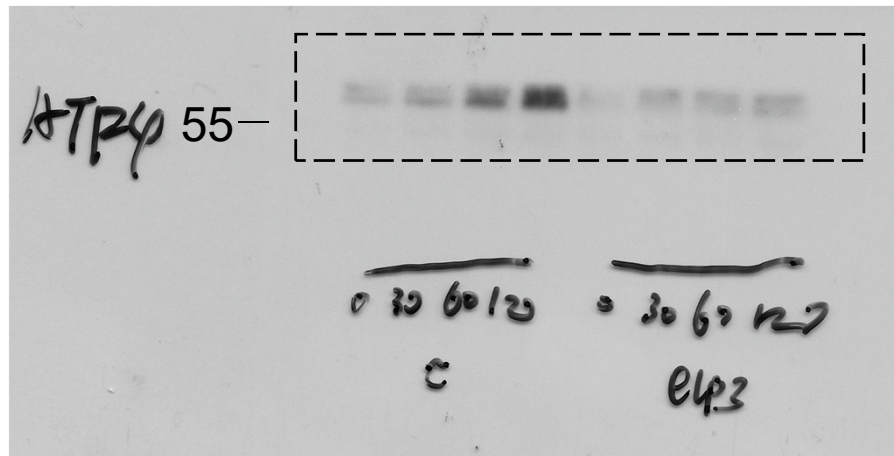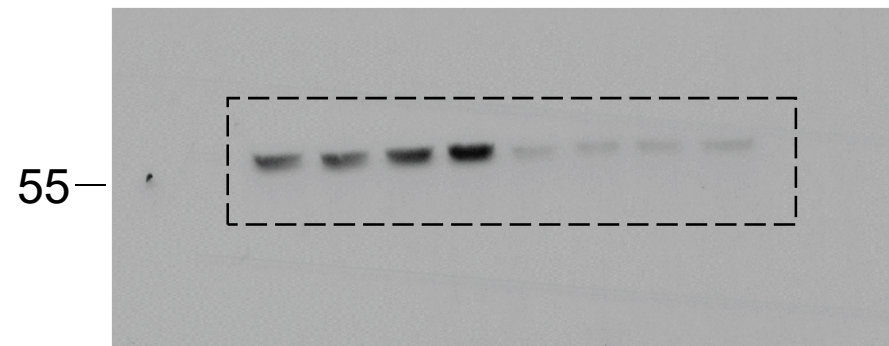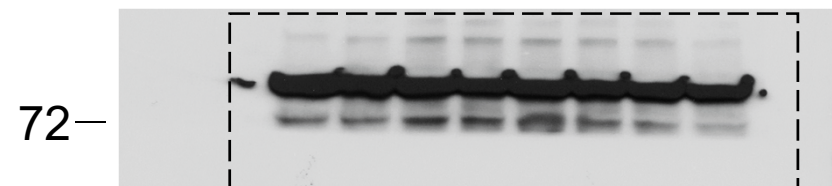

Supplement: Supplementary file 5 — Source Data for Expanded View [file EMBJ-41-e109353-s010.zip › Expanded figures/Source data_Expanded Figure 5.pdf]

**Figure 1A**

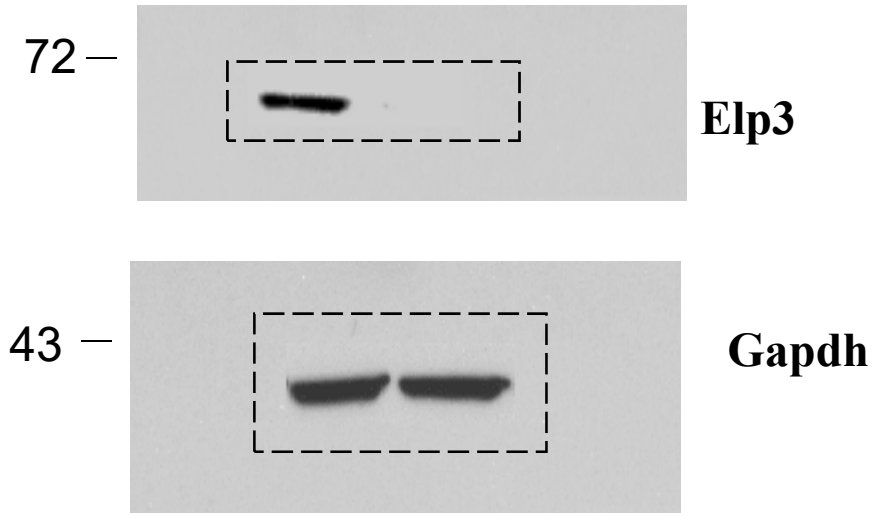

**Figure 1C**

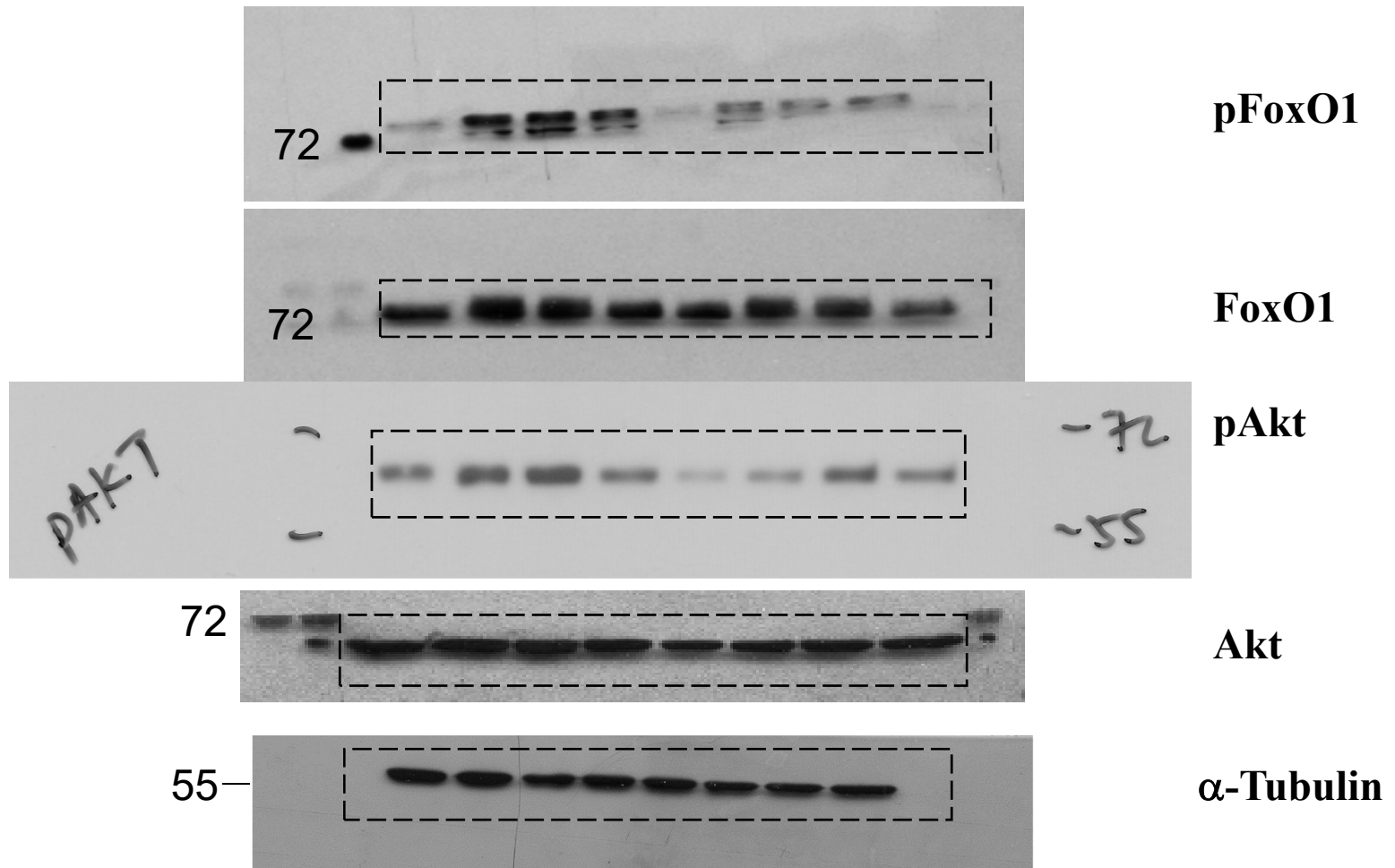

**Figure 1D**

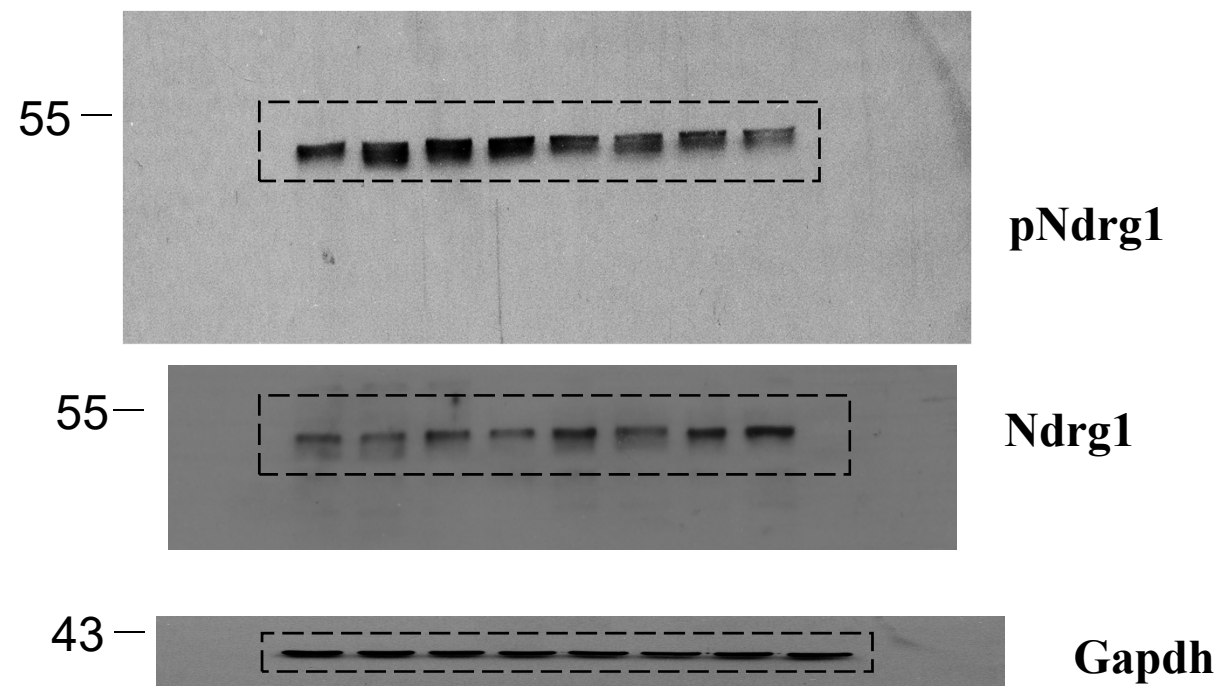

**Figure 1F**

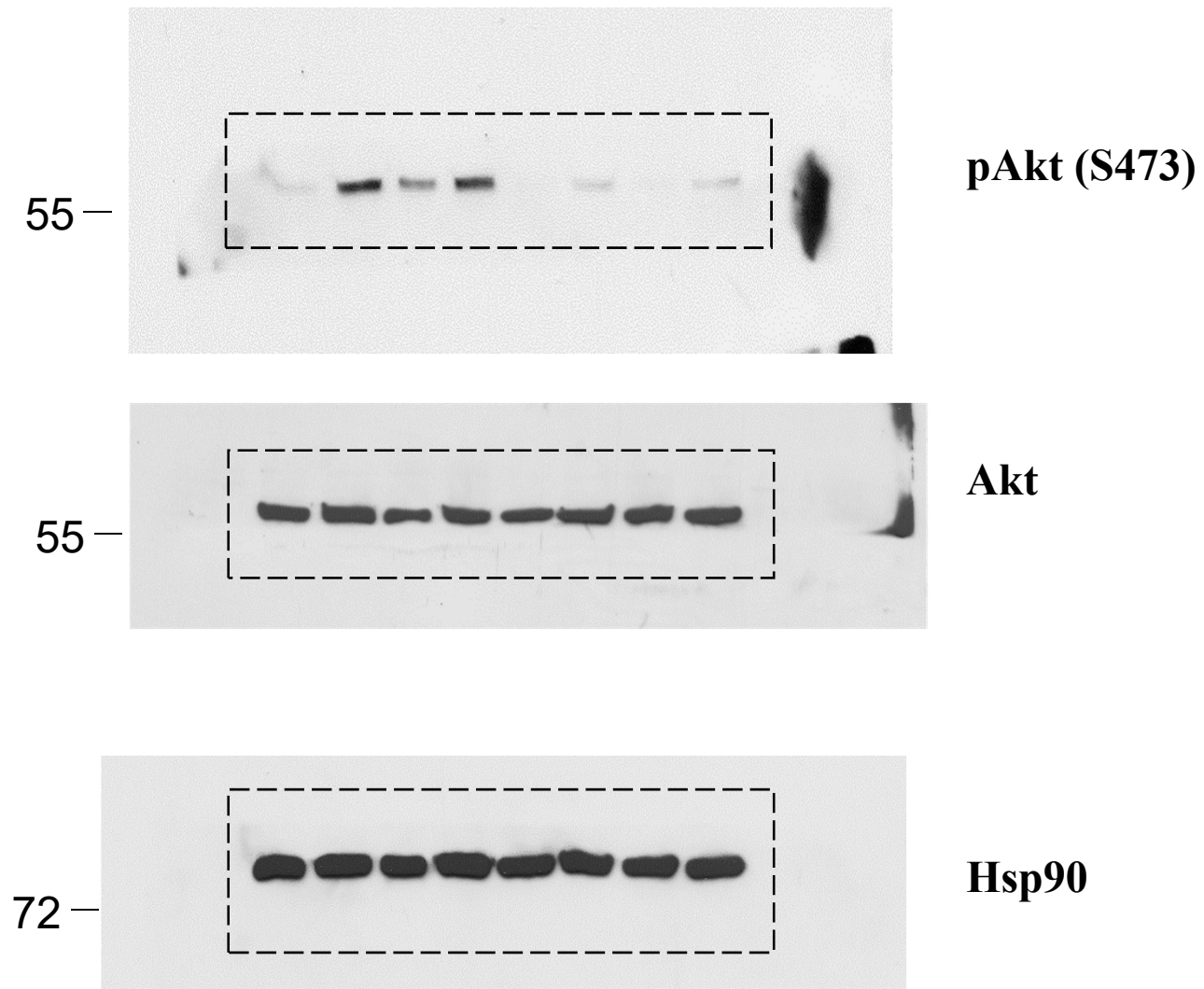

Supplement: Supplementary file 7 — Source Data for Figure 1 [file EMBJ-41-e109353-s011.pdf]

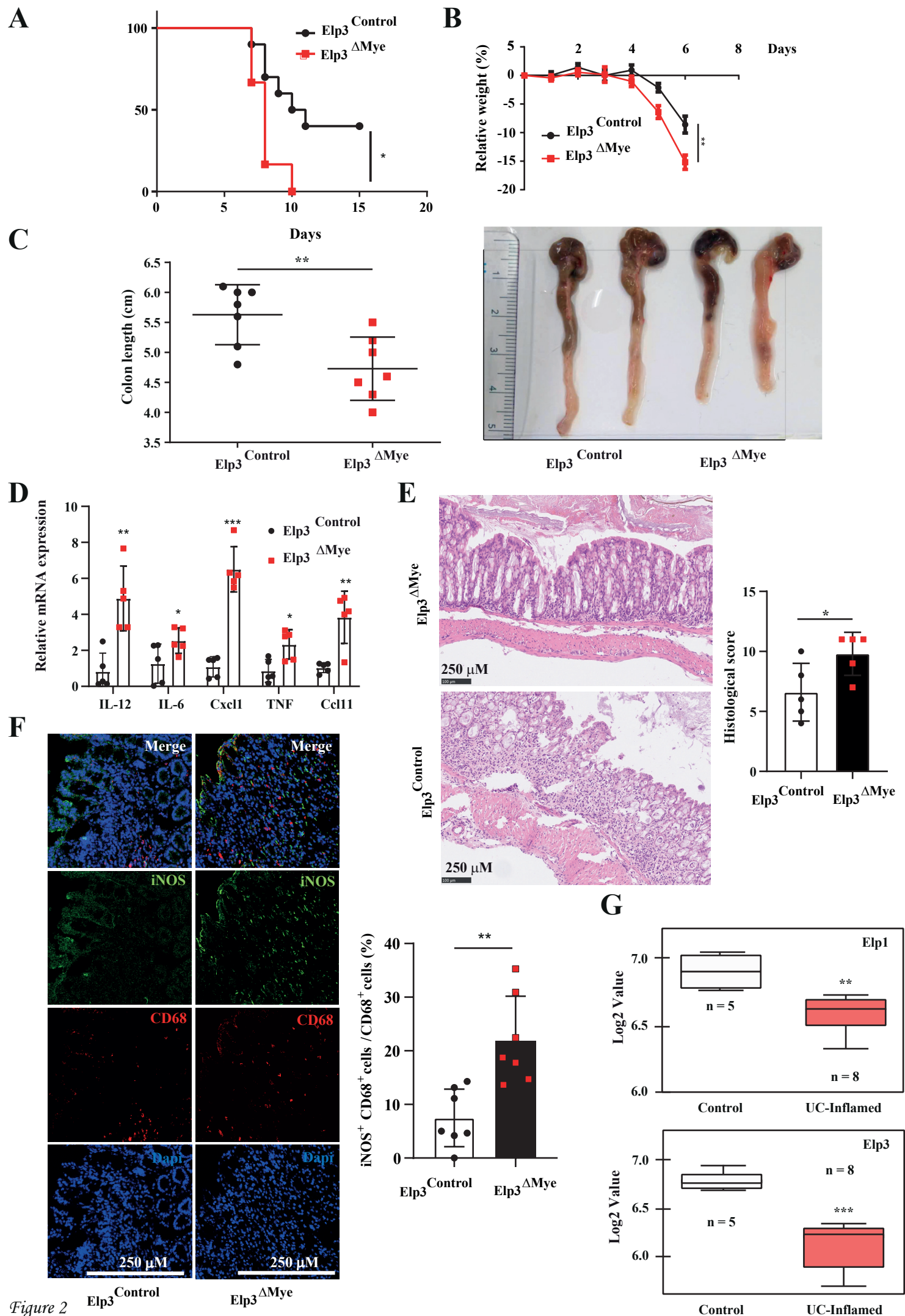

Figure 2

Supplement: Supplementary file 8 — Source Data for Figure 2 [file EMBJ-41-e109353-s002.pdf]

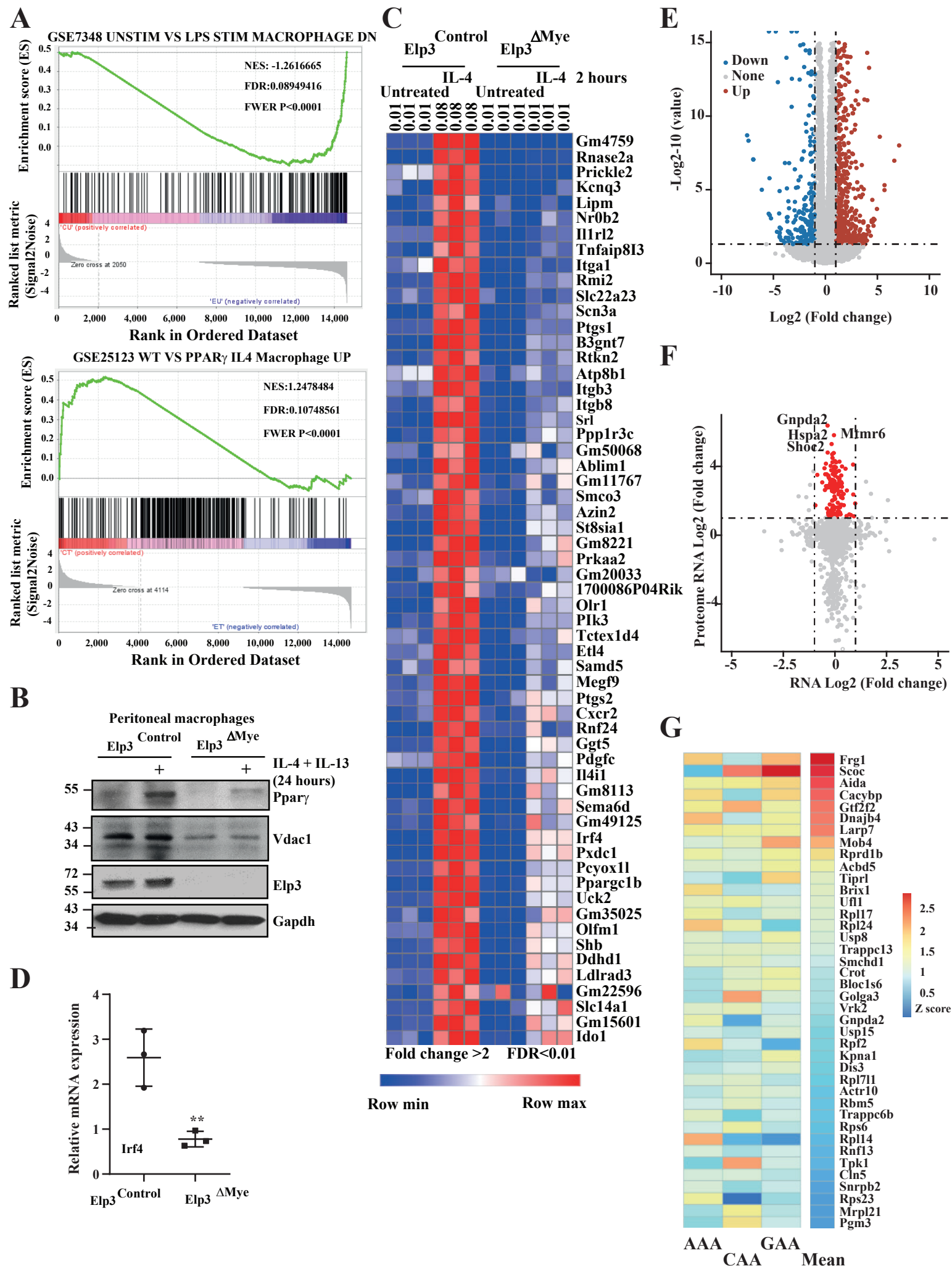

Figure 4

Supplement: Supplementary file 10 — Source Data for Figure 4 [file EMBJ-41-e109353-s009.pdf]

**Figure 7A**

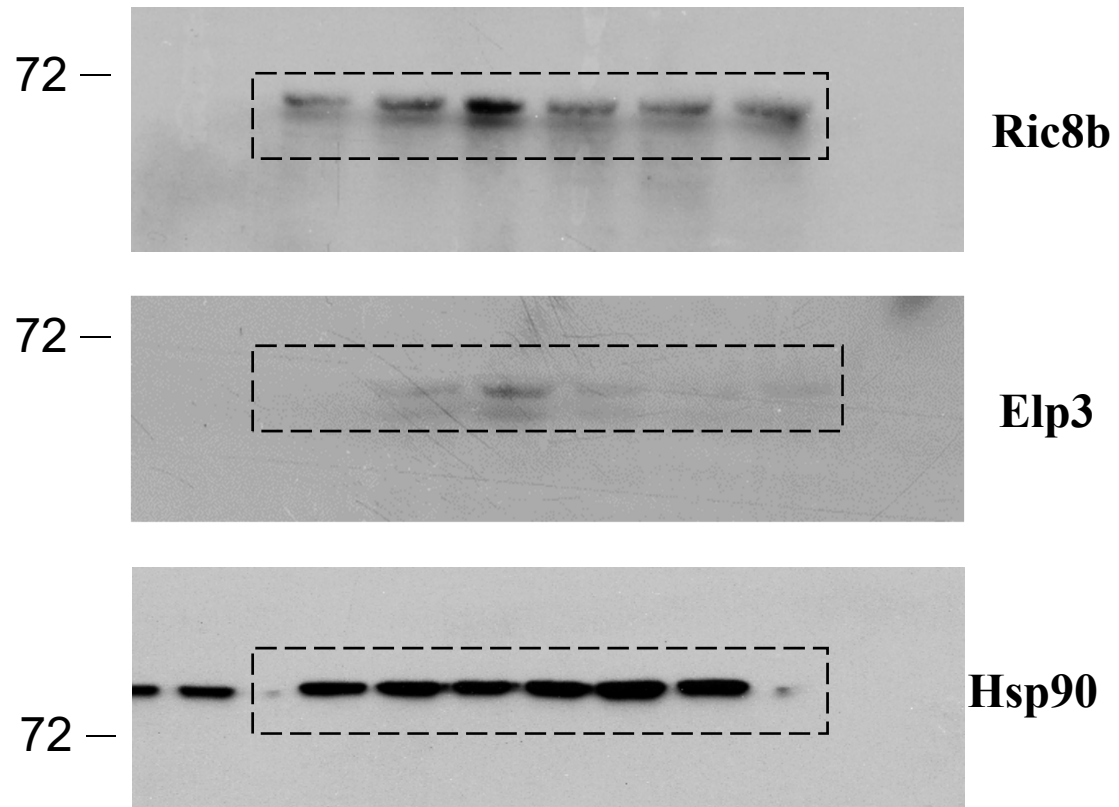

**Figure 7C**

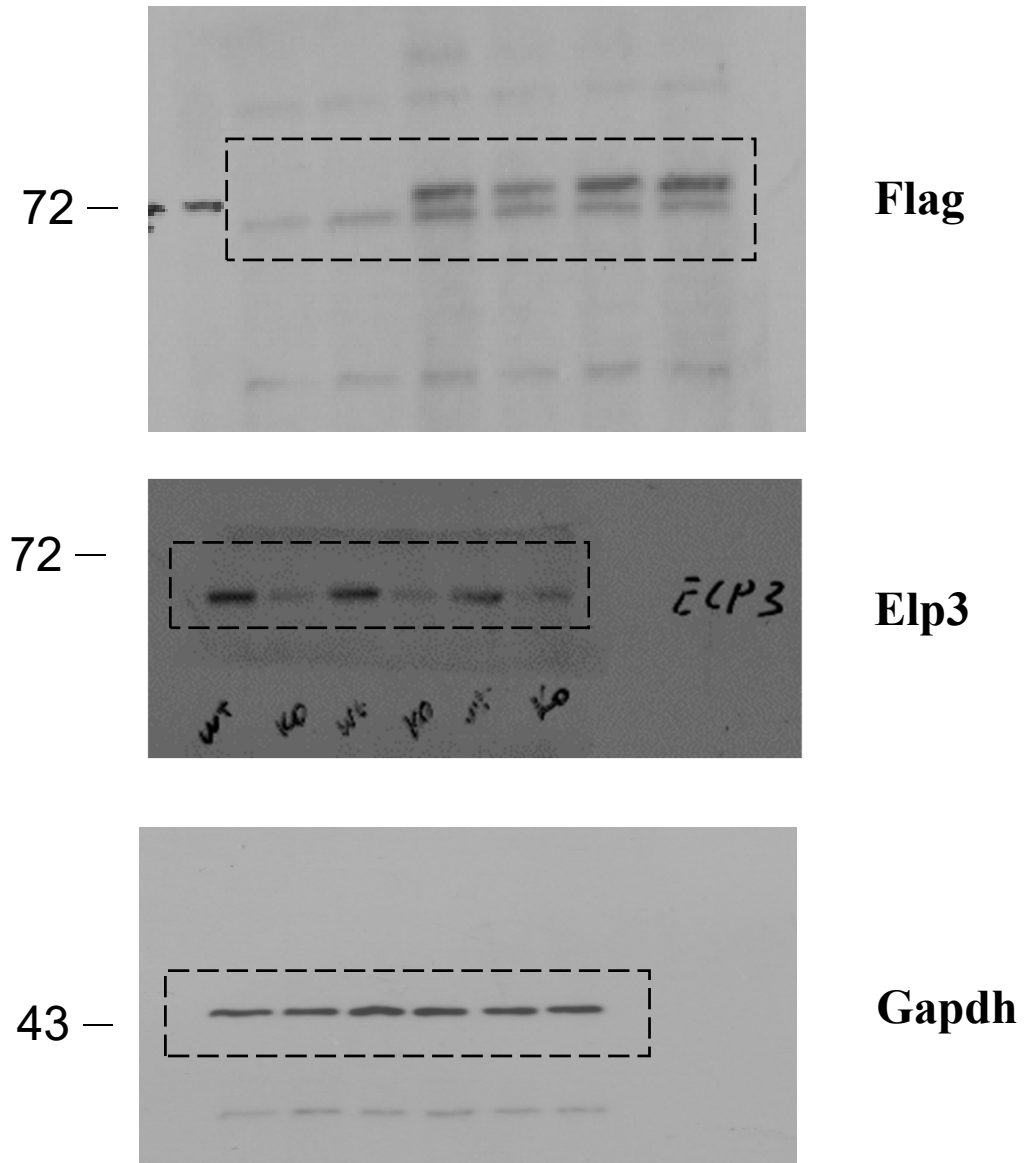

**Figure 7D**

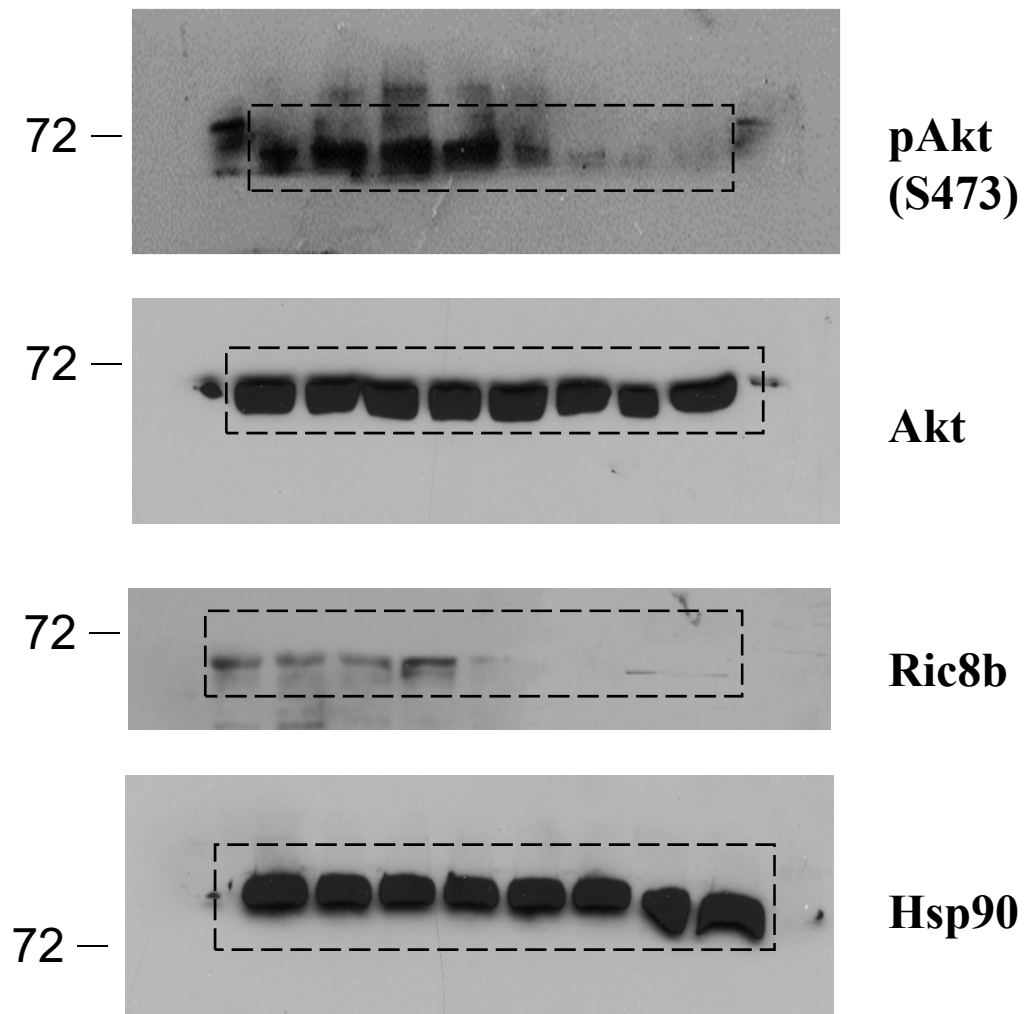

**Figure 7E**

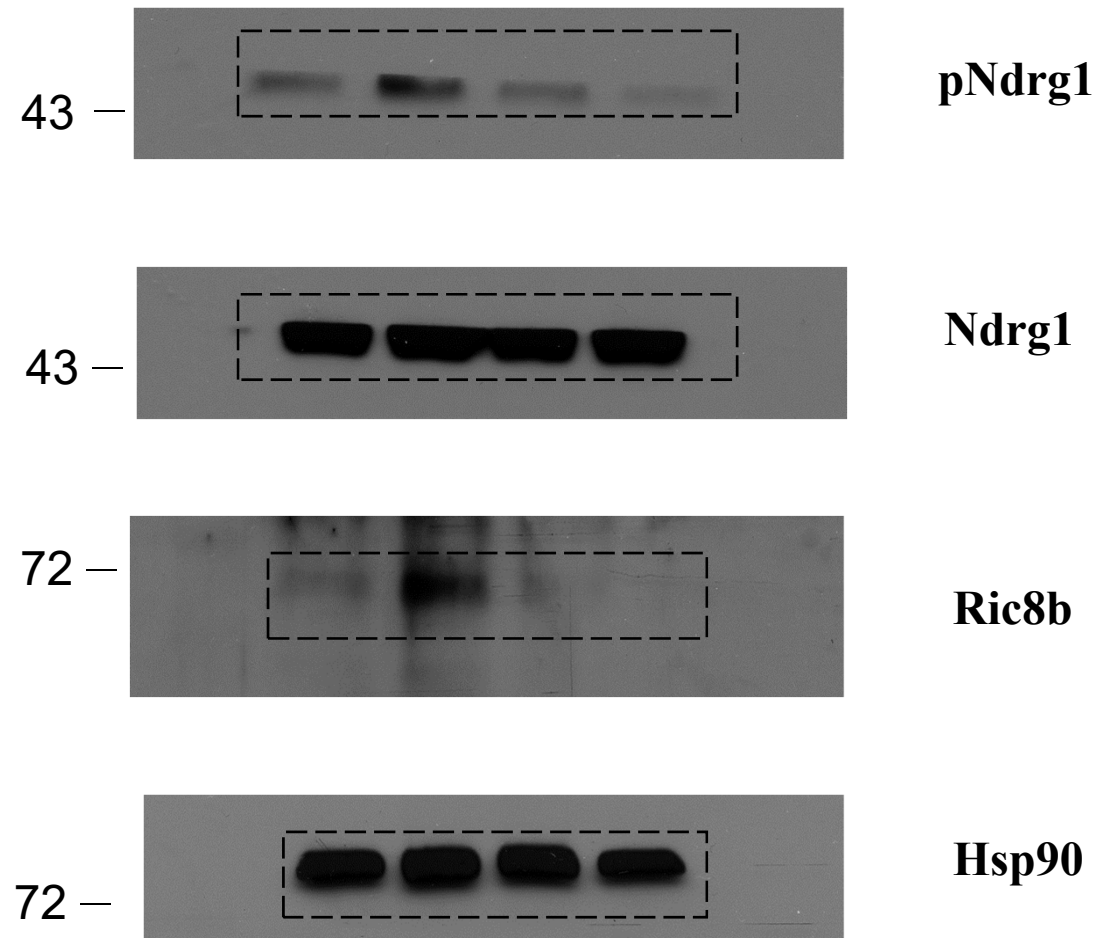

Supplement: Supplementary file 11 — Source Data for Figure 7 [file EMBJ-41-e109353-s006.pdf]

**Figure 8C**

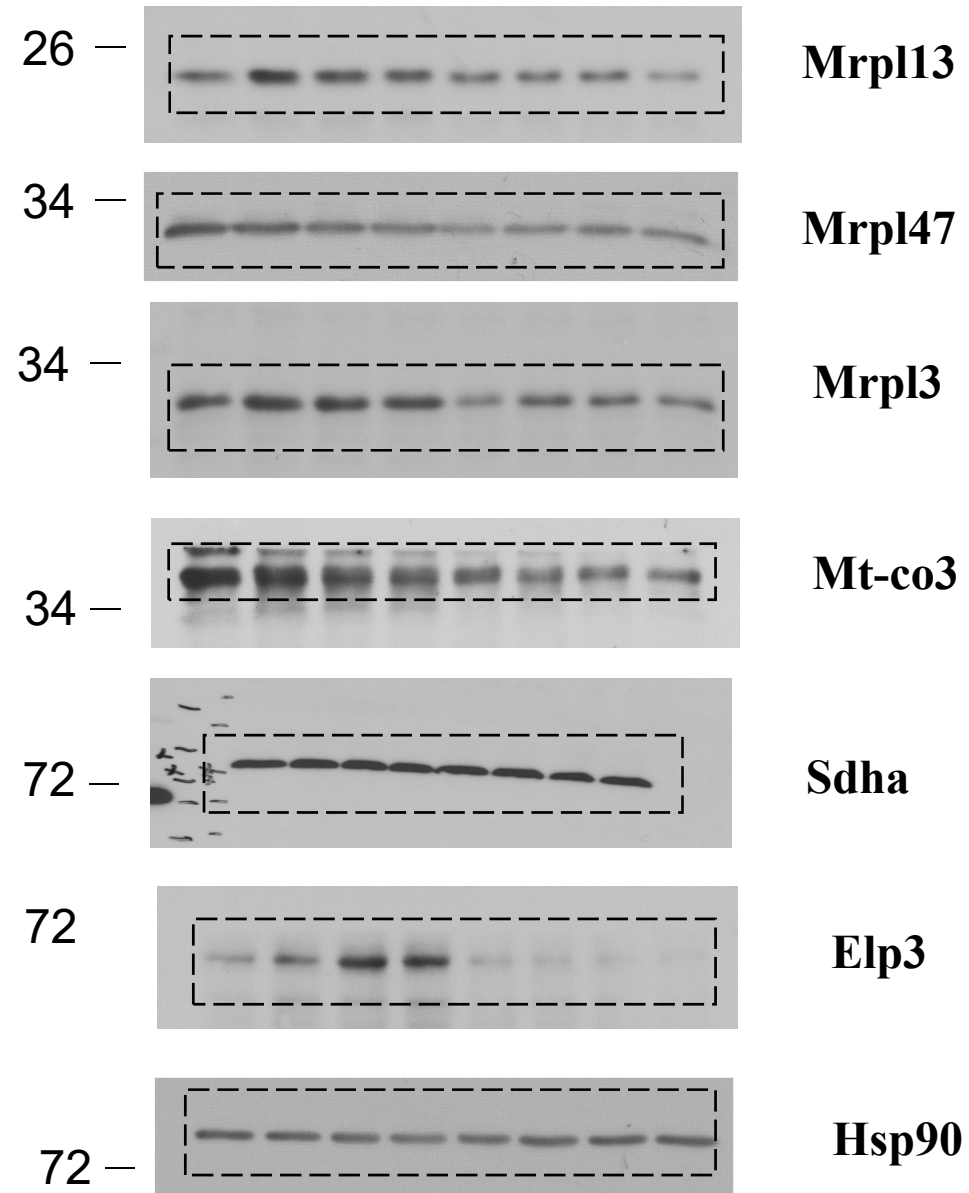

Supplement: Supplementary file 12 — Source Data for Figure 8 [file EMBJ-41-e109353-s005.pdf]

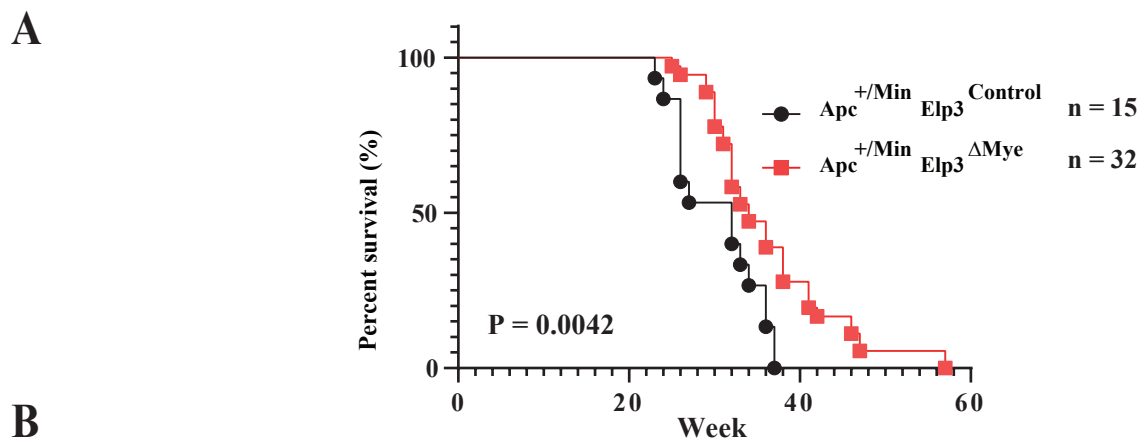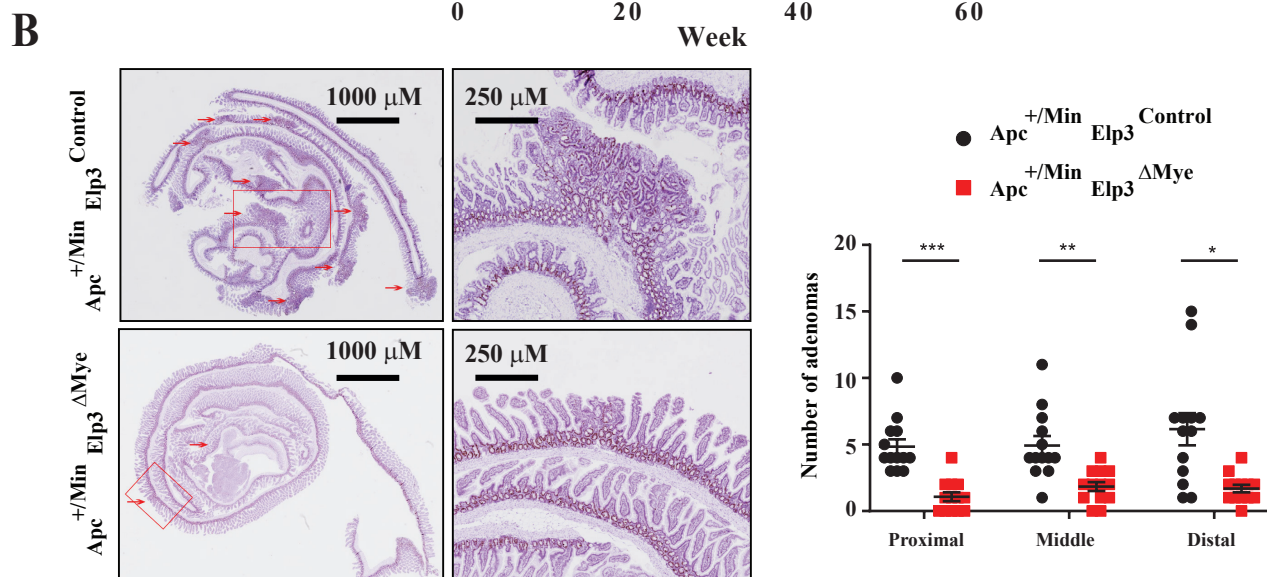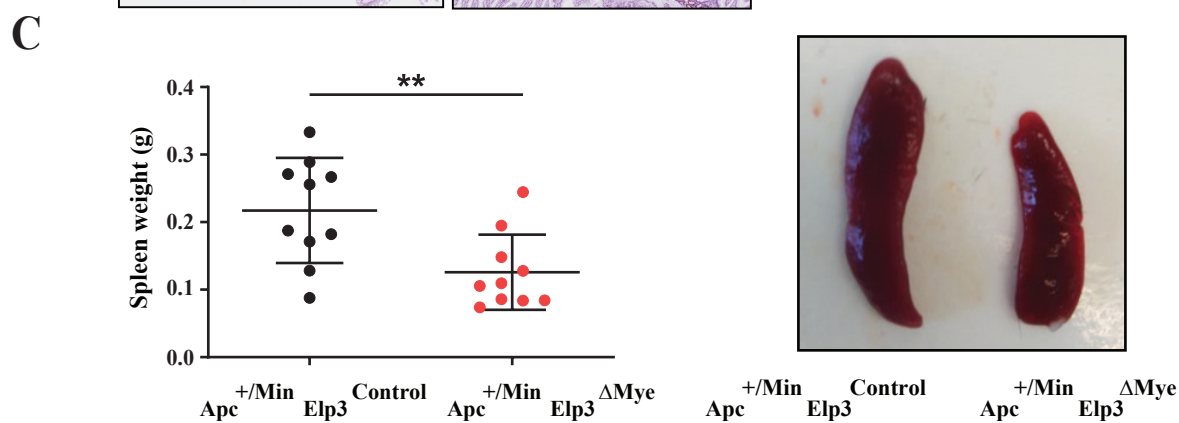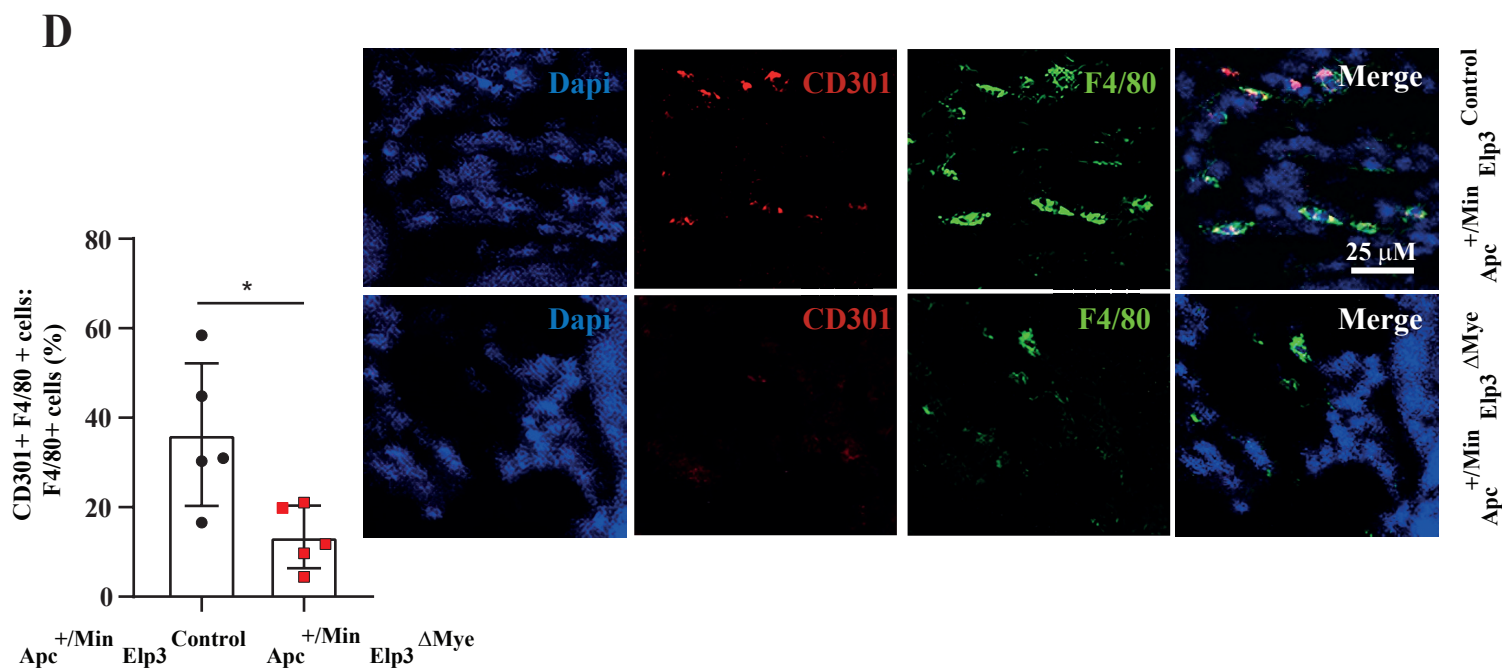

Figure 9

Supplement: Supplementary file 13 — Source Data for Figure 9 [file EMBJ-41-e109353-s012.pdf]
