# Supplementary material for: Elp3‐mediated codon‐dependent translation promotes mTORC2 activation and regulates macrophage polarization
Source: EMBO J. 2022 Aug 3;41(18):e109353. doi: 10.15252/embj.2021109353 (PMC9475509; doi:10.15252/embj.2021109353)
Supplement: Supplementary file 6 — PDF+ [file EMBJ-41-e109353-s003.pdf]

Article

SOURCE DATA | TRANSPARENT PROCESS | OPEN ACCESS

THE EMBO JOURNAL

Elp3-mediated codon-dependent translation promotes mTORC2 activation and regulates macrophage polarization

Dawei Chen[1,2,3], Ivan Nemazanyy[4], Olivier Peulen[1,5], Kateryna Shostak[1,2,3], Xinyi Xu[1,2,3], Seng Chuan Tang[1,2,3], Caroline Wathieu[1,2,3], Silvia Turchetto[1,3,6], Sylvia Tielens[1,2,3], Laurent Nguyen[1,3,6], Pierre Close[1,3,7,8], Christophe Desmet[1,9], Sebastian Klein[10], Alexandra Florin[10], Reinhard Büttner[10], Georgios Petrellis[11], Benjamin Dewals[11,†] & Alain Chariot[1,2,3,8,*,†]

Abstract

Macrophage polarization is a process whereby macrophages acquire distinct effector states (M1 or M2) to carry out multiple and sometimes opposite functions. We show here that translational reprogramming occurs during macrophage polarization and that this relies on the Elongator complex subunit Elp3, an enzyme that modifies the wobble uridine base U34 in cytosolic tRNAs. Elp3 expression is downregulated by classical M1-activating signals in myeloid cells, where it limits the production of pro-inflammatory cytokines via FoxO1 phosphorylation, and attenuates experimental colitis in mice. In contrast, alternative M2-activating signals upregulate Elp3 expression through a PI3K- and STAT6-dependent signaling pathway. The metabolic reprogramming linked to M2 macrophage polarization relies on Elp3 and the translation of multiple candidates, including the mitochondrial ribosome large subunit proteins Mrpl3, Mrpl13, and Mrpl47. By promoting translation of its activator Ric8b in a codon-dependent manner, Elp3 also regulates mTORC2 activation. Elp3 expression in myeloid cells further promotes Wnt-driven tumor initiation in the intestine by maintaining a pool of tumor-associated macrophages exhibiting M2 features. Collectively, our data establish a functional link between tRNA modifications, mTORC2 activation, and macrophage polarization.

Keywords Elp3; macrophage polarization; mitochondrial translation; mTORC2; tRNA modifications

Subject Categories Immunology; RNA Biology

DOI 10.15252/embj.2021109353 | Received 2 August 2021 | Revised 5 July 2022 | Accepted 7 July 2022 | Published online 3 August 2022

The EMBO Journal (2022) 41: e109353

Introduction

Macrophages are critical in homeostasis processes, in anti-microbial, and inflammatory activity in host defense as well as in the resolution of inflammation and wound healing (Murray & Wynn, 2011). Their response to microenvironmental cues allows them to acquire distinct effector states in order to carry out multiple functions. Their plasticity helps macrophages to acquire tailored activities within tissues and is often addressed through the concept of macrophage polarization (Pulendran & Artis, 2012; Murray *et al*, 2014; Murray, 2017). Interferon-γ (IFN-γ) is the canonical type 1 cytokine which, together with bacterial lipopolysaccharide (LPS), is associated with a type 1 immune environment and triggers a proinflammatory profile that is referred to as "M1" or "classical" macrophage polarization. *In vivo*, macrophages with M1 characteristics secrete a variety of pro-inflammatory cytokines (TNF, IL-1β, or IL-6) and cytotoxic molecules (reactive oxygen species and nitric oxide) to activate other immune cells and to carry out their microbicidal activity and anti-tumor immune responses (Murray *et al*, 2014). In contrast, interleukin 4 (IL-4) and IL-13, which are cytokines typically associated with type 2 immune responses, trigger

1 Interdisciplinary Cluster for Applied Genoproteomics, University of Liege, Liege, Belgium
2 Laboratory of Medical Chemistry, University of Liege, Liege, Belgium
3 GIGA Stem Cells, University of Liege, Liege, Belgium
4 Platform for Metabolic Analyses, Structure Fédérative de Recherche Necker, INSERM US24/CNRS UMS 3633, Paris, France
5 Metastasis Research Laboratory (MRL), GIGA Cancer, University of Liege, Liège, Belgium
6 Laboratory of Molecular Regulation of Neurogenesis, University of Liege, Liege, Belgium
7 Laboratory of Cancer Signaling, University of Liege, Liege, Belgium
8 Walloon Excellence in Life Sciences and Biotechnology (WELBIO), Wavres, Belgium
9 Laboratory of Cellular and Molecular Immunology, GIGA-I[3], University of Liege, Liège, Belgium
10 Institute for Pathology-University Hospital Cologne, Köln, Germany
11 Laboratory of Immunology-Vaccinology, Fundamental and Applied Research in Animals and Health (FARAH), University of Liege, Liège, Belgium
*Corresponding author. Tel: +32 (0) 4 366 24 72; E-mail: alain.chariot@uliege.be
[†]These authors contributed equally to this work

© 2022 The Authors. Published under the terms of the CC BY 4.0 license.

different responses in macrophages, resulting in what is often referred to as M2 macrophage polarization (Stein *et al*, 1992; Pulendran & Artis, 2012; Murray *et al*, 2014). M2 macrophages downplay inflammation, promote tissue repair and remodeling through the secretion of anti-inflammatory cytokines (IL-10), scavenging receptors (mannose receptor) and a variety of remodeling factors (Gordon & Martinez, 2010). Processes linked to M2 macrophage *in vivo* are notably involved in tumor growth and metastasis, in the cellular response to helminth infections as well as in insulin sensitivity in adipocytes (Lumeng *et al*, 2007; Gordon & Martinez, 2010). This initial classification was based on *in vitro* studies but it is now increasingly clear that macrophages display a more dynamic and varied spectrum of activation states in between the two extreme M1 and M2 classes, yet mechanisms by which macrophage polarization is regulated at the level of mRNA translation is poorly understood.

Cellular metabolism has emerged as an important regulator of macrophage function as signals involved in macrophage polarization cause major changes in their metabolic program (Saha *et al*, 2017). M1 macrophage polarization is associated with enhanced glycolysis, defective tricarboxylic acid (TCA) cycle and mitochondrial oxidative phosphorylation (OXPHOS) while M2 macrophages rely on TCA cycle and OXPHOS for energy generation (Vats *et al*, 2006; Rodríguez-Prados *et al*, 2010; O'Neill, 2016; Saha *et al*, 2017; Ryan & O'Neill, 2020).

The mechanistic target of Rapamycin (mTOR), a serine/threonine protein kinase, acts as a nutrient/energy sensor which couples nutrient availability to metabolic processes and critically regulates macrophage polarization (Düvel *et al*, 2010; Howell & Manning, 2011; Laplante & Sabatini, 2012; Byles *et al*, 2013). mTOR is part of two distinct complexes, mTORC1 and mTORC2, each complex having specific functions in cell signaling and metabolism (Liu & Sabatini, 2020). Myeloid-specific deficiency of *Rictor*, a specific adaptor molecule of mTORC2, blocks M2 polarization and impairs the cellular response to helminth infection (Hallowell *et al*, 2017). *Rictor*-deficient myeloid cells induce M1 macrophage polarization, similarly to the genetic deletion of *Tsc1*, a negative regulator of mTORC1, and consequently produce more inflammatory cytokines in response to LPS (Byles *et al*, 2013; Festuccia *et al*, 2014). Therefore, mTORC2 activation, linked to metabolic reprogramming, is critical in M2 macrophage polarization (Huang *et al*, 2016).

RNA modifications, which are defined as post-transcriptional changes in the chemical composition of RNA molecules, contribute to translational reprogramming and are involved in immune responses (Shulman & Stern-Ginossar, 2020). The most prevalent modified base found on messenger RNAs (mRNAs), N^6-methyladenosine (m^6A), regulate the antiviral response, at least through the modulation of interferon mRNAs (Rubio *et al*, 2018; Winkler *et al*, 2019). Although the majority of RNA modifications are found in noncoding RNAs, including transfer RNAs (tRNAs) and ribosomal RNAs, their biological relevance in immune responses remains to be elucidated. Among tRNA-modifying enzymes which target the tRNA anticodon, Elongator and Cytosolic thiouridylase (Ctu)-1/2 complexes exclusively modify the wobble uridine (U_{34}) base in cytosolic tRNAs (El Yacoubi *et al*, 2012). Ctu1, but not the associated Ctu2, harbors the enzymatic activity (Dewez *et al*, 2008). Elongator (Elp1-Elp6) includes Elp3, the catalytic subunit and Elp1, the scaffold protein, and promotes the formation of ^{5}-methoxycarbonylmethyl (mcm^5) or ^{5}-carbamoylmethyl (ncm^5) side chains on U_{34} while Ctu1/2 adds a ^{2}-thioylation (s^2) on U_{34} in the last step of this cascade. Only specific tRNAs (Arg^{UCU}, Lys^{UUU}, Gln^{UUG} and Glu^{UUC}) are targeted by both enzymes (El Yacoubi *et al*, 2012). In mice, *Elp3* deficiency impairs indirect neurogenesis and consequently leads to microcephaly, at least due to enhanced Atf4 levels (Laguesse *et al*, 2015). Elp3 expression in epithelial cells promotes Wnt-driven tumor initiation in the intestine and breast cancer metastasis, at least by promoting the expression of Sox9 and DEK in a codon-dependent manner, respectively (Ladang *et al*, 2015; Delaunay *et al*, 2016). Elp3 also promotes the acquired resistance to a B-RAF inhibitor in metastatic melanoma through HIF1α translation (Rapino *et al*, 2017). Mechanistically, Elp1 is activated by phosphorylation through an mTORC2-dependent pathway in melanoma cells (Rapino *et al*, 2018). In yeast, TORC2 also activates Elongator in a phospho-dependent manner while Elongator promotes the translation of key components of TORC2 (Candiracci *et al*, 2019). Therefore, the link between mTORC2 and some tRNA modifications seems conserved throughout evolution.

We show here that *Elp3* deficiency in myeloid cells potentiates the production of pro-inflammatory cytokines upon LPS stimulation, at least through decreased FoxO1 phosphorylation. As a result, *Elp3* deficiency exacerbates intestinal damage in a model of dextran sulfate sodium (DSS)-driven colitis. On the other hand, *Elp3* deficiency blocks IL-4-dependent M2 macrophage polarization. Mechanistically, *Elp3* deficiency impairs mTORC2 activation. Ric8b, a non-canonical guanine nucleotide exchange factor (GEF) acting as a positive regulator of mTORC2 (Nagai *et al*, 2020), relies on Elp3 to be properly translated in a codon-dependent manner. Both transcriptomic signature and metabolic reprogramming triggered by IL-4 is defective upon *Elp3* deficiency. Elp3 supports mitochondrial functions in metabolic reprogramming during macrophage polarization, at least through the production of some mitochondrial ribosome large subunit proteins. Finally, *Elp3* deficiency in myeloid cells delays Wnt-driven tumor initiation in the intestine by altering the pool of tumor-associated macrophages (TAMs). Therefore, Elp3 blocks M1 but favors M2 macrophage polarization. Collectively, our data establish a functional link between some tRNA modifications, mTORC2 activation, and macrophage polarization.

Results

Elp3 deficiency potentiates the differentiation into M1 macrophage

It is currently unknown whether Elongator regulates the polarization of monocytes into M1 or M2 macrophages. To address this issue, we first look at the expression of its subunits in macrophages undergoing M1 polarization. Elp3 and Elp1 expression at both mRNA and protein levels were downregulated by both LPS and IFNγ in mouse peritoneal macrophages (Fig EV1A and B). Of note, levels of Elp5 remained stable during M1 polarization while both Elp4 and Elp6 decreased, mostly at late time points (Fig EV1B). Interestingly, the thiolase Ctu1, but not Ctu2, was also downregulated by both IFNγ and LPS at the protein level (Fig EV1B). Therefore, downregulation of these tRNA-modifying enzymes is observed in cells undergoing differentiation into M1 macrophages.

To assess whether Elongator regulates the polarization of monocytes into M1 macrophages, we first inactivated *Elp3* in myeloid

 © 2022 The Authors

cells by breeding our *Elp3*$^{lox/lox}$ strain (Ladang *et al*, 2015) with the LysM-CRE mouse to generate the so-called « *Elp3*$^{\Delta Mye}$ » strain where Elp3 expression is abolished in myeloid cells (Fig 1A). As expected, levels of mcm^5S^2 U34 tRNA modifications quantified by Mass Spectrometry were decreased in myeloid cells lacking *Elp3* (Fig 1B). Consistently, the pool of thiolated tRNAs in BMDMs lacking Elp3 decreased, as assessed by Northern blots using UUC, UUG, and UUU probes (Fig EV1C). Similar experiments were conducted with total RNAs from intestinal epithelial cells (IECs) lacking or not *Elp3* as controls (Fig EV1C). We next isolated Thioglycollate-elicited peritoneal macrophages from both *Elp3*^{Control} and *Elp3*$^{\Delta Mye}$ strains and stimulated them *ex-vivo* with a combination of LPS and IFNγ to trigger M1 macrophage polarization. Akt phosphorylation on serine 473, a hallmark of mTORC2 activation, was defective upon *Elp3* deficiency (Fig 1C). Consistently, the LPS/IFNγ-dependent phosphorylation of Ndrg1, a mTORC2 substrate, was also defective upon *Elp3* deficiency (Fig 1D). Consistent with a defective Akt activation without Elp3, FoxO1, an Akt substrate (Brunet *et al*, 1999), was not properly phosphorylated in response to both LPS and IFNγ upon loss of Elp3 in peritoneal macrophages (Fig 1C). Therefore, mTORC2 activation relies on Elp3 expression in Thioglycollate-elicited peritoneal macrophages.

Inactivation of *Rictor* causes an hyperinflammatory phenotype in response to LPS, at least due to a defective Akt-dependent FoxO1 phosphorylation (Brown *et al*, 2011). Therefore, we reasoned that the defective FoxO1 phosphorylation seen upon *Elp3* deficiency may lead to enhanced macrophage-driven inflammation in response to LPS and IFNγ. To test this hypothesis, we assessed mRNA levels of several pro-inflammatory cytokines upon stimulation with IFNγ, LPS, or with a combination of both. mRNA levels of IL-6, IL-1β, and IL12p40 in peritoneal macrophages treated with LPS or with both LPS and IFNγ were significantly enhanced upon *Elp3* deficiency (Fig 1E). As Ctu1/2 act in the same enzymatic cascade as Elp3 (El Yacoubi *et al*, 2012), we next explored whether Ctu2 and Elp3 deficiencies induced a similar phenotype in M1 macrophage polarization. Ctu2 deficiency in bone marrow-derived macrophages (BMDMs) also impaired LPS/IFNγ-dependent Akt phosphorylation on S473 and potentiated the production of pro-inflammatory cytokines such as IL-6 and IL-1β upon stimulation with LPS/IFNγ (Fig 1F and G, respectively). Taken together, our data indicate that Elp3, and by extension U_{34} tRNA modifications, negatively regulates M1 macrophage polarization.

Elp3 deficiency in myeloid cells exacerbates experimental colitis

Pro-inflammatory M1 polarization of macrophages contributes to intestinal damage in mouse models of experimental colitis. As Elp3 limits M1 macrophage polarization, we reasoned that *Elp3* deficiency in myeloid cells would potentiate phenotypical parameters in a model of experimental colitis. To address this issue, we treated both *Elp3*^{Control} and *Elp3*$^{\Delta Mye}$ strains for 6 continuous days with Dextran sulfate sodium (DSS), which erodes the single layer of intestinal epithelial cells and causes colitis in mice. The survival time was decreased upon *Elp3* deficiency in myeloid cells (Fig 2A). Moreover, the weight loss due to DSS administration was more pronounced in *Elp3*$^{\Delta Mye}$ mice (Fig 2B). Importantly, colon length in *Elp3*$^{\Delta Mye}$ mice was reduced, reflecting a more severe phenotype following *Elp3* deficiency (Fig 2C). Consistently, mRNA levels of a variety of pro-inflammatory cytokines were more elevated in *Elp3*$^{\Delta Mye}$ mice as was the histological score upon treatment with DSS (Fig 2D and E, respectively). Moreover, the percentage of macrophages expressing M1 markers ($iNOS^+$/CD68$^+$ cells) in inflamed intestinal crypts was higher in *Elp3*$^{\Delta Mye}$ than in *Elp3*^{Control} mice subjected to DSS (Fig 2F). In agreement with the notion that *Elp3* deficiency is linked to an increased inflammatory status, patients suffering from ulcerative colitis showed lower levels of both Elp1 and Elp3 (Fig 2G). Of note, *Elp3* deficiency in myeloid cells did not impact the architecture of intestinal crypts, nor on both colon length and levels of pro-inflammatory cytokines (Fig EV1D–F, respectively). Therefore, Elp3 limits the inflammatory response in a model of experimental colitis, at least by blocking M1 macrophage polarization.

Figure 1. Elp3 negatively regulates M1 macrophage polarization.

A Generation of myeloid cells genetically inactivated for *Elp3*. Elp3 mRNA and protein levels were assessed by Real-Time PCR with extracts isolated from peritoneal macrophages of *Elp3*^{Control} and *Elp3*$^{\Delta Mye}$ mice. mRNA levels in Elp3^{Control} mice were set to 1 and levels in other experimental conditions were relative to that after normalization with Gapdh mRNA levels ($n = 3$ mice; mean ± SD, Student *t*-test, $***P < 0.001$). An anti-Elp3 western blot (WB) is also illustrated. The anti-Gapdh blot is shown for normalization purposes.

B Myeloid cells lacking Elp3 show decreased levels of mcm^5S^2 U34 tRNA modifications. Multiple tRNA modifications in BMDMs from both *Elp3*^{Control} and *Elp3*$^{\Delta Mye}$ mice were quantified by Mass Spectrometry (see Methods for details).

C, D Defective mTORC2 signaling upon *Elp3* deficiency in myeloid cells. Peritoneal macrophages from *Elp3*^{Control} and *Elp3*$^{\Delta Mye}$ mice were isolated and cultured *ex-vivo*. They were untreated or stimulated with a combination of LPS (100 ng/ml) and IFNγ (50 ng/ml) for the indicated periods of time and the resulting cell extracts were subjected to WB analyses.

E Enhanced mRNA levels of pro-inflammatory cytokines upon *Elp3* inactivation in myeloid cells subjected to LPS or to both LPS and IFNγ stimulations. Peritoneal macrophage from *Elp3*^{Control} or *Elp3*$^{\Delta Mye}$ mice were unstimulated (« Control ») or treated with IFNγ (50 ng/ml), LPS (100 ng/ml) or with both IFNγ and LPS for 24 h and the resulting mRNAs of the listed pro-inflammatory cytokines were assessed by Real Time PCR. mRNA levels in *Elp3*^{Control} mice were set to 1 and levels in other experimental conditions were relative to that after normalization with Gapdh mRNA levels ($n = 6$ mice; mean ± SD, Student *t*-test, $**P < 0.01$, $*P < 0.05$).

F mTORC2 activation by M1 polarization signals relies on Ctu2. Control and Ctu2-depleted bone marrow-derived macrophages (BMDMs; SiRNA-mediated depletions) were treated or not with IFNγ (50 ng/ml) and LPS (100 ng/ml) and the resulting cell extracts were subjected to WB analyses.

G Ctu2 deficiency potentiates M1 macrophage polarization. Control and Ctu2-depleted BMDMs were treated or not with IFNγ (50 ng/ml) and LPS (100 ng/ml) for 24 h and the resulting mRNAs of the listed pro-inflammatory cytokines were assessed by Real Time PCR. mRNA levels in control BMDMs were set to 1 and levels in other experimental conditions were relative to that after normalization with Gapdh mRNA levels. Experiments were conducted in triplicates (mean ± SD, Student *t*-test, $*P < 0.05$, $**P < 0.01$, $***P < 0.001$).

Source data are available online for this figure.

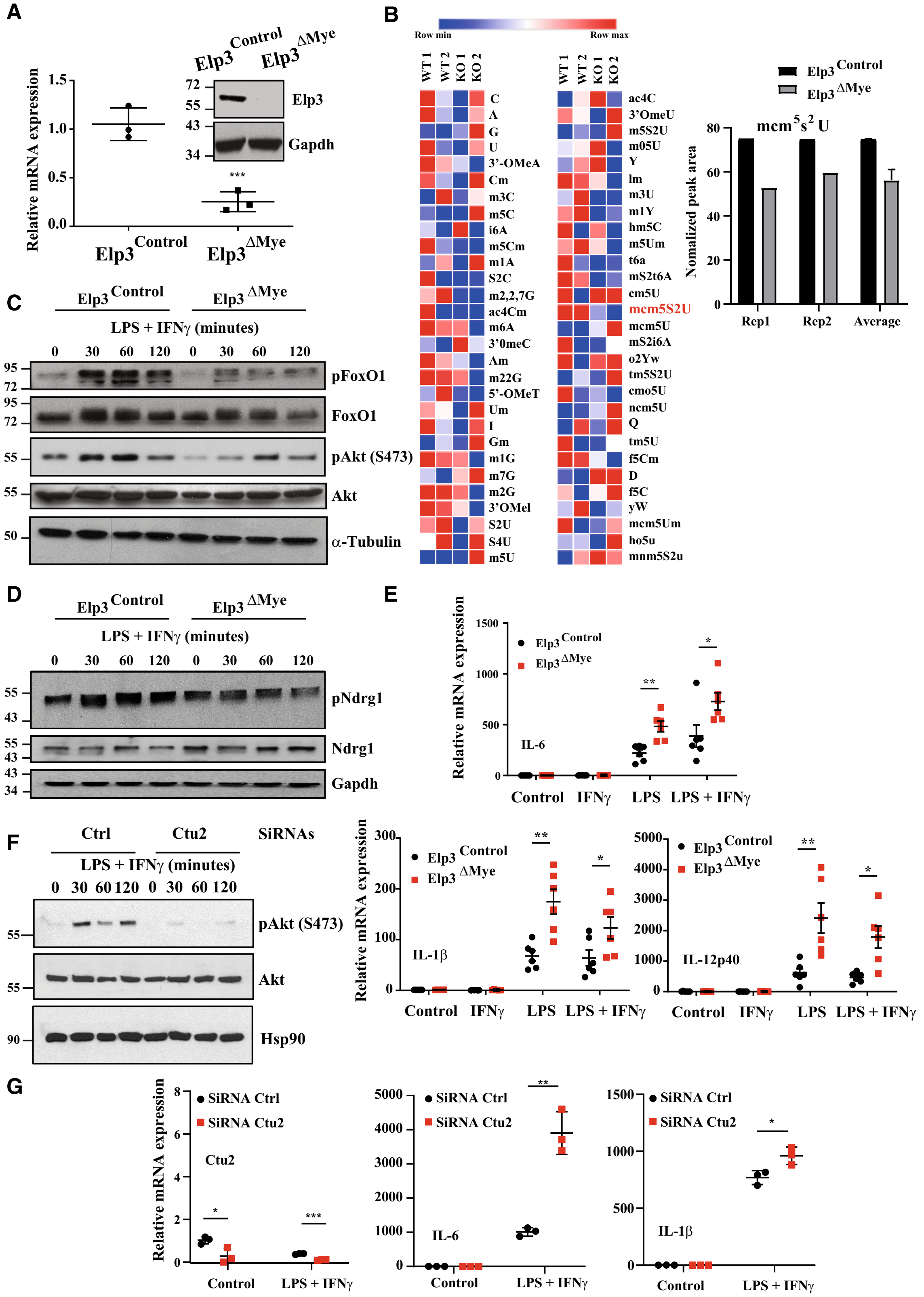

Figure 1.

© 2022 The Authors

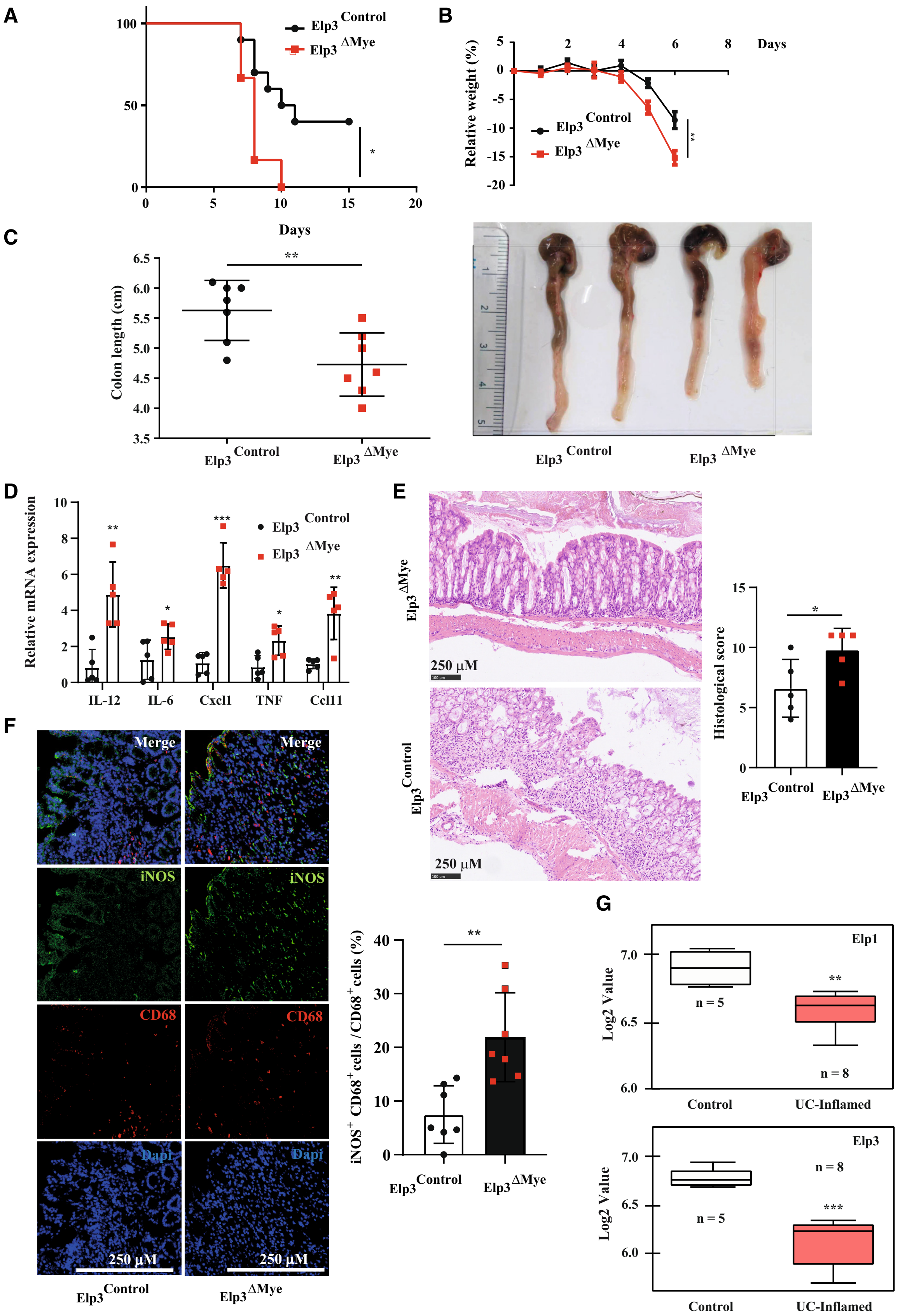
A
B
C
D
E
F
G
Elp3 Control
Elp3 ΔMye
Days
Relative weight (%)
Colon length (cm)
Relative mRNA expression
IL-12
IL-6
Cxcl1
TNF
Ccl11
Histological score
250 μM
Merge
iNOS
CD68
Dapi
iNOS+ CD68+ cells / CD68+ cells (%)
Log2 Value
Elp1
Elp3
n = 5
n = 8
Control
UC-Inflamed

Figure 2.

Figure 2. Elp3 expression in myeloid cells protects from intestinal damage in an experimental model of colitis.

A Decreased survival of mice lacking Elp3 expression in myeloid cells upon DSS administration (n = 10 mice per genotype; mean ± SD, Student t-test, *P < 0.05).

B, C *Elp3* deficiency in myeloid cells exacerbates the weight loss and the decrease of colon length upon DSS administration (n = 7 mice; mean ± SD, **P < 0.01).

D *Elp3* deficiency in myeloid cells potentiates the production of cytokines and chemokines upon DSS administration. mRNA levels of the indicated candidates in $Elp3^{Control}$ mice were set to 1 and levels in other experimental conditions were relative to that after normalization with Gapdh mRNA levels (n = 5 mice per genotype; mean ± SD, Student t-test, *P < 0.05, **P < 0.01, ***P < 0.001).

E More severe histological score in mice lacking Elp3 in myeloid cells upon DSS administration. Representative intestinal crypts from the indicated genotypes are illustrated. The histological score was established as described in methods (n = 5 mice per genotype; mean ± SD, Student t-test, *P < 0.05).

F Increased infiltration of $iNOS^+/CD68^+$ cells upon DSS administration in $Elp3^{\Delta Mye}$ mice. The percentage of $iNOS^+$ in $CD68^+$ cells was established in both genotypes treated with DSS (n = 7 mice per genotype; mean ± SD, Student t-test, **P < 0.01). Anti-iNOS and -CD68 immunofluorescence analyses carried out in intestinal crypts from both genotypes are illustrated.

G Patients suffering from ulcerative colitis (UC) show reduced levels of both Elp1 and Elp3. The central band represents the mean value of relative expression in the investigated cohort. Boxes represent 75th and 25th percentile. Whiskers represent maximum and minimum values before the upper and lower fence, respectively (**P < 0.01, ***P < 0.001; GSE9452-GEO DataSets, Definition of an ulcerative colitis preinflammatory state).

Source data are available online for this figure.

Elp3 expression is induced by IL-4 and IL-13 in macrophages

IL-4 and IL-13 trigger cell polarization into anti-inflammatory M2 macrophages. To assess whether Elp3 regulates M2 macrophage polarization, we first treated macrophages with IL-4, IL-13, or with both cytokines. These cytokines induced Elp3, Elp1, Ctu1, and Ctu2 mRNAs and protein levels in mouse peritoneal macrophages (Figs 3A and EV2A, respectively). Consistently, the pool of thiolated tRNAs increased upon IL-4/IL-13 induction in Thioglycollate-elicited peritoneal macrophages while this pool of chemically modified tRNAs decreased upon treatment with LPS/IFNγ (Fig 3B). Elp3 induction by both IL-4 and IL-13 occurred through a PI3K-dependent pathway as the PI3K inhibitor Wortmannin-blocked Elp3 induction by these cytokines in peritoneal macrophages (Fig 3C). Of note, Wortmannin had no effect on IL-4-dependent Elp3 induction at the mRNA level, indicating that its effect on Elp3 expression is post-transcriptional (Fig 3D). As mTOR is activated by IL-4 in macrophages (Byles *et al*, 2013), we next explored whether mTOR activation was required for Elp3 induction by IL-4. Rictor but not Raptor deficiency severely interfered with Elp3 expression in BMDMs, indicating that mTORC2 but not mTORC1 promotes Elp3 expression (Fig 3E). As the transcription factor Stat6 is also critical for gene induction by IL-4 (Hou *et al*, 1994), we next explored whether the pharmacological inhibition of Stat6 had any consequences on Elp3 expression. As expected, ASI517499 severely abolished Stat6 phosphorylation (Fig 3F). Moreover, this Stat6 inhibitor

Figure 3. Elp3 induction by IL-4 through multiple signaling pathways.

A IL-4 and IL-13 induce the expression of tRNA-modifying enzymes in macrophages. Peritoneal macrophages were untreated (« Control ») or stimulated with IL-4 (10 ng/ml), IL-13 (10 ng/ml) or with both cytokines for 24 h and cell extracts from the resulting cells were subjected to WB analyses.

B The pool of thiolated tRNAs in macrophages is enhanced by IL-4/IL-13 but decreased by LPS/IFNγ. On the left, Northern blot analysis assessing t:Q(UUG) tRNA thiolation in Thioglycollate-elicited peritoneal macrophages subjected to the indicated treatments for 24 h. On the right, quantification of t:Q(UUG) tRNA thiolation, calculated as the ration of thiolated over nonthiolated t:Q(UUG), in the indicated experimental conditions.

C Elp3 induction by IL-4/IL-13 occurs through a PI3K-dependent pathway. Peritoneal macrophages were pretreated with DMSO (control vehicle) or with Wortmannin (200 nM) for 1 h and subsequently left untreated or stimulated with IL-4 (10 ng/ml)/IL-13 (10 ng/ml) for the indicated periods of time. Cell extracts from the resulting cells were subjected to WB analyses.

D PI3K inhibition does not impact on Elp3 mRNA levels. Peritoneal macrophages were pretreated with DMSO (control vehicle) or with Wortmannin (200 nM) for 1 h and subsequently left untreated or stimulated with IL-4 (10 ng/ml) for 24 h. Elp3 mRNA levels were quantified in all experimental conditions by Real-Time PCR. Elp3 mRNA levels in unstimulated cells were set to 1 and levels in other experimental conditions were relative to that after normalization with Gapdh mRNA levels. Experiments were conducted in triplicates (mean ± SD, Student t-test, **P < 0.01).

E Rictor but not Raptor deficiency impairs Elp3 expression. BMDMs were transfected with the indicated siRNAs and the resulting cells were treated or not with IL-4 (10 ng/ml)/IL-13 (10 ng/ml) for 24 h. Cell extracts were subjected to WB analyses.

F IL-4/IL-13 rely on Stat6 to induce Elp3 expression. Peritoneal macrophages were pretreated with the control vehicle (DMSO) or with the Stat6 pharmacological inhibitor AS1517499 (5 μM) for 1 h and subsequently left unstimulated or treated with IL-4 (10 ng/ml)/IL-13 (10 ng/ml) for the indicated periods of time. Cell extracts from the resulting cells were subjected to WB analyses.

G Stat6 is recruited on the *Elp3* promoter in an IL-4-dependent manner. ChIP assays using an anti-Stat6 antibody were conducted in RAW 264.7 cells to assess Stat6 recruitment on a Stat-DNA binding site found in the *Elp3* promoter. IgG antibody was used as a negative result. The histogram shows Stat6 recruitment on the Stat DNA-binding site with or without IL-4 (10 ng/ml) treatment for 2 h. Results obtained with four independent experiments (mean ± SD, Student t-test, *P < 0.05) are illustrated.

H–J *Elp3* deficiency in myeloid cells impairs multiple signaling pathways triggered by IL-4 (H, I, and J), IL-13 (H) or by both cytokines (H). Peritoneal macrophages isolated from the indicated mouse genotypes were untreated or stimulated with IL-4 (10 ng/ml) (H, I, and J), IL-13 (10 ng/ml) (H) or with both cytokines (H) for 8 h (H) or for the indicated periods of time (I and J) and the resulting cell extracts were subjected to WB analyses.

K *Elp3* deficiency in BMDMs impairs the production of M2 markers upon stimulation with IL-4/IL-13. mRNAs of M2 markers were assessed by Real Time PCR. mRNA levels in unstimulated macrophages from $Elp3^{Control}$ mice were set to 1 and levels in other experimental conditions were relative to that after normalization with Gapdh mRNA levels (n = 4 mice; mean ± SD, Student t-test, *P < 0.05, ***P < 0.001).

L Elp3 promotes M2 macrophage polarization. Peritoneal macrophages isolated from the indicated mouse genotypes were untreated or stimulated with IL-4/IL-13 (10 ng/ml) for 24 h and the resulting cell extracts were subjected to WB analyses.

Source data are available online for this figure.

 © 2022 The Authors

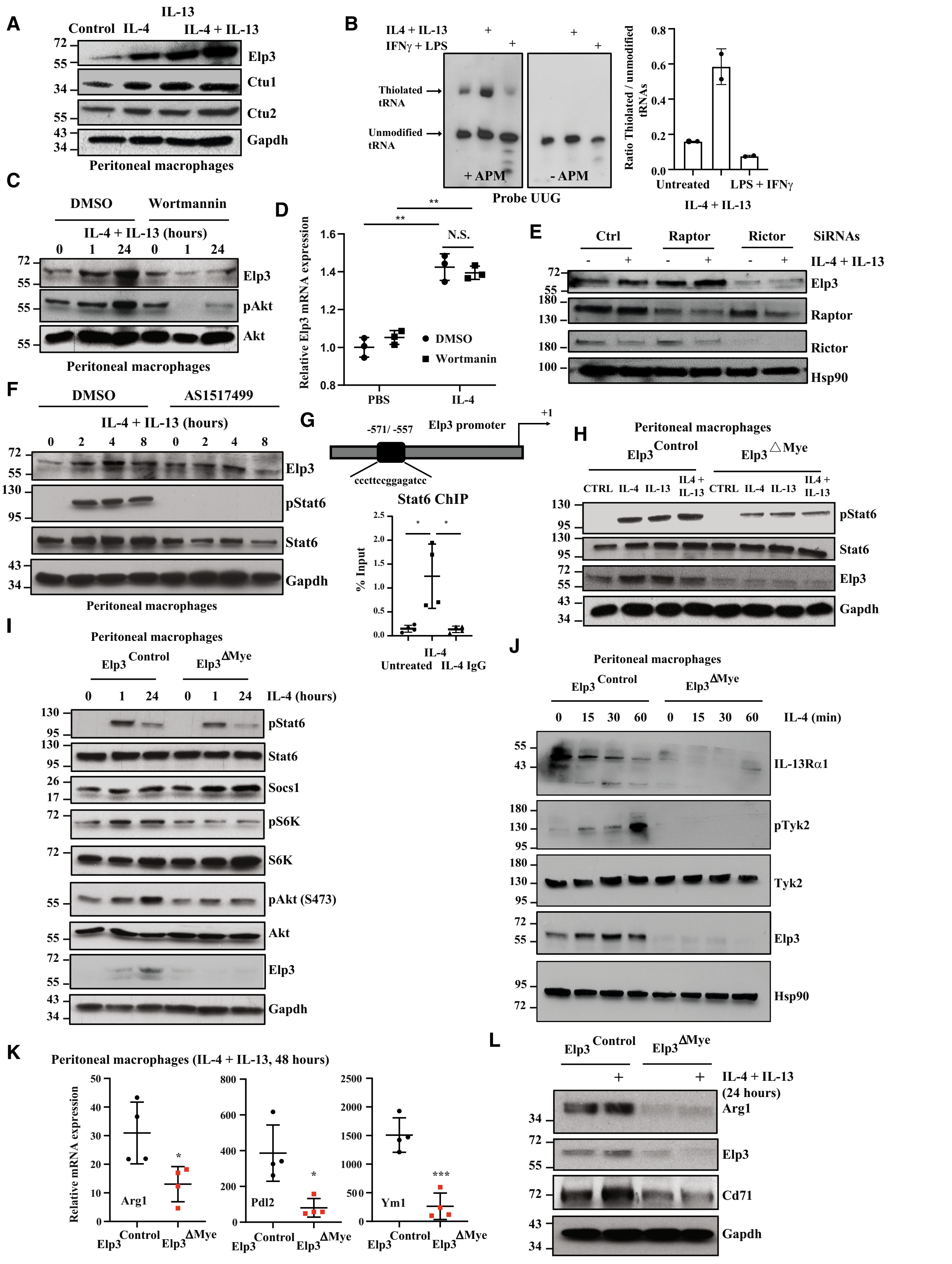
A
Control IL-4 IL-13 IL-4 + IL-13
Elp3
Ctu1
Ctu2
Gapdh
Peritoneal macrophages
B
IL4 + IL-13
IFNγ + LPS
Thiolated tRNA
Unmodified tRNA
+ APM
- APM
Probe UUG
Ratio Thiolated / unmodified tRNAs
Untreated
LPS + IFNγ
IL-4 + IL-13
C
DMSO
Wortmannin
IL-4 + IL-13 (hours)
Elp3
pAkt
Akt
Peritoneal macrophages
D
Relative Elp3 mRNA expression
N.S.
DMSO
Wortmannin
PBS
IL-4
E
Ctrl
Raptor
Rictor
SiRNAs
IL-4 + IL-13
Elp3
Raptor
Rictor
Hsp90
F
DMSO
AS1517499
IL-4 + IL-13 (hours)
Elp3
pStat6
Stat6
Gapdh
Peritoneal macrophages
G
-571/ -557
Elp3 promoter
+1
cccttccggagatcc
Stat6 ChIP
% Input
IL-4
Untreated IL-4 IgG
H
Peritoneal macrophages
Elp3 Control
Elp3 △Mye
CTRL IL-4 IL-13 IL4 + IL-13 CTRL IL-4 IL-13 IL4 + IL-13
pStat6
Stat6
Elp3
Gapdh
I
Peritoneal macrophages
Elp3 Control
Elp3 ΔMye
IL-4 (hours)
pStat6
Stat6
Socs1
pS6K
S6K
pAkt (S473)
Akt
Elp3
Gapdh
J
Peritoneal macrophages
Elp3 Control
Elp3 ΔMye
IL-4 (min)
IL-13Rα1
pTyk2
Tyk2
Elp3
Hsp90
K
Peritoneal macrophages (IL-4 + IL-13, 48 hours)
Relative mRNA expression
Arg1
Pdl2
Ym1
Elp3 Control
Elp3 ΔMye
L
Elp3 Control
Elp3 ΔMye
IL-4 + IL-13 (24 hours)
Arg1
Elp3
Cd71
Gapdh

Figure 3.

also interfered with the induction of Elp3 expression by IL-4 in peritoneal macrophages (Fig 3F). Consistently, Stat6 was also recruited on a Stat DNA-binding site in the *Elp3* promoter in an IL-4-dependent manner (Fig 3G). Therefore, IL-4 relies on both Stat6 and mTORC2 to induce Elp3 expression in macrophages.

Elp3 promotes M2 macrophage polarization

Having established that IL-4/IL-13 induce Elp3 expression, we next assessed whether loss of *Elp3* impairs M2 macrophage polarization. The genetic inactivation of *Elp3* in peritoneal macrophages impaired Stat6 phosphorylation upon stimulation with IL-4, IL-13 or with both cytokines (Fig 3H and I). Of note, protein levels of Socs1, a negative regulator of Stat signaling, were increased upon IL-4 stimulation in peritoneal macrophages from *Elp3*$^{\Delta Mye}$ mice (Fig 3I). Moreover, IL-4-dependent S6K phosphorylation was also defective upon Elp3 deficiency (Fig 3I). Importantly, mTORC2-dependent Akt phosphorylation on serine 473 was also impaired following Elp3 deficiency (Fig 3I). Therefore, *Elp3* deficiency impairs multiple signaling pathways triggered by IL-4 in peritoneal macrophages. To learn more on molecular mechanisms by which Elp3 promotes Stat6 activation upon IL-4 stimulation, we looked at early events occurring in this signaling pathway. Protein levels of IL-13Rα1 were decreased in peritoneal macrophages lacking Elp3 expression (Fig 3J). Moreover, Tyk2, a JAK kinase family member, was not properly phosphorylated upon IL-4 stimulation in peritoneal macrophages lacking Elp3, indicating that very early events in IL-4 signaling are controlled by Elp3 (Fig 3J). As IL-4 signaling is impaired upon *Elp3* deficiency, the expression of M2 markers such as arginase 1, Cd71, Pdl2, and Ym1 was reduced in IL-4/IL-13-stimulated peritoneal macrophages from *Elp3*$^{\Delta Mye}$ mice (Fig 3K and L). Consistently, Ctu2 deficiency in BMDMs also impaired IL-4-dependent Akt phosphorylation on S473 and also impaired the production of M2 markers in IL-4-stimulated BMDMs (Fig EV2B and C, respectively).

To further explore the role of Elp3 in M2 macrophage polarization, we conducted unbiased transcriptomic analyses (RNA-Seq) combined with Gene Set Enrichment analyses (GSEA) using BMDMs from *Elp3*^{Control} and *Elp3*$^{\Delta Mye}$ mice treated or not with IL-4 *ex-vivo* for 2 h. Genes normally induced through LPS-dependent pathways were upregulated upon *Elp3* deficiency, which fits with our data showing that *Elp3* deficiency potentiates M1 macrophage polarization (Fig 4A). Importantly, a Pparγ-dependent signature was lost upon *Elp3* deficiency, which also fits with our data demonstrating that M2 macrophage polarization relies on Elp3 (Fig 4A). Consistently, the induction of Pparγ by IL-4/IL-13 was abolished in peritoneal macrophages lacking Elp3 (Fig 4B). Dozens of mRNAs whose expression is induced by IL-4 and linked to M2 macrophage polarization were actually not properly induced in IL-4-stimulated BMDMs lacking Elp3 (Fig 4C). Interestingly, Irf4, which acts downstream of mTORC2 to promote M2 macrophage polarization (Huang *et al*, 2016), was not properly expressed upon *Elp3* deficiency in peritoneal macrophages (Fig 4D). Besides these top 50 genes whose expression was impaired upon IL-4 treatment in BMDMs lacking Elp3, we also identified 806 less expressed genes as well as 416 genes whose expression increased in IL-4-induced BMDMs from *Elp3*$^{\Delta Mye}$ mice compared to BMDMs from *Elp3*^{Control} mice (Fig 4E). To study the consequences of *Elp3* deficiency on the proteome of macrophages undergoing M2 polarization, we performed an integrated comparative proteomic analysis of *Elp3*^{Control} and *Elp3*$^{\Delta Mye}$ BMDMs treated with IL-4 for 4 h. Correlation analyses of RNA-seq and proteomic data showed that 139 proteins were significantly downregulated in BMDMs from *Elp3*^{Mye} mice, while corresponding mRNAs did not change (Fig 4F). Interestingly, many of these proteins showed a clear prominent mRNA use of codons Lys^{AAA}, Gln^{CAA}, and Glu^{GAA} known to rely on Elp3 to be translated (Fig 4G). Collectively, our data demonstrate that Elp3 is a key enzyme involved in M2 macrophage polarization.

Metabolic reprogramming linked to M2 macrophage polarization requires Elp3

As macrophage polarization is intrinsically linked to metabolic reprogramming, we next investigated whether *Elp3* deficiency had any impact on metabolic remodeling. Extracts from peritoneal macrophages of *Elp3*^{Control} and *Elp3*$^{\Delta Mye}$ mice treated or not with IL-4 *ex-vivo* were subjected to mass spectrometry analyses in order to

Figure 4. The transcriptomic reprogramming during M2 macrophage polarization relies on Elp3.

A *Elp3* deficiency potentiates the expression of genes induced by LPS-dependent pathways and impairs a Pparγ-dependent transcriptional signature. The figure shows some GSEA analyses carried out on RNA-Seq data done with total RNAs of BMDMs from *Elp3*^{Control} and *Elp3*$^{\Delta Mye}$ mice treated with IL-4 *ex-vivo*.

B Defective induction of Pparγ by IL-4/IL-13 upon *Elp3* deficiency. Cell extracts from peritoneal macrophages of *Elp3*^{Control} and *Elp3*$^{\Delta Mye}$ mice treated or not with IL-4/IL-13 were subjected to WB analyses.

C *Elp3* deficiency impairs the IL-4-dependent transcriptional signature. Cell extracts from BMDMs of *Elp3*^{Control} and *Elp3*$^{\Delta Mye}$ mice treated or not with IL-4 for 2 h were subjected to RNA-Seq experiments. A heatmap is illustrated to show the defective induction of 50 genes by IL-4 upon *Elp3* deficiency.

D Defective Irf4 expression upon IL-4 stimulation in peritoneal macrophages lacking Elp3 expression. Irf4 mRNA levels in peritoneal macrophages from untreated *Elp3*^{Control} mice were set to 1 and levels in other experimental conditions were relative to that after normalization with Gapdh mRNA levels (n = 3 mice; mean ± SD, Student t-test, $^{**}P < 0.01$).

E The IL-4-dependent transcriptomic signature requires Elp3. A volcano plot illustrating changes in mRNA levels in IL-4-stimulated BMDMs (2 h) from *Elp3*^{Control} and *Elp3*$^{\Delta Mye}$ mice is shown.

F Identification of candidates whose protein but not mRNA levels are decreased in IL-4-treated BMDMs lacking Elp3. A volcano plot of correlation between changes in protein and mRNA levels is illustrated. Protein were extracted from BMDMs of *Elp3*^{Control} and *Elp3*$^{\Delta Mye}$ mice treated for 4 h with IL-4. Red dots indicate candidates whose protein but not mRNA levels were downregulated in BMDMs from *Elp3*^{Mye} mice compared to BMDMs from *Elp3*^{Control} mice.

G Identification of candidates enriched in Lys^{AAA}, Gln^{CAA}, and Glu^{GAA} codons whose expression decreases in BMDMs lacking Elp3. A heatmap of the top 40 genes enriched in these codons is illustrated.

Source data are available online for this figure.

 © 2022 The Authors

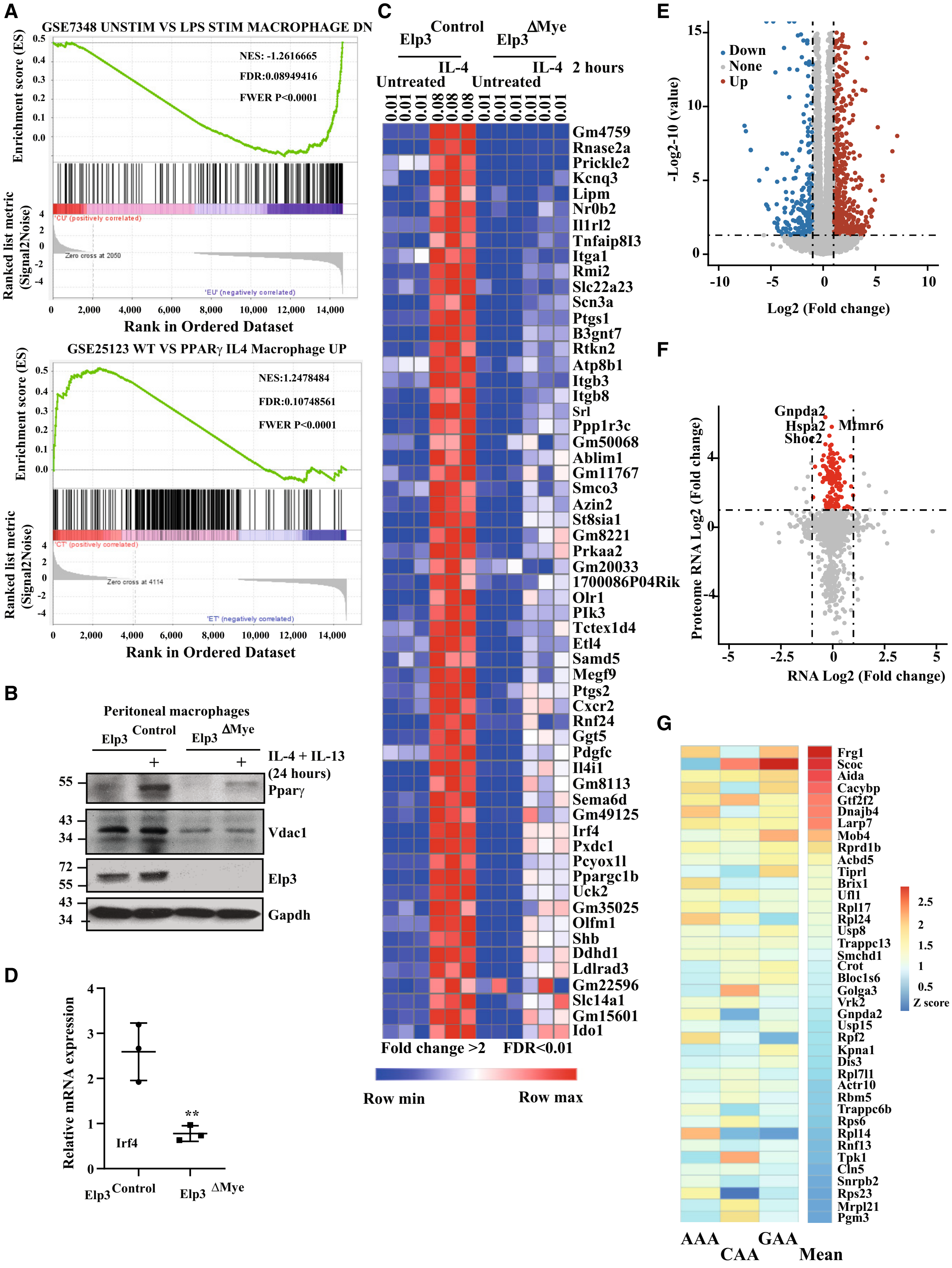

A
GSE7348 UNSTIM VS LPS STIM MACROPHAGE DN
NES: -1.2616665
FDR:0.08949416
FWER P<0.0001
Enrichment score (ES)
Ranked list metric (Signal2Noise)
'CU' (positively correlated)
Zero cross at 2050
'EU' (negatively correlated)
Rank in Ordered Dataset
GSE25123 WT VS PPARγ IL4 Macrophage UP
NES:1.2478484
FDR:0.10748561
FWER P<0.0001
'CT' (positively correlated)
Zero cross at 4114
'ET' (negatively correlated)
Rank in Ordered Dataset
B
Peritoneal macrophages
Elp3 Control
Elp3 ΔMye
IL-4 + IL-13 (24 hours)
Pparγ
Vdac1
Elp3
Gapdh
D
Relative mRNA expression
Irf4
**
Elp3 Control
Elp3 ΔMye
C
Elp3 Control
Elp3 ΔMye
Untreated
IL-4
2 hours
Gm4759
Rnase2a
Prickle2
Kcnq3
Lipm
Nr0b2
Il1rl2
Tnfaip8l3
Itga1
Rmi2
Slc22a23
Scn3a
Ptgs1
B3gnt7
Rtkn2
Atp8b1
Itgb3
Itgb8
Srl
Ppp1r3c
Gm50068
Ablim1
Gm11767
Smco3
Azin2
St8sia1
Gm8221
Prkaa2
Gm20033
1700086P04Rik
Olr1
Plk3
Tctex1d4
Etl4
Samd5
Megf9
Ptgs2
Cxcr2
Rnf24
Ggt5
Pdgfc
Il4i1
Gm8113
Sema6d
Gm49125
Irf4
Pxdc1
Pcyox1l
Ppargc1b
Uck2
Gm35025
Olfm1
Shb
Ddhd1
Ldlrad3
Gm22596
Slc14a1
Gm15601
Ido1
Fold change >2
FDR<0.01
Row min
Row max
E
Down
None
Up
-Log2-10 (value)
Log2 (Fold change)
F
Gnpda2
Hspa2
Mlmr6
Shoc2
Proteome RNA Log2 (Fold change)
RNA Log2 (Fold change)
G
Frg1
Scoc
Aida
Cacybp
Gtf2f2
Dnajb4
Larp7
Mob4
Rprd1b
Acbd5
Tiprl
Brix1
Ufl1
Rpl17
Rpl24
Usp8
Trappc13
Smchd1
Crot
Bloc1s6
Golga3
Vrk2
Gnpda2
Usp15
Rpf2
Kpna1
Dis3
Rpl7l1
Actr10
Rbm5
Trappc6b
Rps6
Rpl14
Rnf13
Tpk1
Cln5
Snrpb2
Rps23
Mrpl21
Pgm3
Z score
AAA
CAA
GAA
Mean

Figure 4.

establish their metabolic profile. In agreement with the fact that M2 macrophages rely on TCA cycle for glucose metabolism and energy generation (O'Neill, 2016) and in agreement with our data showing that *Elp3* deficiency impairs M2 macrophage polarization, TCA metabolites (malate, fumarate) accumulated in IL-4-stimulated peritoneal macrophages lacking Elp3 expression, which reflects a defective TCA cycle (Figs 5A and EV3). This conclusion was raised through an enrichment overview of our metabolomic data (Fig 5B). Glutamine metabolism is another feature of M2 macrophages (Jha *et al*, 2015). In this context, α-ketoglutarate, which is produced via glutaminolysis, is essential for M2 macrophage polarization (Liu *et al*, 2017). Interestingly, α-ketoglutarate levels were decreased in IL-4-stimulated peritoneal macrophages from *Elp3*$^{\Delta Mye}$ mice, suggesting that glutaminolysis is impaired without Elp3 (Fig EV3). Moreover, glucose consumption has been reported to be elevated in M2 macrophages (Huang *et al*, 2016). Consistent with a defective M2 polarization without Elp3, glucose consumption was indeed impaired in macrophages from *Elp3*$^{\Delta Mye}$ mice (Fig 5C). The use of glucose for UDP-GlcNAc synthesis is another metabolic feature of M2 macrophages (Jha *et al*, 2015). UDP-GlcNAc levels were downregulated upon *Elp3* deficiency in IL-4-stimulated peritoneal macrophages (Fig 5A). Therefore, several metabolic pathways involved in M2 macrophage polarization, critically rely on Elp3.

To learn more on molecular mechanisms by which Elp3 controls glucose consumption in macrophages, we looked at expression level of Glut1, a glucose transporter and found that Glut1 mRNA levels were severely decreased in peritoneal macrophages from *Elp3*$^{\Delta Mye}$ mice (Fig 5D). As the rate of conversion of glucose into pyruvate, a key step of glycolysis, accelerates the net rate of glucose consumption, we next looked at expression levels of platelet phosphofructokinase (Pfkp) and noticed a severely decreased expression upon *Elp3* deficiency as were mRNA levels of glucose phosphate isomerase (Gpi1; Fig 5D). Therefore, Elp3 controls glucose consumption in macrophages, at least by promoting the expression of glucose transporter and enzymes involved in glycolysis.

Elp3 controls mitochondrial function in macrophages

A defective TCA cycle observed upon *Elp3* deficiency suggests that some mitochondrial functions may be disrupted. We indeed noticed that peritoneal macrophages lacking Elp3 showed a reduced mitochondrial complex I activity (Fig 5E). We also observed that the mitochondrial membrane potential in peritoneal macrophages from *Elp3*$^{\Delta Mye}$ mice was impaired, as evidenced by FACS using potential-sensitive tetramethylrhodamine methyl ester (TMRM) fluorescence (Fig 5F). We next explored whether Elp3 regulates mitochondrial oxidative phosphorylation based on the oxygen consumption rate (OCR), a measure of electron transport chain activity. Maximal but not basal OCR was significantly decreased in peritoneal macrophages from *Elp3*$^{\Delta Mye}$ mice (Fig 5G). Consistently, the spare OCR capacity was decreased upon *Elp3* deficiency in peritoneal macrophages (Fig 5G). Collectively, our data indicate that Elp3 expression is required for mitochondrial functions in peritoneal macrophages.

Elp3 promotes M2 macrophage polarization *in vivo*

To further assess whether M2 macrophage polarization requires Elp3 *in vivo*, mice were administered with recombinant IL-4 complexed with anti-IL-4 monoclonal antibody (IL-4c) to extend the bioactive half-life of IL-4 *in vivo* and the expression of M2 markers (arginase 1, Ucp1, and Ym1) were quantified. Levels of these M2 markers were severely impaired in *Elp3*$^{\Delta Mye}$ mice (Fig 6A). The i.p injection of IL-4c also elicits the robust proliferation of resident macrophages in the pleural and peritoneal cavity (Jenkins *et al*, 2011). These peritoneal macrophages (PMs) have been classified into two groups, referred to as large peritoneal macrophages (LPMs), which are resident macrophages and small peritoneal macrophages (SPMs), derived from bone marrow, according to their morphology (Cassado *et al*, 2015). Interestingly, loss of *Elp3* impaired IL-4c-induced LPM numbers but did not change the number of SPMs (Fig 6B and C, respectively). To address whether the lack of amplification of LPMs in IL-4c-treated *Elp3*$^{\Delta Mye}$ mice was due to impaired proliferation, we stained peritoneal cells from both *Elp3*^{Control} and *Elp3*$^{\Delta Mye}$ mice for Ki67 and EdU, two markers of cycling cells. *Elp3* deficiency decreased the number of both Ki67^{+} LPMs and EdU^{+} PMs (Fig 6D and E, respectively). To further characterize this cell proliferation defect in macrophages lacking Elp3, GSEA analyses for Hallmark gene sets were conducted and showed prominent enrichment of gene sets related to proliferation, including the mitotic spindle, G2/M checkpoint, and E2F targets gene sets, in our RNA-Seq data from IL-4 treated *Elp3*^{Control} and *Elp3*$^{\Delta Mye}$ BMDMs

Figure 5. The transcriptomic reprogramming during M2 macrophage polarization relies on Elp3. ▶

A *Elp3* deficiency impairs levels of TCA cycle metabolites in IL-4-stimulated peritoneal macrophages. Peritoneal macrophages from *Elp3*^{Control} and *Elp3*$^{\Delta Mye}$ mice (n = 3 per genotype) were treated with IL-4 for 24 h *ex-vivo* and the resulting extracts were subjected to Mass Spec analyses to extensively quantify metabolites.

B *Elp3* deficiency is linked to a defective TCA cycle in peritoneal macrophages. Enrichment analyses carried out on our metabolomics data are illustrated.

C Elp3 controls glucose consumption in peritoneal macrophages. The glucose consumption rate was measured in peritoneal macrophages from *Elp3*^{Control} and *Elp3*$^{\Delta Mye}$ mice (see Methods for details; n = 3 mice per genotype; mean ± SD, Student t-test, *P < 0.05).

D *Elp3* deficiency in peritoneal macrophages impairs the expression of enzymes involved in glucose consumption. mRNA levels of the indicated candidates in peritoneal macrophages from *Elp3*^{Control} mice were set to 1 and levels in *Elp3*$^{\Delta Mye}$ mice were relative to that after normalization with Gapdh mRNA levels (n = 3 mice per genotype; mean ± SD, Student t-test, ***P < 0.001, **P < 0.01, *P < 0.05).

E, F *Elp3* deficiency in peritoneal macrophages impairs mitochondrial complex I activity (E) and the mitochondrial membrane potential (F). Extracts from peritoneal macrophages of *Elp3*^{Control} and *Elp3*$^{\Delta Mye}$ mice were used to assess complex I activity (E) (n = 3 mice per genotype; mean ± SD, Student t-test, *P < 0.05). The mitochondrial membrane potential was assessed by FACS analyses by following-up TMRM (n = 3 mice per genotype; mean ± SD, Student t-test, ***P < 0.001). Representative FACS panels are illustrated on the right.

G Elp3 expression in peritoneal macrophages is required for mitochondrial oxidative phosphorylation. Both basal and maximal oxygen consumption rate (OCR) as well as the spare OCR were measured with extracts from peritoneal macrophages of both *Elp3*^{Control} and *Elp3*$^{\Delta Mye}$ mice (see Methods for details; n = 3 mice per genotype; mean ± SD, Student t-test, ***P < 0.001, **P < 0.01).

 © 2022 The Authors

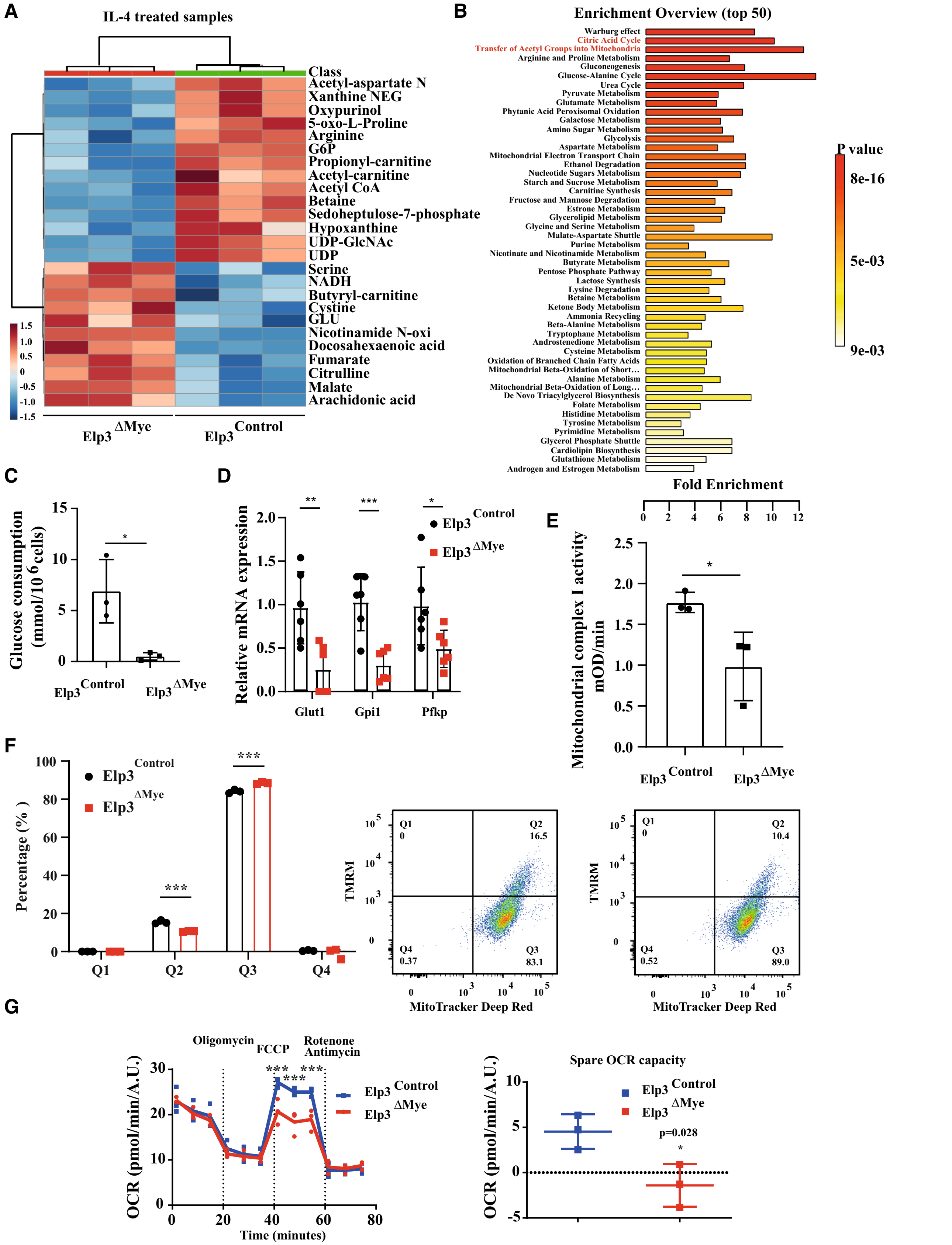
A
IL-4 treated samples
Class
Acetyl-aspartate N
Xanthine NEG
Oxypurinol
5-oxo-L-Proline
Arginine
G6P
Propionyl-carnitine
Acetyl-carnitine
Acetyl CoA
Betaine
Sedoheptulose-7-phosphate
Hypoxanthine
UDP-GlcNAc
UDP
Serine
NADH
Butyryl-carnitine
Cystine
GLU
Nicotinamide N-oxi
Docosahexaenoic acid
Fumarate
Citrulline
Malate
Arachidonic acid
Elp3 ΔMye
Elp3 Control
B
Enrichment Overview (top 50)
Warburg effect
Citric Acid Cycle
Transfer of Acetyl Groups into Mitochondria
Arginine and Proline Metabolism
Gluconeogenesis
Glucose-Alanine Cycle
Urea Cycle
Pyruvate Metabolism
Glutamate Metabolism
Phytanic Acid Peroxisomal Oxidation
Galactose Metabolism
Amino Sugar Metabolism
Glycolysis
Aspartate Metabolism
Mitochondrial Electron Transport Chain
Ethanol Degradation
Nucleotide Sugars Metabolism
Starch and Sucrose Metabolism
Carnitine Synthesis
Fructose and Mannose Degradation
Estrone Metabolism
Glycerolipid Metabolism
Glycine and Serine Metabolism
Malate-Aspartate Shuttle
Purine Metabolism
Nicotinate and Nicotinamide Metabolism
Butyrate Metabolism
Pentose Phosphate Pathway
Lactose Synthesis
Lysine Degradation
Betaine Metabolism
Ketone Body Metabolism
Ammonia Recycling
Beta-Alanine Metabolism
Tryptophane Metabolism
Androstenedione Metabolism
Cysteine Metabolism
Oxidation of Branched Chain Fatty Acids
Mitochondrial Beta-Oxidation of Short…
Alanine Metabolism
Mitochondrial Beta-Oxidation of Long…
De Novo Triacylglycerol Biosynthesis
Folate Metabolism
Histidine Metabolism
Tyrosine Metabolism
Pyrimidine Metabolism
Glycerol Phosphate Shuttle
Cardiolipin Biosynthesis
Glutathione Metabolism
Androgen and Estrogen Metabolism
P value
8e-16
5e-03
9e-03
Fold Enrichment
C
Glucose consumption (mmol/10^6 cells)
Elp3 Control
Elp3 ΔMye
D
Relative mRNA expression
Elp3 Control
Elp3 ΔMye
Glut1
Gpi1
Pfkp
E
Mitochondrial complex I activity mOD/min
Elp3 Control
Elp3 ΔMye
F
Percentage (%)
Elp3 Control
Elp3 ΔMye
Q1
Q2
Q3
Q4
TMRM
MitoTracker Deep Red
Q1 0
Q2 16.5
Q4 0.37
Q3 83.1
Q1 0
Q2 10.4
Q4 0.52
Q3 89.0
G
Oligomycin
FCCP
Rotenone Antimycin
OCR (pmol/min/A.U.)
Time (minutes)
Elp3 Control
Elp3 ΔMye
Spare OCR capacity
p=0.028

Figure 5.

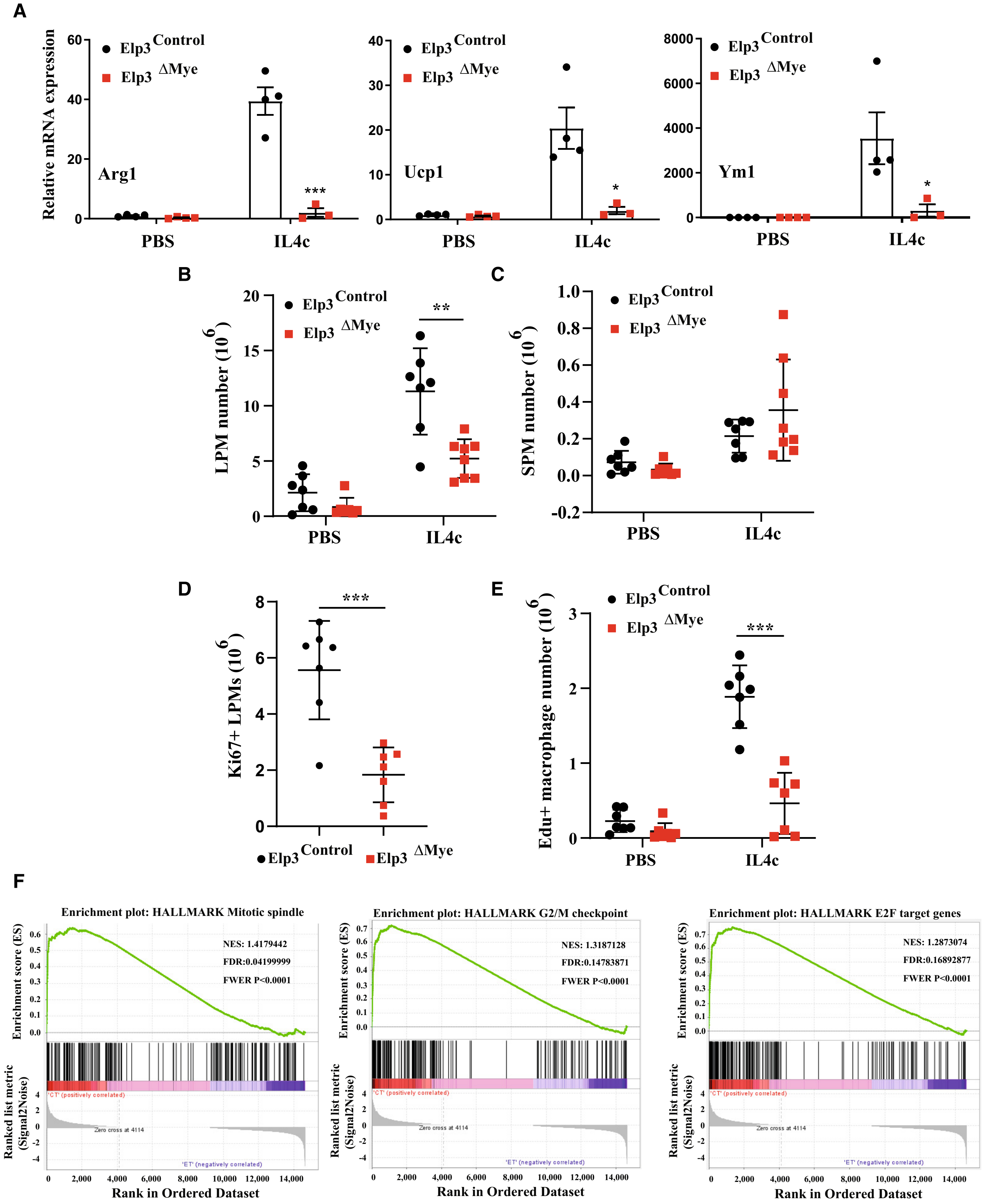

Figure 6.

 © 2022 The Authors

Figure 6. Elp3 promotes M2 macrophage polarization *in vivo*.

A Impaired production of M2 markers upon IL-4 activation *in vivo* in mice lacking *Elp3* in peritoneal macrophages. Mice of the indicated genotypes were treated with PBS or stimulated with IL-4 complex (IL-4c) and mRNA levels of the indicated candidates were assessed by Real Time PCR in the peritoneal exudate cells 4 days after the stimulation. mRNA levels in peritoneal exudate cells from untreated $Elp3^{Control}$ mice were set to 1 and levels in other experimental conditions were relative to that after normalization with Gapdh mRNA levels ($n = 4$ mice; mean ± SD, Student *t*-test, $^{***}P < 0.001$, $^{**}P < 0.01$, $^{*}P < 0.05$).

B Decreased number of large peritoneal macrophages (LPMs) lacking Elp3 during M2 polarization. Numbers of LPMs ($CD115^+$, $ICAM2^+$, $MHCII^{low}$) sorted from $Elp3^{Control}$ and $Elp3^{\Delta Mye}$ mice treated by IL-4c are plotted (representative of two experiments, $n = 7$ mice per group, mean ± SD, Student *t*-test, $^{**}P < 0.01$).

C *Elp3* deficiency does not impact cell proliferation of small peritoneal macrophages upon IL-4c stimulation. Numbers of SPMs ($CD115^+$, $ICAM2^+$, MHC II^{high}) sorted from $Elp3^{Control}$ and $Elp3^{\Delta Mye}$ mice treated by IL-4c are plotted and data are illustrated as in B. (representative of two experiments, $n = 7$ per group, mean ± SD, Student *t*-test, nonsignificant).

D Decreased number of cycling LPMs lacking Elp3 upon stimulation with IL-4c. Numbers of $Ki67^+$ LPMs sorted from $Elp3^{Control}$ and $Elp3^{\Delta Mye}$ mice treated by IL-4c are plotted (representative of two experiments, $n = 7$ mice per group, mean ± SD, Student *t*-test, $^{***}P < 0.001$).

E Decreased number of Edu^+ PM cells lacking Elp3 upon stimulation with IL-4c. Numbers of Edu^+ $CD11^+$ cells sorted from $Elp3^{Control}$ and $Elp3^{\Delta Mye}$ mice treated by IL-4c are plotted (representative of two experiments, $n = 7$ per group, mean ± SD, Student *t*-test, $^{***}P < 0.001$).

F Elp3 controls the proliferation of macrophages undergoing M2 polarization. GSEA analyses with gene expression RNA-seq data are illustrated. Representative Hallmarks enriched in $Elp3^{Control}$ versus $Elp3^{\Delta Mye}$ BMDMs treated by IL-4 for 2 h are shown.

(Fig 6F). Therefore, Elp3 promotes M2 macrophage polarization *in vivo*, at least by supporting macrophage proliferation upon stimulation with IL-4c.

Elp3 promotes Ric8b expression in myeloid cells

As Elp3 and mTORC2 deficiencies share overlapping phenotypes (Hallowell *et al*, 2017), we next looked for any positive regulator of mTORC2 activation whose translation may require Elp3. In this context, we got interested in Ric8b, a noncanonical guanine nucleotide exchange factor (GEF) whose deficiency impairs Akt phosphorylation on Serine 473 (Nagai *et al*, 2020). Ric8b mRNA levels were induced by IL-4 in BMDMs from both $Elp3^{Control}$ and $Elp3^{\Delta Mye}$ mice, suggesting that Elp3 does not control Ric8b transcription (Fig EV4A). Importantly, Ric8b protein levels were also increased by IL-4 in BMDMs from $Elp3^{Control}$ but not from $Elp3^{\Delta Mye}$ mice, suggesting that Elp3 may control Ric8b mRNA translation (Fig 7A). This conclusion was further supported by the fact that the induction of Elp3 and Ric8b expression by IL-4 followed similar kinetics (Fig 7A). To explore whether Elp3 promotes Ric8b mRNA translation, we carried out a Puro-PLA assay to detect newly synthesized Ric8b proteins in IL-4-stimulated BMDMs from both $Elp3^{Control}$ and $Elp3^{\Delta Mye}$ mice. Interestingly, *Elp3* deficiency interfered with the production of Ric8b proteins, suggesting that Ric8b is one candidate whose mRNA translation is regulated by Elp3-dependent tRNA modifications (Fig 7B). As Elp3 controls mRNA translation in a codon-dependent manner (Bauer *et al*, 2012), we generated a Ric8b mutant in which Lys^{AAA}, Gln^{CAA}, and Glu^{GAA} codons known to rely on Elp3 to be properly decoded ("Ric8b WT") where replaced by their cognate synonymous Lys^{AAG}, Gln^{CAG}, and Glu^{GAG} codons which do not require the U34 mcm^5s^2 for their translation ("Ric8b MUT"; Fig 7C). While *Elp3* deficiency in BMDMs interfered with the production of Ric8b WT, Ric8b MUT was properly expressed in macrophages lacking Elp3 (Fig 7C). Collectively, our data define Ric8b as a direct target of Elp3 in macrophages. We next depleted Ric8b in BMDMs and assessed the consequences on IL-4-dependent mTORC2 activation. Ric8b-depleted BMDMs did not properly phosphorylate Akt and Ndrg1 upon IL-4 stimulation, indicating that mTORC2 activation indeed relies on Ric8b (Fig 7D and E, respectively). Of note, Ric8b deficiency also impaired mTORC2 activation by LPS/IFNγ, as judged by a defective Akt and Ndrg1 phosphorylation (Fig EV4B). As IL-4-dependent signaling was defective upon Ric8b deficiency, the production of M2 markers was also impaired in BMDMs lacking Ric8b upon stimulation with IL-4/IL-13 (Fig 7F and G). Similarly to *Elp3*

Figure 7. Elp3 promotes Ric8b expression.

A The induction of Ric8b expression by IL-4 relies on Elp3. BMDMs from the indicated genotypes were treated or not with IL-4 (10 ng/ml) up to 24 h and the resulting cell extracts were subjected to WB analyses.

B Elp3 promotes Ric8B mRNA translation. BMDMs from $Elp3^{Control}$ and $Elp3^{\Delta Mye}$ mice were treated by IL-4 for 24 h, followed by a treatment with 10 μg/ml puromycin for 5 min. To detect newly synthesized Ric8b proteins, a Puro-PLA assay was conducted. Representative images detecting Ric8b (Red) and α-$Tubulin^+$ microtubules (green) in BMDMs are illustrated. On the right, the graph shows a quantification of signals for Ric8b in α-$Tubulin^+$ areas. A random of six different areas were counted (6 technical replicates, mean ± SD, Student's *t*-test, $^{*}P < 0.05$).

C Ric8b is a direct target of Elp3. BMDMs from $Elp3^{Control}$ and $Elp3^{\Delta Mye}$ mice were infected with a lentiviral GFP construct (negative control), Flag-Ric8b WT or with Flag-Ricb MUT, as indicated and the resulting protein extracts were subjected to WB analyses to assess Ric8b and Elp3 protein levels. Gapdh was used as a loading control.

D, E Ric8b promotes IL-4-dependent mTORC2 activation. Control or Ric8b-depleted BMDMs were treated or not with IL-4 (10 ng/ml) for the indicated periods of time and the resulting cell extracts were subjected to WB analyses.

F, G Ric8b deficiency impairs the production of M2 markers upon IL-4/IL-13 stimulation. Control or Ric8b-depleted BMDMs were stimulated or not with IL-4/IL-13 (10 ng/ml) for 24 h and the resulting RNAs were subjected to Real-Time PCR analyses. mRNA levels of the indicated candidates in control and unstimulated BMDMs were set to 1 and levels in other experimental conditions were relative to that after normalization with Gapdh mRNA levels ($n = 3$ mice per genotype; mean ± SD, Student *t*-test, $^{***}P < 0.001$, $^{**}P < 0.01$).

H Ric8b controls glucose consumption in BMDMs. The glucose consumption rate was measured in BMDMs transfected with a siRNA CTRL or Ric8b. Data from three biological replicates (3 mice per group) are shown (mean ± SD, Student *t*-test, $^{**}P < 0.01$).

Source data are available online for this figure.

© 2022 The Authors

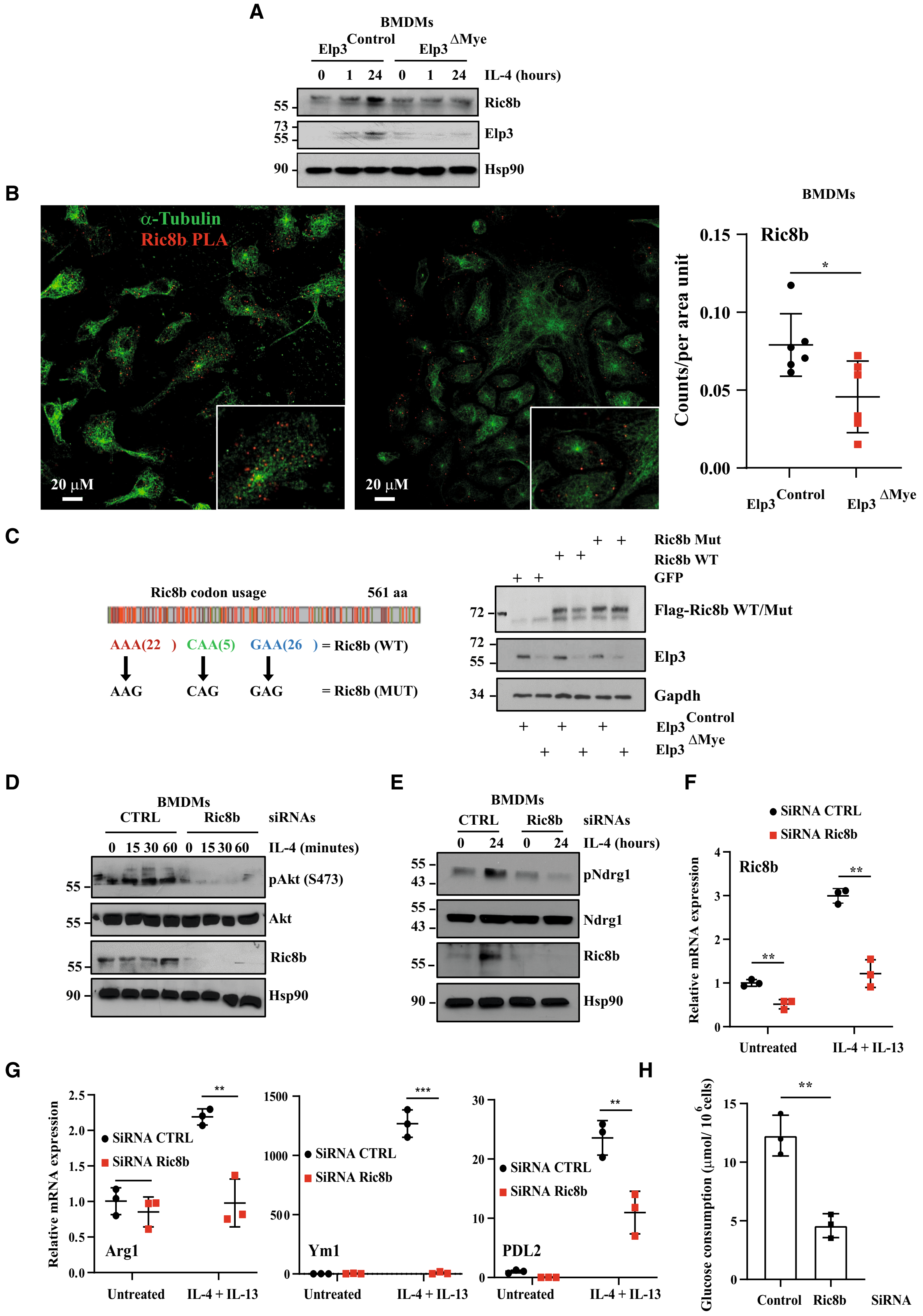

Figure 7.

© 2022 The Authors

deficiency, glucose consumption was also defective in IL-4/IL-13-stimulated BMDMs lacking Ric8b (Fig 7H). Collectively, these data indicate that Elp3 promotes mTORC2 signaling, at least through Ric8b in BMDMs.

Elp3 promotes the expression of some mitochondrial ribosome large subunit proteins

Having demonstrated that Elp3 is critical for mitochondrial functions in metabolic reprogramming linked to macrophage polarization, we next explored the underlying molecular mechanisms. *Elp3* deficiency leads to the accumulation of multiple proteins in aggregates, both in yeast and in mammalian cells (Nedialkova & Leidel, 2015; Rapino *et al*, 2018; Tavares *et al*, 2020). Likewise, *Elp3* deficiency caused the accumulation of protein candidates in aggregates from IL-4-stimulated BMDMs (Fig EV4C). We next isolated aggregates of BMDMs from both *Elp3*^{Control} and *Elp3*$^{\Delta Mye}$ mice and subjected them to electrophoresis followed by Mass Spectrometry analysis (Fig EV4D). One thousand eight hundred and eight proteins were more expressed in aggregates upon IL-4 stimulation and levels of 623 candidates were more elevated upon *Elp3* deficiency in IL-4-stimulated BMDMs (Fig EV4E and F). Therefore, we focused our attention on 206 candidates whose expression was both induced by IL-4 and elevated upon *Elp3* deficiency in aggregates. We first subjected this proteomic signature to a Reactome analysis and highlighted a link with mitochondrial translation (Fig 8A). Interestingly, this signature included several mitochondrial ribosome large subunit proteins (Mrpl) showing an enrichment in Lys^{AAA}, Gln^{CAA}, and Glu^{GAA} codons known to rely on Elp3 to be decoded (Bauer *et al*, 2012; Fig 8B). We conducted western blot analysis using total extracts and found that both Mrpl3 and Mrpl13 levels were induced by IL-4/IL-13 in BMDMs (Fig 8C). Interestingly, protein levels of these mitochondrial candidates were similarly decreased upon IL-4/IL-13 stimulation in both BMDMs and PMs lacking Elp3 expression (Figs 8C and EV4G). Elp3 controls the expression of Mrpl3, Mrpl13, and Mrpl47 at a post-transcriptional level as mRNA levels of these candidates were similar in BMDMs from both *Elp3*^{Control} and *Elp3*$^{\Delta Mye}$ mice (Fig EV4H). These results suggest that mitoribosomes may not be fully functional upon *Elp3* deficiency, with important consequences for mitochondrial translation. In agreement with this hypothesis, we found that protein levels of Mitochondrially Encoded Cytochrome C Oxidase III (Mt-co3) were severely decreased in both BMDMs and PMs lacking Elp3 (Figs 8C and EVI, respectively). Importantly, levels of Succinate dehydrogenase complex, subunit A (Sdha), a mitochondrial enzyme produced from nuclear DNA, were not defective in macrophages lacking Elp3 (Fig 8C). Therefore, *Elp3* deficiency causes defects in mitochondrial translation, at least by impairing levels and consequently functions of mitochondrial ribosomes. Importantly, Elp3 promotes M2 polarization, at least through the production of Mrpl13 as Mrpl13 deficiency in BMDMs also impaired the production of M2 markers upon stimulation with IL-4 (Fig 8D). Collectively, our data demonstrate that the production of some mitochondrial candidates is one mechanism by which Elp3 promotes M2 macrophage polarization.

Lack of Elp3 in peritoneal macrophages does not stabilize Atf4

Elp3 deficiency in T cells or in the hematopoietic system causes Atf4 stabilization (Lemaitre *et al*, 2021; Rosu *et al*, 2021). Although stabilized ATF4 promotes rather than inhibit M2 macrophage polarization (Kim *et al*, 2018), we nevertheless addressed ATF4 expression in peritoneal macrophages lacking Elp3 expression. In contrast to T cells, Atf4 was not stabilized upon *Elp3* deficiency (Fig EV5A). Instead, Atf4 protein levels were decreased, especially after IL-4/IL-13 treatment (Fig EV5A). Consistently, mRNA levels of Atf4 target genes were not increased in IL-4-stimulated peritoneal macrophages lacking Elp3 expression (Fig EV5B). Collectively, these results indicate that *Elp3* deficiency does not stabilize Atf4 in peritoneal macrophages.

Elp3 expression in macrophages promotes Wnt-driven tumor development in the intestine

Intestinal tumors are infiltrated by TAMs, a class of macrophages with M2 features that positively regulates tumor development (Galdiero *et al*, 2013). As Elp3 controls M2 macrophage polarization, we next assessed whether *Elp3* deficiency in myeloid cells had any consequences on Wnt-driven tumor development in the intestine. To achieve this goal, we crossed *Elp3*^{Control} and *Elp3*$^{\Delta Mye}$ mice with the *Apc*$^{+/Min}$ strain which spontaneously develop intestinal adenomas due to constitutive Wnt signaling (Su *et al*, 1992). The genetic inactivation of *Elp3* in myeloid cells extended mice survival in *Apc*$^{+/Min}$ mice (Fig 9A). *Apc*$^{+/Min}$ *Elp3*$^{\Delta Mye}$ mice showed less adenomas in proximal, middle, and distal parts of the intestine than *Apc*$^{+/Min}$ *Elp3*^{Control} mice (Fig 9B). Moreover, splenomegaly, a typical feature observed in *Apc*$^{+/Min}$ mice, was less pronounced in *Apc*$^{+/Min}$ *Elp3*$^{\Delta Mye}$ mice (Fig 9C). Importantly, the number of TAMs, defined as CD301^{+}/F4/80^{+} cells among F4/80^{+} cells was decreased in *Apc*$^{+/Min}$ *Elp3*$^{\Delta Mye}$ mice (Fig 9D). Therefore, *Elp3* deficiency in myeloid cells delays tumor development upon constitutive Wnt signaling, at least by negatively impacting on the pool of TAMs.

Discussion

We defined here tRNA-modifying enzymes Elp3 and Ctu2 as candidates that limit M1 and promote M2 macrophage polarization. We also demonstrate that Elp3 expression in myeloid cells is required in Wnt-driven tumor development in the intestine, at least by maintaining the pool of infiltrated M2-like macrophages in intestinal tumors. Mechanistically, we defined Ric8b and multiple IL-4-induced candidates including some mitochondrial proteins, as candidates whose production relies on Elp3. Therefore, our data indicate that U_{34} tRNA-modifications critically regulate the biology of macrophages.

Macrophage polarization involves a dynamic regulation in the expression of tRNA-modifying enzymes such as Elp1/3 and Ctu1/2. Indeed, Elp3 expression is induced by IL-4 in macrophages but repressed by IFNγ. As a result, the pool of U_{34} chemically modified tRNAs similarly change during macrophage polarization in order to control the translational reprogramming occurring during this process. We showed that Stat6 is recruited on the *Elp3* promoter to drive its expression upon stimulation by IL-4. Moreover, Rictor also promotes Elp3 induction by IL-4, possibly through the mTORC2-dependent phosphorylation of transcription factors recruited on the *Elp3* promoter. Therefore, Elp3 expression is regulated by multiple signaling pathways triggered by IL-4 signaling, as previously

© 2022 The Authors

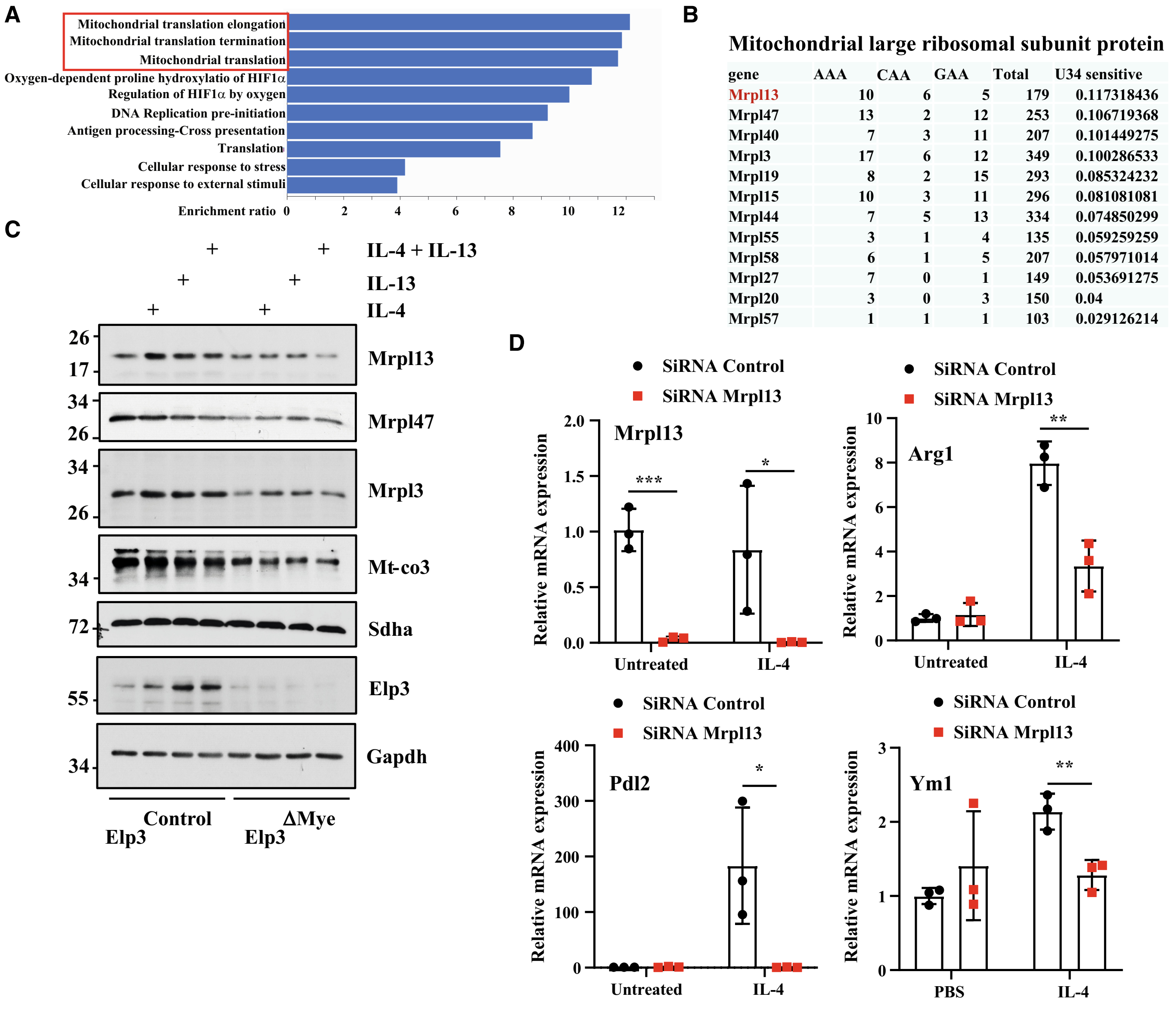

gene	AAA	CAA	GAA	Total	U34 sensitive
Mrpl13	10	6	5	179	0.117318436
Mrpl47	13	2	12	253	0.106719368
Mrpl40	7	3	11	207	0.101449275
Mrpl3	17	6	12	349	0.100286533
Mrpl19	8	2	15	293	0.085324232
Mrpl15	10	3	11	296	0.081081081
Mrpl44	7	5	13	334	0.074850299
Mrpl55	3	1	4	135	0.059259259
Mrpl58	6	1	5	207	0.057971014
Mrpl27	7	0	1	149	0.053691275
Mrpl20	3	0	3	150	0.04
Mrpl57	1	1	1	103	0.029126214

Figure 8. Elp3 controls the expression of some mitochondrial proteins.

A Enrichment of candidates involved in mitochondrial translation in the proteomic signature of 206 candidates (Reactome pathway enrichment analysis).

B Enrichment of Mrpl proteins in the proteomic signature of 206 candidates. Proteins were classified according to the combined frequency of AAA, GAA, and CAA codons in their open reading frame.

C Elp3 promotes mitochondrial translation. BMDMs from the indicated genotypes were treated or not with IL-4/IL-13 (10 ng/ml) for the indicated hours and the resulting cell extracts were subjected to WB analyses.

D Impaired expression of M2 markers upon Mrpl13 deficiency in IL-4-stimulated macrophages. BMDMs were transfected with a control siRNA or with a siRNA targeting Mrpl13 and the resulting cells were untreated or stimulated with IL-4 (10 ng/ml) for 24 h. mRNA levels of the indicated candidates were quantified by Real-Time PCR experiments. mRNA levels in unstimulated BMDMs transfected with the control siRNA were set to 1 and levels in other experimental conditions were related to that. Experiments were conducted in triplicates (mean ± SD, Student *t*-test, ***$P < 0.001$, **$P < 0.01$, *$P < 0.05$).

Source data are available online for this figure.

reported for Irf4 (Huang *et al*, 2016). Interfering with Elp3 or Ctu2 expression has dramatic consequences on cascades induced by polarizing signals and consequently on downstream expression of M1 or M2 markers. Likewise, metabolic reprogramming linked to macrophage polarization is also dramatically deregulated upon *Elp3* deficiency. Although Ctu2 expression is not changed upon stimulation by M1 polarizing signals, Ctu2 deficiency impairs Ctu1 function and consequently the thiolation of some U_{34} tRNAs. As a result, Ctu2 and Elp3 deficiencies share common consequences on macrophage polarization.

 © 2022 The Authors

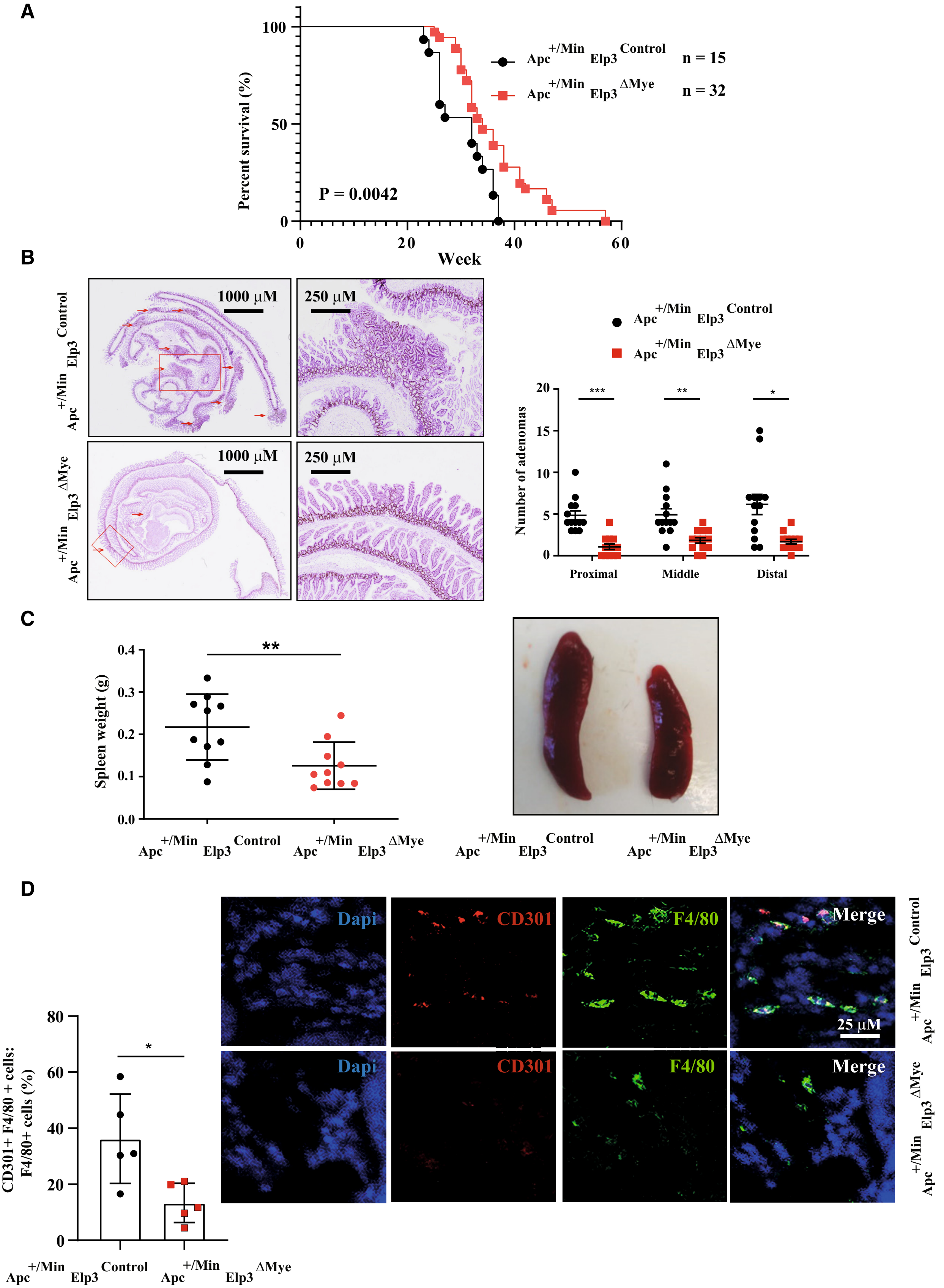

A
Percent survival (%)
100
50
0
0
20
40
60
Week
P = 0.0042
Apc+/Min Elp3Control n = 15
Apc+/Min Elp3ΔMye n = 32
B
Apc+/Min Elp3Control
Apc+/Min Elp3ΔMye
1000 μM
250 μM
Number of adenomas
20
15
10
5
0
***
**
*
Proximal
Middle
Distal
C
Spleen weight (g)
0.4
0.3
0.2
0.1
0.0
**
Apc+/Min Elp3Control
Apc+/Min Elp3ΔMye
D
CD301+ F4/80 + cells: F4/80+ cells (%)
80
60
40
20
0
*
Dapi
CD301
F4/80
Merge
25 μM

Figure 9.

Figure 9. Elp3 expression in myeloid cells promotes Wnt-driven tumor initiation in the intestine.

A, B *Elp3* deficiency in myeloid cells prolongs survival of $Apc^{+/Min}$ mice (A) and reduces the number of adenomas in all parts of the intestine (B). A Kaplan–Meyer curve was established with the indicated genotypes (A). The number of intestinal tumors in the indicated parts of the intestine was quantified in both $Apc^{+/Min}$ $Elp3^{Control}$ and $Apc^{+/Min}$ $Elp3^{\Delta Mye}$ mice (B) (n = 10 mice per genotype; mean ± SD, Student t-test, ***P < 0.001, **P < 0.01, *P < 0.05). Representative H&E analyses of intestinal crypts from the indicated genotypes are illustrated.

C *Elp3* deficiency in myeloid cells decreases the spleen weight of $Apc^{+/Min}$ mice (n = 10 mice per genotype; mean ± SD, Student t-test, **P < 0.01). Representative pictures of the spleen from the indicated genotypes.

D Elp3 expression in myeloid cells maintains the pool of tumor-associated macrophages (TAMs). The number of TAMs, defined as $CD301^+$/$F4/80^+$ cells among $F4/80^+$ cells was quantified in intestinal crypts from both $Apc^{+/Min}$ $Elp3^{Control}$ and $Apc^{+/Min}$ $Elp3^{\Delta Mye}$ mice (n = 5 mice per genotype; mean ± SD, Student t-test, *P < 0.05). Representative immunofluorescence analyses are illustrated.

Source data are available online for this figure.

The phenotype that results from *Elp3* deficiency in macrophages undergoing M1 or M2 polarization resembles what has been reported without mTORC2 activation. Indeed, Akt phosphorylation on serine 473, a residue targeted by mTORC2 upon LPS/IFNγ stimulation, is impaired without Elp3. Moreover, both *Elp3* and *Rictor* deficiencies show elevated levels of cytokines linked to M1 macrophage polarization (Festuccia *et al*, 2014). Likewise, the phosphorylation of Akt by mTORC2, the transcriptional signature and metabolic reprogramming by IL-4 are impaired without Elp3, as previously reported without mTORC2 (Huang *et al*, 2016). Collectively, these data define a biological context in which Elp3 promotes mTORC2 signaling, as demonstrated in yeast (Candiracci *et al*, 2019). As Ctu2, a tRNA-modifying enzyme acting in the same cascade as Elp3, also promotes mTORC2 activation, it is likely that these enzymes are required for the translation of mRNA candidates acting as activators of mTORC2. In this context, we identified Ric8b, a candidate known to promote mTORC2 activation (Nagai *et al*, 2020), as a direct target of Elp3. Ric8b expression is induced by IL-4 in macrophages, similarly to Elp3. Moreover, *Elp3* deficiency interferes with Ric8b expression in IL-4-stimulated macrophages. Ric8b is enriched in codons (9.45%) known to rely on Elp3 to be properly translated. Our data based on experiments conducted with a Ric8b mutant lacking all Lys^{AAA}, Gln^{CAA}, and Glu^{GAA} codons demonstrate that Elp3 promotes Ric8b translation in a codon-dependent manner. Alternatively, Elp3 may have stabilized Ric8b through mTORC2. Such hypothesis indeed explains how Elp3 stabilizes the anti-apoptotic protein Mcl-1 in triple negative breast cancers (Cruz-Gordillo *et al*, 2020). The PI3K/mTOR pathway indeed stabilizes candidates such as Mcl-1 (Wang *et al*, 1999). Therefore, Elp3 may also regulate protein stability of a variety of candidates downstream of mTORC2, in addition to promoting mRNA translation in a codon-dependent manner.

Elp3 may also promote mTORC2 activation through additional, Ric8b-independent pathways. Indeed, a careful analysis of candidates whose protein but not mRNA levels are impaired in IL-4-stimulated BMDMs lacking Elp3 revealed Rps6 whose deficiency attenuates insulin-induced mTORC2 activation (Yano *et al*, 2014).

Beside a role of Elp3 in mTORC2 activation, Elp3 also regulates early steps of IL-4 signaling. Indeed, both Tyk2 and Stat6 phosphorylation triggered by IL-4, which occur through a mTORC2-independent pathway, are defective upon *Elp3* deficiency. One explanation could be the decreased IL-13Rα1 expression in Elp3-depleted peritoneal macrophages. Alternatively, elevated Socs1 levels seen without Elp3 could also underlie defective Stat6 phosphorylation, a mechanism also reported upon *Rictor* deficiency (Hallowell *et al*, 2017). It remains to be demonstrated how Elp3 regulates both Socs1 and IL-13Rα1 expression though.

Elp3 deficiency leads to protein misfolding and aggregation in both yeast and human (Nedialkova & Leidel, 2015; Rapino *et al*, 2018). Interestingly, candidates found in these aggregates are enriched in codons decoded by tRNAs lacking $mcm^5s^2U_{34}$ modifications in Elp3-deficient yeast (Tavares *et al*, 2020). Therefore, it is tempting to speculate that defects in macrophage polarization seen upon *Elp3* deficiency may result, at least in part, from misfolding of a variety of proteins acting downstream of both LPS/IFNγ and IL-4 signaling cascades. In support with this hypothesis, we found that several mitochondrial proteins of ribosome large subunit such as Mrpl3, Mrpl13, and Mrpl 47 accumulate in aggregates of macrophages stimulated with IL-4 upon *Elp3* deficiency. As a result, mitochondrial translation is impaired, as evidenced by decreased protein levels of candidates such as Mt-co3. This finding has important consequences as the depletion of Mrpl13 also leads to defects in M2 macrophage polarization, similarly to *Elp3* deficiency. Therefore, our results demonstrate how critical mitochondrial translation regulated by a tRNA-modifying enzyme is in macrophage polarization.

The loss of *Elp3* in cortical progenitors, T cells as well as in the hematopoietic system causes Atf4 stabilization, which largely underlies the phenotypical defects in mice (Laguesse *et al*, 2015; Lemaitre *et al*, 2021; Rosu *et al*, 2021). We have not seen any robust Atf4 stabilization in peritoneal macrophages lacking Elp3 expression, suggesting that Atf4 stabilization is not a systematic consequence of *Elp3* deficiency in primary cells.

The capacity of Elp3 to prevent aberrant M1 macrophage polarization defines U_{34} chemical tRNA modifications as an anti-inflammatory process as *Elp3* deficiency in myeloid cells exacerbates inflammation in a model of experimental colitis. The defective Akt-dependent FoxO1 phosphorylation seen in LPS/IFNγ-stimulated peritoneal macrophages most likely contributes to this inflammatory phenotype seen upon *Elp3* deficiency. Therefore, Elp3 expression in myeloid cells prevents excessive inflammation in the large intestine, at least by promoting mTORC2 activation.

Loss of *Elp3* in macrophages impacts on mitochondrial functions. Indeed, IL-4-dependent metabolic reprogramming, a feature of M2 macrophage polarization, is largely impaired without Elp3. TCA cycle and mitochondrial oxidative phosphorylation are defective upon *Elp3* deficiency in macrophages. These phenotypes can be explained, at least in part, by a defective activation of both IL-4Rα-Stat6- and mTORC2-Irf4-dependent pathways, given their key roles in glucose consumption occurring during IL-4-dependent

 © 2022 The Authors

metabolic reprogramming (Huang *et al*, 2016). The fact that Elp3 promotes the production of some mitochondrial proteins of ribosome large subunit induced by IL-4 represents one molecular mechanism by which Elp3 controls mitochondrial functions in metabolic reprogramming.

Lastly, the genetic inactivation of *Elp3* in myeloid cells delays Wnt-driven tumor initiation in the intestine. This delay in tumor development also occurs when *Elp3* is inactivated in intestinal epithelial cells (Ladang *et al*, 2015). Although the role of Elp3 in all stromal cells remains to be extensively defined, our data strengthen the notion that Elp3 is a promising therapeutic target to treat intestinal tumors showing constitutive Wnt signaling.

Materials and Methods

Cell lines and reagents

RAW 264.7 cells were from ATCC (ATCC TIB-71) and were tested several times for mycoplasma contamination. They were cultured in DMEM medium supplemented with 10% FBS, 4.5 g/l of glucose, 2 mM of Glutamine, and 100 U/ml of Penicillin/Streptomycin. LPS was purchased from Sigma (L4391) and mouse IFNγ, IL-4, and IL-13 were from R&D Systems (485-MI, 404-ML, and 413-ML). Wortmannin (W1628) and AS1517499 (SML1906) were purchased from Sigma. Flag-Ric8b WT and Flag-Ric8b MUT were generated by VectorBuilder using the pLV-Puro-CMV/T7-EGFP as a lentiviral vector backbone.

Mice

The $Elp3^{lox/lox}$ mouse was previously described (Ladang *et al*, 2015). The LysM-CRE strain was obtained from Jackson Laboratory (stock 004781). $Elp3^{\Delta Mye}$ mice were obtained by crossing $Elp3^{lox/lox}$ with the LysM-CRE strain while $Elp3^{Control}$ mice correspond to $Elp3^{lox/lox}$ mice. The $Elp3^{\Delta IEC}$ mice in which Elp3 expression is genetically inactivated in intestinal epithelial cells has been previously described (Ladang *et al*, 2015). All mouse strains were housed at the animal facility of the University of Liege, according to rules requested by the ethical comity (file number 2332). Cages were ventilated, softly lit, and subjected to a light dark cycle. The relative humidity was kept at 45–65%. Mouse rooms and cages were always kept at a temperature range of 20–24°C. $Apc^{+/Min}$ mice were obtained from Jackson Laboratory (strain #:002020).

Experimental colitis model

Mice were given 2.5% DSS (molecular mass 36,000–50,000, MP Biomedicals, LLC) in the drinking water for 6 continuous days. Body weight was observed daily. Six days after the administration of DSS in water, colon length was measured and the colonic histological score was evaluated as follows: Inflammatory cell infiltrate severity (0–3) and extent (0–3), hyperplasia (0–2) and epithelial erosion (0–3), according to hematoxylin and eosin (H&E) staining samples, as previously described (Erben *et al*, 2014). mRNAs were extracted from the distal 1 cm of colon. For survival record, mice were given 2.5% DSS for 7 continuous days, then followed with normal water for another 8 days.

Generation of BMDMs

Bone marrow was extracted from femur and tibia, after lysis of erythrocytes, marrow cells were cultured in complete medium consisting of Dulbecco's modified Eagle's medium with 30% L929-conditioned medium and 10% heat-inactivated fetal bovine serum. On day 4, nonadherent cells were removed and the medium was replaced. On day 7, BMDMs were treated with stimulators. For infections of BMDMs, 3×10^6 293-LentiX cells were transfected with 12 μg of the "GFP" lentiviral plasmid (used as negative control), with Ric8b WT or Ric8b MUT, 12 μg of psPAX2 and 5 μg of VSVG plasmid, using the Mirus Bio's TransIT-LT1 reagent. Supernatants of those infected cells were collected and filtered (0.2 μm) 72 h after transfection and centrifuged at 90,000 *g* for 4 h. Pellets were diluted in BMDM growth media for 4 h at 4°C with constant rocking. Virus was added to BMDMs on the 6th day of differentiation with 8 μg/ml of polybrene. Twenty-four hours later, the media was changed and cells were left for another 96 h. After that, cells were collected and subjected to western blot analyses.

Treatment of mice with thioglycollate and peritoneal macrophage isolation

A 3% solution of thioglycollate (BD, Cat: 211716) was prepared in water, autoclaved and mice were received 1 ml intraperitoneal injection (i.p.) to induce peritonitis 4 days before sacrifice. Following sacrifice, peritoneal cavity exudate cells were obtained by washing the cavity with cold 1X PBS, erythrocytes were removed by incubating with a red blood cell lysis buffer. Cells were seeded in 6 well plates in the same culture medium as RAW 264.7 cells. Two hours later, cells were washed by PBS and then used for subsequent experiments.

IL-4 complex administration

Recombinant mouse IL-4 (BioLegend) was mixed with anti-mouse IL-4 monoclonal antibody (Biolegend, 11B11) at a molar ratio of 1:5 and incubated at room temperature (RT) for 5 min. Mice were injected i.p. with 100 μl of this cytokine-antibody complex (IL-4c) containing 5 μg of rIL-4 and 25 μg of anti-IL-4 antibody or PBS as control on day 0 and day 2. Peritoneal exudate cells were collected on day 4.

FACS sorting and intracellular staining

Peritoneal lavage was performed to collect exudates. Cells were then stained with primary antibodies in the Table EV1 before dead cells were excluded using a fixable viability dye (2000x, eFluor™ 780, eBiosciences Cat # 65-0865-14). A total of one million peritoneal macrophages were then sorted as Live F4/80^{+}CD11b^{+} cells using a FACSAria UIII (BD Biosciences) for further RNA extraction.

For Ki67 staining, the eBioscience FoxP3/Transcription Factor Staining Buffer set (00–5523–00) was used. In brief, after surface staining, cells were fixed with 1× Fix Concentrate buffer in the provided Fix Diluent for 30 min at room temperature. To permeabilize the cells, samples were washed with 1× Perm buffer diluted in water. Then, the samples were stained with secondary antibodies 20 min at room temperature, followed by washing in 1× Perm buffer and FACS buffer before flow cytometry.

© 2022 The Authors

For EdU staining, mice were given 1 mg of EdU i.p. (from Click-iT™ Plus EdU Flow Cytometry Assay Kit, Thermo Fisher, C10635) 3 h before sacrifice. After sacrifice of mice and peritoneal lavage, samples were processed, fixed. EdU-labeled cells were washed in 1× Perm buffer, incubated in Edu detection reaction cocktails for 30 min at room temperature, followed by washing in 1× Perm buffer and FACS buffer.

Histology and immunofluorescence microscopy

For histopathological analysis, colon samples were embedded in paraffin wax then sectioned (5 µm) and stained with H&E. For immunofluorescence, after dewaxing and hydration, antigen retrieval was performed by boiling slices for 30 min in a sodium citrate buffer pH 6.0. Slides were blocked with 10% goat serum for 1 h at RT and incubated with primary antibodies at 4°C overnight. After washing, Alexa Fluor® 568 goat anti- rabbit and Alexa Fluor® 488 goat anti-rat (Thermo Fisher) secondary antibody was applied to the slides at RT for 1 h. Then, sections were counterstained with 4′, 6-diamidino-2-phenylindole (DAPI). The antibody used in this study are listed in Table EV1. The immunofluorescence staining data were analyzed by using the Image-pro plus software.

RNA extraction and Real-Time PCR analysis

mRNAs expression was defined by reverse transcription polymerase chain reaction and RT-qPCR. RNAs were isolated from tissue or cells with the RNeasy Mini Kit (Qiagen) and then synthesized to DNAs (cDNAs) by using the RevertAid H Minus First Strand cDNA Synthesis Kit (Thermo Fisher) and augmented by using SYBR Green qPCR Mix (TAKARA) on LightCycler 480 (Roche). ΔCt values were normalized to GAPDH, and relative quantification of gene expression were compared to WT group. The primers used in this study are listed in Table EV2 and synthesized by Integrated DNA Technologies (Germany).

Immunoblotting

Macrophages were washed 3 times in cold PBS, lysed in RIPA buffer with protease and phosphatase inhibitor cocktails (Roche) for 10 min on a rocker 100 rpm at 4°C. Protein concentrations were quantified by using a BCA protein kit (Thermo Fisher). Proteins samples were analyzed on SDS polyacrylamide gel electrophoresis (SDS–PAGE) and transferred onto PVDF membranes (Millipore). These membranes were blocked with 5% BSA (Cell signaling) and 0.1% Tween 20 in Tris-buffered saline 1 h, and then incubated overnight at 4°C with primary antibodies. The appropriate HRP-coupled secondary antibody was then added and was detected with chemiluminescent substrate ECL (Thermo Fisher).

Quantification of tRNA modifications

BMDMs were collected and cultured from three $Elp3^{Control}$ and $Elp3^{\Delta Mye}$ mice. Replicates were used for each mouse. Total RNAs were extracted using Trizol. RNAs were submitted to Arraystar (arraystar.com) for LC–MS analysis where it was processed as follows: total RNA samples were quantified by using Nanodrop ND-1000. tRNAs were isolated from total RNAs by Urea-PAGE and 60-90 nt bands of tRNAs were excised and purified by ethanol precipitation. tRNAs were hydrolyzed to single dephosphorylated nucleosides by enzyme mix. Pretreated nucleoside solutions were deproteinized using Satorius 10,000-Da MWCO spin filter. The analysis of nucleoside mixtures was performed on Agilent 6460 QQQ mass spectrometer with an Agilent 1260 HPLC system using the Multi reaction monitoring (MRM) detection mode. LC–MS data were acquired using Agilent Qualitative Analysis software. MRM peaks of each modified nucleoside were extracted and normalized.

Northern blots

Five micrograms of total RNAs (or 1 µg of enriched small RNAs isolated using the kit mirVana™ Paris™ RNA (Invitrogen)) extracted from peritoneal macrophages were subjected to electrophoresis on a 8% acrylamide gel (29:1) with 6 M Urea and 1 × TBE. Fifty micrograms per milliliter of N-acryloylaminophenyl mercuric chloride (APM) were added or not. RNAs were transferred on a positively charged nylon membrane (180 mA, 45 min, 0.5 × TBE) using the Trans Blot Turbo Transfer system. RNAs were crosslinked using a Spectrolinker UV crosslinker (Invitrogen). Hybridization was performed using the Easy-Hyb solution (Roche). The probe dQ(UUG) GGTCCCACCGAGATTT-GAACTCGG, tK(UUU)CCTGAACAGGGACT TGAACCCTGA-3 or tE(UUC) GTTCCCACACCGGGAGTC-GAAC was labeled using the DIG Oligonucleotide 3′ end labeling kit (second generation) and hybridized overnight at 50°C with constant rotation (for the probe tK(UUU), the hybridization was performed at 45°C). Membranes were washed twice with a buffer containing 2 × SSC, 0.1% SDS at room temperature during 5 min and twice with another buffer containing 0.2 × SSC, 0.1% SDS at 50°C (45°C for the probe tK(UUU) during 15 min. The detection of both unmodified and thiolated tRNAs was performed using DIG Luminescent Detection Kit for Nucleic Acids.

ChIP

ChIP assays were essentially performed by using the anti-STAT6 antibody (Cell signaling) or an IgG antibody as negative control. Extracts from PBS or IL-4-treated RAW 264.7 cells were precleared by incubation with protein A Sepharose/Herring sperm DNA for 1 h and subsequent IPs were performed by incubating cell extracts overnight at 4°C with the relevant antibody followed by 1 h of incubation with protein A/Herring sperm DNA. Protein–DNA complexes were washed as per standard ChIP techniques. After elution, proteinase K treatment and reversal of crosslinks, DNA fragments were analyzed by real-time PCR with SYBR green detection. Input DNA was analyzed simultaneously and used for normalization purposes. Primers used to address STAT6 recruitment on the *Elp3* gene promoter are listed in the Table EV2. Putative STAT6-binding sites were predicted by JASPAR database (http://jaspar.genereg.net/).

Transfection of siRNA

Cells were transfected with ON-TARGETplus Mouse Raptor, Rictor, Ric8b, and siGENOME mouse Elp3 and Ctu2 siRNAs (Horizon Discovery) or control siRNAs by using INTERFERin (Polplus) according to the manufacturer's instruction. RAW264.7 cells and BMDMs were transfected with 50 nmol siRNA and 10 µl of transfection reagent on

 © 2022 The Authors

three consecutive days and experiments were performed 96 h after the first transfection. The siRNAs and their sequences used in this study are listed in Table EV3.

RNA sequencing and analysis

BMDMs from *Elp3*^{Control} and *Elp3*$^{\Delta Mye}$ mice were treated with or without IL-4 and IL-13 for 2 h. A total of 6 mice were used for each experimental condition. Total RNAs were extracted using a RNeasy Mini Kit (Qiagen) according to the manufacturer's instructions. RNA quality was controlled on the Bioanalyser 2100 (agilent). TruSeq mRNA library preparation started from 1 μg of total RNA and denatured for 4 min. Libraries were validated by qPCR using the KAPA Universal kit (KAPA Biosystems). Libraries were sequenced on the NExtSeq550. We employed GSEA in the context of gene ontology and utilized specific genes sets (GEO: GSE7348 and GSE25123) to perform GSEA. The heatmap was generated using https://software.broadinstitute.org/morpheus. Differential gene expression was calculated using DESeq2 in R. Genes with normalized P values < 0.05 were considered as significantly regulated.

LC–MS profiling for intracellular metabolites

For metabolomics analysis of peritoneal macrophages, the peritoneal macrophages from *Elp3*^{Control} and *Elp3*$^{\Delta Mye}$ mice were treated with or without IL-4 and IL-13 for 24 h. Each sample was washed three times with cold PBS, collected into an Eppendorf tube, frozen in liquid nitrogen and stored at −80°C until extraction. The extraction solution used was 50% methanol, 30% ACN, and 20% water. The volume of extraction solution added was calculated from the cell count (2×10^6 cells/ml). After addition of extraction solution, samples were vortexed for 5 min at 4°C, and immediately centrifuged at 16,000 *g* for 15 min at 4°C. The supernatants were collected and analyzed by liquid chromatography–mass spectrometry using SeQuant ZIC-pHilic column (Merck) for the liquid chromatography separation. Mobile phase A consisted of 20 mM of ammonium carbonate plus 0.1% ammonia hydroxide in water. Mobile phase B consisted of ACN. The flow rate was kept at 100 ml/min, and the gradient was 0 min, 80% of B; 30 min, 20% of B; 31 min, 80% of B; and 45 min, 80% of B. The mass spectrometer (QExactive Orbitrap, Thermo Fisher Scientific) was operated in a polarity switching mode and metabolites were identified using TraceFinder Software (Thermo Fisher Scientific). To obtain a robust statistical analysis, metabolomics data were normalized using the median normalization method (Hendriks *et al*, 2007). The data were further pre-processed with a log transformation. The MetaboAnalyst 4.0 software (Xia *et al*, 2015) was used to conduct statistical analysis and heatmap generation, and enrichment analysis. The algorithm for heatmap clustering was based on the Pearson distance measure for similarity and the Ward linkage method for biotype clustering. Metabolites with similar abundance patterns were positioned closer together.

Glucose consumption assay

Glucose-Glo™ Assay Kit (Promega) was used to determine glucose uptake according to the manufacturer's protocols. IL-4-primed M2 peritoneal macrophages from *Elp3*^{Control} and *Elp3*$^{\Delta Mye}$ mice were plated at 2.5×10^5 cells/ml into 24-well plates and incubated for 24 h. Next day, cells incubation mediums and medium without cells (empty control) were collected and used for determination of glucose consumption. The luminescence was recorded by PerkinElmer Victor X3. The glucose consumption was calculated as the difference between the noncells medium value and cells incubation medium value.

Complex I enzyme activity assay

The Complex I enzyme activity was performed by following instructions of the Complex I Enzyme Activity Microplate Assay Kit (Abcam, ab109721). Briefly, 1×10^7 peritoneal macrophages from *Elp3*^{Control} and *Elp3*$^{\Delta Mye}$ mice were seeded in 15 cm^2 dishes with IL-4 treatment for 24 h. The cell lysis buffer was added into the microplate and incubated at room temperature for 3 h. After 3 washes, assay solutions were added and then the data were recorded as the following settings: wavelength, 450 nm; time, 30 min; interval, 20 s. The rate was calculated by the formula: mOD/min = Abs 1 – Abs 2 (mOD @ 450 nm)/Time 1 – Time 2 (minutes).

Mitochondrial membrane potential

Peritoneal macrophages from *Elp3*^{Control} and *Elp3*$^{\Delta Mye}$ mice were plated at 5×10^5 cells/ml in 12-well plates and treated with IL-4 for 24 h. The following control cell samples were accounted for: unstained, single stained TMRM (Thermo Fisher), MitoTracker Deep Red (Thermo Fisher, hereafter MTDR), and Viability 405/452 Fixable Dye (Miltenyi Biotech). Cells were stained with Viability 405/452 Fixable Dye at 37°C in the dark for 30 min. Next, cells were stained by a mix of MTDR (50 nM) and TMRM (20 nM) at 37°C in the dark for another 30 min. Cells were removed from the plate surface using a cell scraper and transferred to polypropylene FACS tubes. Cells were then analyzed using BD FACS Canto II, and data were analyzed using FlowJo software.

Extracellular flux assays

All experiments were performed with a Seahorse XFp extracellular flux analyzer (Agilent). Cells were seeded (100,000 cells/well) in XFp mini-plates (Agilent) and treated by IL-4 for 24 h. One hour before the mitochondrial oxygen consumption rate (OCR - pmol/min) analysis, culture medium was replaced by an unbuffered serum-free DMEM (Basal DMEM, Agilent) supplemented with pyruvate (1 mM), glutamine (2 mM), glucose (10 mM), pH7.4, and further incubated at 37°C in ambient CO_2 incubator. During the assay, cells were successively stressed with oligomycin (1 μM), FCCP (1.0 μM) and rotenone/antimycin A (0.5 μM each) mix. Spare OCR capacity was calculated as the difference between the average of the 3 pre-oligomycin measurements and the average of the 3 post-FCCP measurements. All results were normalized according the cell number evaluated by Hoechst (2 μg/ml) incorporation (A.U.) after cold methanol/acetone fixation. Results shown are representative of 3 independent experiments.

Puromycin proximity ligation assay (Puro-PLA)

IL-4-activated BMDMs were treated with Puromycin (10 μg/ml) for 5 min at 37°C, 5% CO_2, and washed twice in PBS. As negative

© 2022 The Authors

control, cells were treated with Cycloheximide (100 μg/ml) before Puromycin treatment. Cells were fixed in 4% paraformaldehyde for 20 min and permeabilized with 0.5% Triton X-100 solution for 1 h. Duolink PLA reagents (Sigma-Aldrich) were used to detect newly synthesized proteins according to the manufacturer's instructions. Briefly, cells were blocked using the Duolink Blocking solution for 1 h at room temperature and then were incubated in the primary antibody (anti-Ric8b) overnight at 4°C. After several washings, cells were incubated in the PLUS and MINUS PLA probes solutions for 1 h at 37°C. Next, cells were incubated in the ligation solution (30 min) and in the amplification buffer (100 min) at 37°C. Once the amplification was done, cells were incubated in the anti-α-tubulin antibody solution overnight and then incubated in the secondary antibody solution for 1 h. Glasses were mounted with the fluorescent mounting medium overnight. Duolink PLA signals were visualized by fluorescence microscopy (Zeiss HR LSM 880).

Aggresome detection

BMDMs were treated with IL-4 for 24 h and the positive control was treated by MG-132. Then, the procedures were followed in agreement with the instruction of the PROTEOSTAT® Aggresome Detection Kit (Enzo, ENZ-51035-K100). Briefly, cells were washed by PBS twice and then incubated in 4% formaldehyde for 30 min. Cells were then washed with PBS and incubated in the Permeabilizing Solution for 30 min and lastly incubated in the Dual Detection Reagent for another 30 min in the dark. The samples were analyzed in the FL3 channel of a flow cytometer.

Aggregates isolation

BMDMs were treated with IL-4 or PBS for 24 h and cells were lysed using the Cytoplasmic Lysis Buffer (Tris HCl pH 7.9, 10 mM; sucrose 340 nM; $CaCl_2$ 3 mM; EDTA 0.1 mM; $MgCl_2$ 2 mM; 1 mM DTT; 0.5% NP-40) for 10 min on ice. The samples were then centrifuged at 1,100 *g*, 4°C for 15 min. The supernatant was centrifuged at 20,000 *g*, 4°C for 10 min. Protein concentration in supernatants was determined with the Bio-Rad Protein Assay. Protein concentration was equalized across samples and aggregates were pelleted from equal amounts of total proteins. Pellets were sonicated 4 times for 8 min in the cold room. The samples were then centrifuged at 16,000 *g*, at 4°C for 20 min and the pellets were washed using the washing buffer (NaF 20 mM pH 6.8; PMSF 1 μM) with 2% NP-40 twice and next washed using the washing buffer without NP-40 one more time. Lastly, the samples were sonicated for 4 min and centrifuged at 16,000 *g*, at 4°C for 20 min. Supernatants were collected as aggregates fraction.

LC–MS profiling for total proteins and aggregates proteins

The total proteins were extracted from *Elp3*^{Control} and *Elp3*$^{\Delta Mye}$ BMDMs treated with or without IL-4 (20 ng/ml) for 4 h. Lysates were quantified by Qubit fluorometry. The aggregate extraction samples were heated at 100°C for 15 min in the loading buffer, centrifuged briefly, and 50% was loaded on gel. Each gel lane was excised into 20 equally sized segments and gel slices were processed using a robot (ProGest, DigiLab) with the following protocol: washed with 25 mM ammonium bicarbonate followed by acetonitrile; reduced with 10 mM dithiothreitol at 60°C followed by alkylation with 50 mM iodoacetamide at room temperature; digested with trypsin (Promega) at 37°C for 4 h; quenched with formic acid and the supernatant was analyzed directly without further processing. Peptides were analyzed by nano LC/MS/MS with a Waters NanoAcquity HPLC system interfaced to a Thermo Fisher Fusion Lumos. Peptides were loaded on a trapping column and eluted over a 75 μm analytical column at 350 nL/min; both columns were packed with Luna C18 resin (Phenomenex). The mass spectrometer was operated in data-dependent mode, with MS and MS/MS performed in the Orbitrap at 60,000 FWHM resolution and 15,000 FWHM resolution, respectively. APD was turned on. The instrument was run with a 3 s cycle for MS and MS/MS. Mascot DAT files were parsed into the Scaffold software for validation, filtering and to create a nonredundant list per sample. Data were filtered 1% protein and peptide level FDR and requiring at least two unique peptides per protein.

Codon bias analysis

The coding sequence (cds) of all protein-coding Mus musculus genes (genome assembly GRCm38.p6) was downloaded from Ensembl (http://www.ensembl.org). Codon frequency of AAA, CAA, and GAA in the complete cds of every Mouse Genome Informatics–annotated mouse gene was computed using the seqinr and biomaRt packages in R. We next computed a z-score for AAA, CAA, and GAA codon enrichment/ depletion in the cds of proteins found to be downregulated in BMDMs from *Elp3*$^{\Delta Mye}$ mice in our comparative proteomic analysis using the formula: z = (codon frequency of gene − average codon frequency in all mouse cds)/variance of codon frequency in all mouse cds. A heatmap was subsequently generated using the heatmap package in R.

Statistical analysis

The statistical significance of differences in groups were performed using the GraphPad Prism 8.0 Software. All data and error bars are presented as the mean ± standard deviation (SD) and based on experiments performed at least in triplicate. The one- or two-way ANOVA test was used to compare the mean of a continuous variable between two samples and Unpaired Student's *t*-test was used to comparison between two groups. $P < 0.05$ was considered to be statistically significant. Blinding was systematically done.

Data availability

RNA-seq data has been deposited on Gene Expression Omnibus (GEO) under accession number GSE166169 (http://www.ncbi.nlm.nih.gov/geo/query/acc.cgi?acc=GSE166169). Proteomic data has been deposited on ProteomeXChange with accession code PXD033962 and PXD023588 (http://www.ebi.ac.uk/pride/archive/projects/PXD033962; http://www.ebi.ac.uk/pride/archive/projects/PXD023588). Other data that support the findings of this study are available from the corresponding authors upon request.

Expanded View for this article is available online.

 © 2022 The Authors

Acknowledgments

The authors are grateful to the GIGA Imaging platform for the FACS analyses. They also thank Dr. Zhang from NIBIO (Olso, Norway) for providing us R scripts on RNA-seq and proteomics analysis. This work was supported by grants from WELBIO (WELBIO-CR-2019C-02R), FNRS/TELEVIE, the Belgian Foundation against Cancer (FBC/2020/1323), the University of Liege (ARC UBICOREAR) and by the Leon Fredericq Foundation (Faculty of Medicine, CHU of Liege). PC and AC are Senior Research Associate and Research Director at the FNRS, respectively.

Author contributions

Dawei Chen: Conceptualization; formal analysis; validation; investigation. **Ivan Nemazanyy:** Investigation; methodology. **Olivier Peulen:** Investigation; methodology. **Kateryna Shostak:** Data curation; formal analysis; supervision; validation; investigation; methodology. **Xinyi Xu:** Formal analysis; methodology. **Seng Chuan Tang:** Formal analysis; methodology. **Caroline Wathieu:** Data curation; methodology. **Silvia Turchetto:** Formal analysis; methodology. **Sylvia Tielens:** Data curation; methodology. **Laurent Nguyen:** Conceptualization; formal analysis; methodology. **Pierre Close:** Methodology. **Christophe Desmet:** Conceptualization; resources. **Sebastian Klein:** Conceptualization; data curation; formal analysis; methodology. **Alexandra Florin:** Data curation; methodology. **Reinhard Büttner:** Conceptualization; data curation; methodology. **Georgios Petrellis:** Data curation; formal analysis. **Benjamin Dewals:** Conceptualization; data curation; formal analysis; supervision; validation; investigation; methodology; writing – review and editing. **Alain Chariot:** Conceptualization; resources; data curation; formal analysis; supervision; funding acquisition; validation; investigation; visualization; methodology; writing – original draft; project administration; writing – review and editing.

Disclosure and competing interests statement

The authors declare that there is no conflict of interest.

References

Bauer F, Matsuyama A, Candiracci J, Dieu M, Scheliga J, Wolf DA, Yoshida M, Hermand D (2012) Translational control of cell division by elongator. *Cell Rep* 1: 424 – 433

Brown J, Wang H, Suttles J, Graves DT, Martin M (2011) Mammalian target of rapamycin complex 2 (mTORC2) negatively regulates toll-like receptor 4-mediated inflammatory response via FoxO1. *J Biol Chem* 286: 44295 – 44305

Brunet A, Bonni A, Zigmond MJ, Lin MZ, Juo P, Hu LS, Anderson MJ, Arden KC, Blenis J, Greenberg ME (1999) Akt promotes cell survival by phosphorylating and inhibiting a forkhead transcription factor. *Cell* 96: 857 – 868

Byles V, Covarrubias AJ, Ben-Sahra I, Lamming DW, Sabatini DM, Manning BD, Horng T (2013) The TSC-mTOR pathway regulates macrophage polarization. *Nat Commun* 4: 2834

Candiracci J, Migeot V, Chionh YH, Bauer F, Brochier T, Russell B, Shiozaki K, Dedon P, Hermand D (2019) Reciprocal regulation of TORC signaling and tRNA modifications by elongator enforces nutrient-dependent cell fate. *Sci Adv* 5: eaav0184

Cassado AA, D'Império Lima MR, Bortoluci KR (2015) Revisiting mouse peritoneal macrophages: Heterogeneity, development, and function. *Front Immunol* 6: 225

Cruz-Gordillo P, Honeywell ME, Harper NW, Leete T, Lee MJ (2020) ELP-dependent expression of MCL1 promotes resistance to EGFR inhibition in triple-negative breast cancer cells. *Sci Signal* 13: eabb9820

Delaunay S, Rapino F, Tharun L, Zhou Z, Heukamp L, Termathe M, Shostak K, Klevernic I, Florin A, Desmecht H *et al* (2016) Elp3 links tRNA modification to IRES-dependent translation of LEF1 to sustain metastasis in breast cancer. *J Exp Med* 213: 2503 – 2523

Dewez M, Bauer F, Dieu M, Hermand D (2008) The conserved wobble uridine tRNA thiolase Ctu1–Ctu2 is required to maintain genome integrity. *Proc Natl Acad Sci U S A* 105: 5459 – 5464

Düvel K, Yecies JL, Menon S, Raman P, Lipovsky AI, Souza AL, Triantafellow E, Ma Q, Gorski R, Cleaver S *et al* (2010) Activation of a metabolic gene regulatory network downstream of mTOR complex 1. *Mol Cell* 39: 171 – 183

El Yacoubi B, Bailly M, De Crécy-Lagard V (2012) Biosynthesis and function of posttranscriptional modifications of transfer RNAs. *Annu Rev Genet* 46: 69 – 95

Erben U, Loddenkemper C, Doerfel K, Spieckermann S, Haller D, Heimesaat MM, Zeitz M, Siegmund B, Kühl AA (2014) A guide to histomorphological evaluation of intestinal inflammation in mouse models. *Int J Clin Exp Pathol* 7: 4557 – 4576

Festuccia WT, Pouliot P, Bakan I, Sabatini DM, Laplante M (2014) Myeloid-specific rictor deletion induces M1 macrophage polarization and potentiates *in vivo* pro-inflammatory response to lipopolysaccharide. *PLoS One* 9: e95432

Galdiero MR, Garlanda C, Jaillon S, Marone G, Mantovani A (2013) Tumor associated macrophages and neutrophils in tumor progression. *J Cell Physiol* 228: 1404 – 1412

Gordon S, Martinez FO (2010) Alternative activation of macrophages: mechanism and functions. *Immunity* 32: 593 – 604

Hallowell RW, Collins SL, Craig JM, Zhang Y, Oh M, Illei PB, Chan-Li Y, Vigeland CL, Mitzner W, Scott AL *et al* (2017) MTORC2 signalling regulates M2 macrophage differentiation in response to helminth infection and adaptive thermogenesis. *Nat Commun* 8: 14208

Hendriks MMWB, Smit S, Akkermans WLMW, Reijmers TH, Eilers PHC, Hoefsloot HCJ, Rubingh CM, De Koster CG, Aerts JM, Smilde AK (2007) How to distinguish healthy from diseased? Classification strategy for mass spectrometry-based clinical proteomics. *Proteomics* 7: 3672 – 3680

Hou J, Schindler U, Henzel WJ, Ho TC, Brasseur M, McKnight SL (1994) An interleukin-4-induced transcription factor: IL-4 stat. *Science* 265: 1701 – 1706

Howell JJ, Manning BD (2011) MTOR couples cellular nutrient sensing to organismal metabolic homeostasis. *Trends Endocrinol Metab* 22: 94 – 102

Huang SCC, Smith AM, Everts B, Colonna M, Pearce EL, Schilling JD, Pearce EJ (2016) Metabolic reprogramming mediated by the mTORC2-IRF4 signaling axis is essential for macrophage alternative activation. *Immunity* 45: 817 – 830

Jenkins SJ, Ruckerl D, Cook PC, Jones LH, Finkelman FD, van Rooijen N, MacDonald AS, Allen JE (2011) Local macrophage proliferation, rather than recruitment from the blood, is a signature of TH2 inflammation. *Science* 332: 1284 – 1288

Jha AK, Huang SCC, Sergushichev A, Lampropoulou V, Ivanova Y, Loginicheva E, Chmielewski K, Stewart KM, Ashall J, Everts B *et al* (2015) Network integration of parallel metabolic and transcriptional data reveals metabolic modules that regulate macrophage polarization. *Immunity* 42: 419 – 430

Kim JH, Lee E, Friedline RH, Suk S, Jung DY, Dagdeviren S, Hu X, Inashima K, Noh HL, Kwon JY *et al* (2018) Endoplasmic reticulum chaperone GRP78 regulates macrophage function and insulin resistance in diet-induced obesity. *FASEB J* 32: 2292 – 2304

Ladang A, Rapino F, Heukamp LC, Tharun L, Shostak K, Hermand D, Delaunay S, Klevernic I, Jiang Z, Jacques N *et al* (2015) Elp3 drives Wnt-dependent

tumor initiation and regeneration in the intestine. *J Exp Med* 212: 2057 – 2075

Laguesse S, Creppe C, Nedialkova DD, Prévot PP, Borgs L, Huysseune S, Franco B, Duysens G, Krusy N, Lee G *et al* (2015) A dynamic unfolded protein response contributes to the control of cortical neurogenesis. *Dev Cell* 35: 553 – 567

Laplante M, Sabatini DM (2012) MTOR signaling in growth control and disease. *Cell* 149: 274 – 293

Lemaitre P, Bai Q, Legrand C, Chariot A, Close P, Bureau F, Desmet CJ (2021) Loss of the transfer RNA wobble uridine–modifying enzyme Elp3 delays T cell cycle entry and impairs T follicular helper cell responses through deregulation of Atf4. *J Immunol* 206: 1077 – 1087

Liu GY, Sabatini DM (2020) mTOR at the nexus of nutrition, growth, ageing and disease. *Nat Rev Mol Cell Biol* 21: 183 – 203

Liu PS, Wang H, Li X, Chao T, Teav T, Christen S, Di Conza G, Cheng WC, Chou CH, Vavakova M *et al* (2017) A-ketoglutarate orchestrates macrophage activation through metabolic and epigenetic reprogramming. *Nat Immunol* 18: 985 – 994

Lumeng CN, Bodzin JL, Saltiel AR (2007) Obesity induces a phenotypic switch in adipose tissue macrophage polarization. *J Clin Invest* 117: 175 – 184

Murray PJ (2017) Macrophage polarization. *Annu Rev Physiol* 79: 541 – 566

Murray PJ, Wynn TA (2011) Protective and pathogenic functions of macrophage subsets. *Nat Rev Immunol* 11: 723 – 737

Murray PJ, Allen JE, Biswas SK, Fisher EA, Gilroy DW, Goerdt S, Gordon S, Hamilton JA, Ivashkiv LB, Lawrence T *et al* (2014) Macrophage activation and polarization: Nomenclature and experimental guidelines. *Immunity* 41: 14 – 20

Nagai MH, Xavier VPS, Gutiyama LM, Machado CF, Reis AH, Donnard ER, Galante PAF, Abreu JG, Festuccia WT, Malnic B (2020) Depletion of Ric-8B leads to reduced mTORC2 activity. *PLoS Genet* 16: e1008255

Nedialkova DD, Leidel SA (2015) Optimization of codon translation rates via tRNA modifications maintains proteome integrity. *Cell* 161: 1606 – 1618

O'Neill LAJ (2016) A metabolic roadblock in inflammatory macrophages. *Cell Rep* 17: 625 – 626

Pulendran B, Artis D (2012) New paradigms in type 2 immunity. *Science* 337: 431 – 435

Rapino F, Delaunay S, Zhou Z, Chariot A, Close P (2017) tRNA modification: is cancer having a wobble? *Trends Cancer* 3: 249 – 252

Rapino F, Delaunay S, Rambow F, Zhou Z, Tharun L, De Tullio P, Sin O, Shostak K, Schmitz S, Piepers J *et al* (2018) Codon-specific translation reprogramming promotes resistance to targeted therapy. *Nature* 558: 605 – 609

Rodríguez-Prados J-C, Través PG, Cuenca J, Rico D, Aragonés J, Martín-Sanz P, Cascante M, Boscá L (2010) Substrate fate in activated macrophages: a comparison between innate, classic, and alternative activation. *J Immunol* 185: 605 – 614

Rosu A, El Hachem N, Rapino F, Rouault-Pierre K, Jorssen J, Somja J, Ramery E, Thiry M, Nguyen L, Jacquemyn M *et al* (2021) Loss of tRNA-modifying enzyme Elp3 activates a p53-dependent antitumor checkpoint in hematopoiesis. *J Exp Med* 218: e20200662

Rubio RM, Depledge DP, Bianco C, Thompson L, Mohr I (2018) RNA m 6 a modification enzymes shape innate responses to DNA by regulating interferon β. *Genes Dev* 32: 1472 – 1484

Ryan DG, O'Neill LAJ (2020) Krebs cycle reborn in macrophage immunometabolism. *Annu Rev Immunol* 38: 289 – 313

Saha S, Shalova IN, Biswas SK (2017) Metabolic regulation of macrophage phenotype and function. *Immunol Rev* 280: 102 – 111

Shulman Z, Stern-Ginossar N (2020) The RNA modification N 6-methyladenosine as a novel regulator of the immune system. *Nat Immunol* 21: 501 – 512

Stein M, Keshav S, Harris N, Gordon S (1992) Interleukin 4 potently enhances murine macrophage mannose receptor activity: a marker of alternative immunologic macrophage activation. *J Exp Med* 176: 287 – 292

Su LK, Kinzler KW, Vogelstein B, Preisinger AC, Moser AR, Luongo C, Gould KA, Dove WF (1992) Multiple intestinal neoplasia caused by a mutation in the murine homolog of the APC gene. *Science* 256: 668 – 670

Tavares JF, Davis NK, Poim A, Reis A, Kellner S, Sousa I, Soares AR, Moura GMR, Dedon PC, Santos M (2020) tRNA-modifying enzyme mutations induce codon-specific mistranslation and protein aggregation in yeast. *RNA Biol* 18: 563 – 575

Vats D, Mukundan L, Odegaard JI, Zhang L, Smith KL, Morel CR, Greaves DR, Murray PJ, Chawla A (2006) Oxidative metabolism and PGC-1β attenuate macrophage-mediated inflammation. *Cell Metab* 4: 13 – 24

Wang J-M, Chao J-R, Chen W, Kuo M-L, Yen JJ-Y, Yang-Yen H-F (1999) The antiapoptotic gene mcl-1 is up-regulated by the phosphatidylinositol 3-kinase/Akt signaling pathway through a transcription factor complex containing CREB. *Mol Cell Biol* 19: 6195 – 6206

Winkler R, Gillis E, Lasman L, Safra M, Geula S, Soyris C, Nachshon A, Tai-Schmiedel J, Friedman N, Le-Trilling VTK *et al* (2019) M 6 a modification controls the innate immune response to infection by targeting type I interferons. *Nat Immunol* 20: 173 – 182

Xia J, Sinelnikov IV, Han B, Wishart DS (2015) MetaboAnalyst 3.0-making metabolomics more meaningful. *Nucleic Acids Res* 43: W251 – W257

Yano T, Ferlito M, Aponte A, Kuno A, Miura T, Murphy E, Steenberger C (2014) Pivotal role of mTORC2 and involvement of ribosomal protein S6 in cardioprotective signaling. *Circ Res* 114: 1268 – 1280

License: This is an open access article under the terms of the Creative Commons Attribution License, which permits use, distribution and reproduction in any medium, provided the original work is properly cited.

Expanded View Figures

Figure EV1. Expression of Elongator subunits and Ctu1/2 by M1 polarization signals.

A, B M1 polarization signals downregulate the expression of tRNA-modifying enzymes at both mRNA (A) and protein (B) levels. Peritoneal macrophages were untreated or stimulated with IFNγ (50 ng/ml), LPS (100 ng/ml) or with both IFNγ and LPS *ex-vivo* for the indicated periods of time and mRNA levels of the indicated candidates were assessed in all experimental conditions by Real-Time PCR (A). mRNA levels in unstimulated cells were set to 1 and levels in other experimental conditions were relative to that after normalization with Gapdh mRNA levels (n = 3 mice; mean ± SD, Student t-test, *$P < 0.05$; **$P < 0.01$; ***$P < 0.001$). Protein levels (B) were assessed by western blot analyses using the indicated antibodies.

C BMDMs and intestinal epithelial cells (IECs) lacking *Elp3* show decreased thiolated tRNA levels. Northern blots were carried out with enriched small RNAs extracted from BMBMs of $Elp3^{Control}$ and $Elp3^{\Delta Mye}$ mice or with total RNAs from IECs of $Elp3^{Control}$ and $Elp3^{\Delta IEC}$ mice, using the indicated probes. Values are from three independent measurements (mean ± SD, ****$P < 0.0001$, Welch one-way Anova test).

D Elp3 expression in myeloid cells is dispensable in the architecture of intestinal crypts. Colons from both $Elp3^{Control}$ and $Elp3^{\Delta Mye}$ mice were subjected to IHC analyses.

E Colon length is not altered upon *Elp3* deficiency in myeloid cells. Data at day 6 from 8 mice per group are illustrated (mean ± SD, Student t-test, nonsignificant).

F mRNA levels of pro-inflammatory cytokines in peritoneal macrophages lacking Elp3 do not change in unstimulated mice. mRNAs of the listed pro-inflammatory cytokines in peritoneal macrophage from $Elp3^{Control}$ or $Elp3^{\Delta Mye}$ mice were assessed by Real Time PCR. mRNA levels in $Elp3^{Control}$ mice were set to 1 and levels in other experimental conditions were relative to that after normalization with Gapdh mRNA levels (n = 5 mice; mean ± SD, nonsignificant).

Source data are available online for this figure.

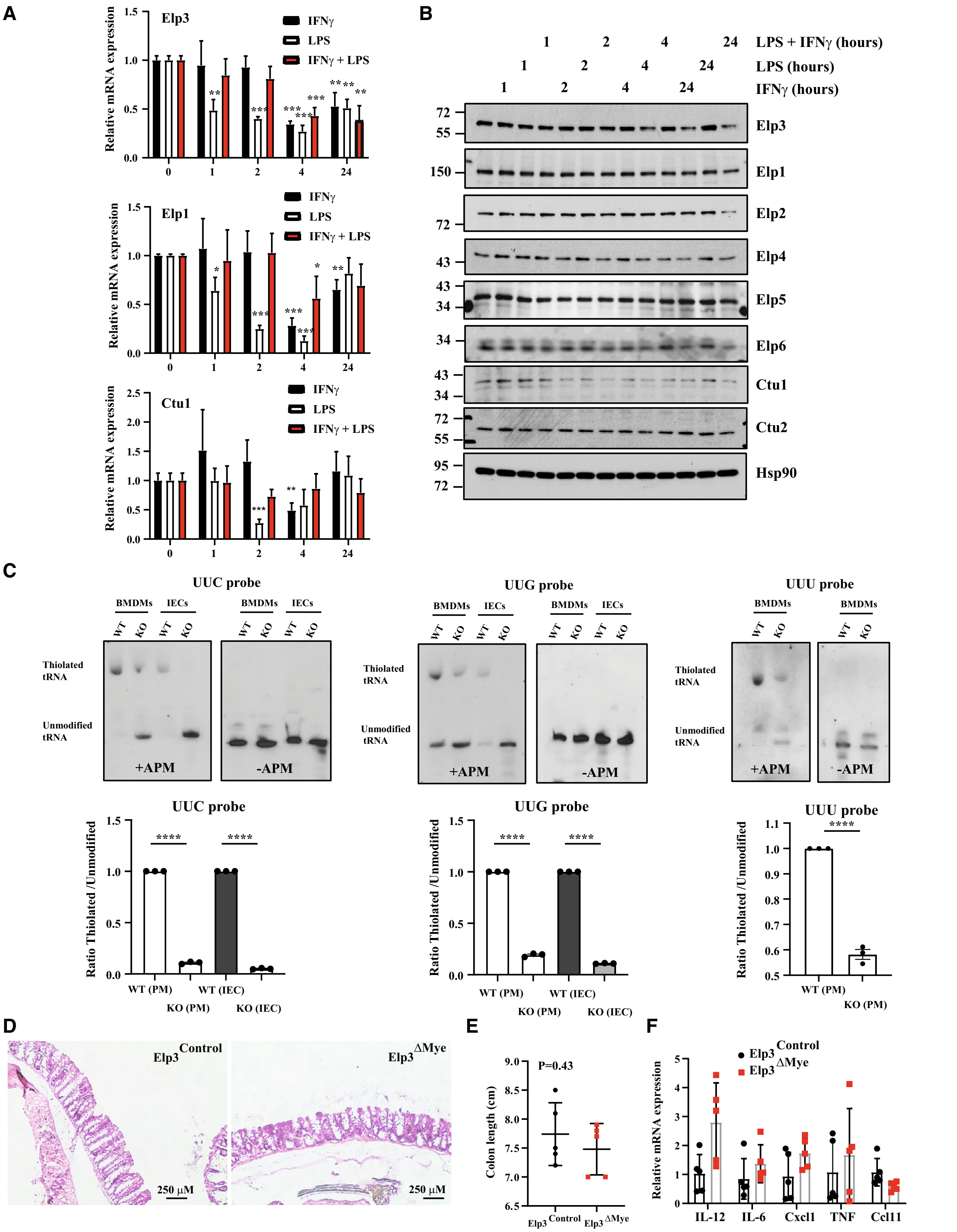

A
Elp3
Elp1
Ctu1
IFNγ
LPS
IFNγ + LPS
Relative mRNA expression
B
LPS + IFNγ (hours)
LPS (hours)
IFNγ (hours)
Elp3
Elp1
Elp2
Elp4
Elp5
Elp6
Ctu1
Ctu2
Hsp90
C
UUC probe
UUG probe
UUU probe
BMDMs
IECs
Thiolated tRNA
Unmodified tRNA
+APM
-APM
Ratio Thiolated /Unmodified
WT (PM)
KO (PM)
WT (IEC)
KO (IEC)
D
Elp3 Control
Elp3 ΔMye
250 µM
E
P=0.43
Colon length (cm)
F
Relative mRNA expression
IL-12
IL-6
Cxcl1
TNF
Ccl11

Figure EV1.

Figure EV2. Ctu2 promotes IL-4-dependent mTORC2 activation and M2 macrophage polarization.

A Elp1/3 and Ctu1/2 expression are induced by M2 polarization signals. Peritoneal macrophages were untreated or stimulated with IL-4 (20 ng/ml), IL-13 (20 ng/ml) or with both IL-4 and IL-13 *ex-vivo* for the indicated periods of time and mRNA levels of the indicated candidates were assessed in all experimental conditions by Real-Time PCR. mRNA levels in unstimulated cells were set to 1 and levels in other experimental conditions were relative to that after normalization with Gapdh mRNA levels ($n = 3$ mice; mean ± SD, Student t-test, *$P < 0.05$; **$P < 0.01$; ***$P < 0.001$).

B IL-4-dependent mTORC2 activation relies on Ctu2. Control and Ctu2-depleted bone marrow-derived macrophages (BMDMs) were treated or not with IL-4 (10 ng/ml) and the resulting cell extracts were subjected to western blot (WB) analyses.

C Ctu2 deficiency impairs M2 macrophage polarization. Control and Ctu2-depleted BMDMs were treated or not with IL-4 (10 ng/ml) for 24 h and the resulting mRNAs of the listed M2 markers were assessed by Real Time PCR. mRNA levels in control BMDMs were set to 1 and levels in other experimental conditions were relative to that after normalization with Gapdh mRNA levels ($n = 3$ mice; mean ± SD, Student t-test, *$P < 0.05$, **$P < 0.01$, ***$P < 0.001$).

Source data are available online for this figure.

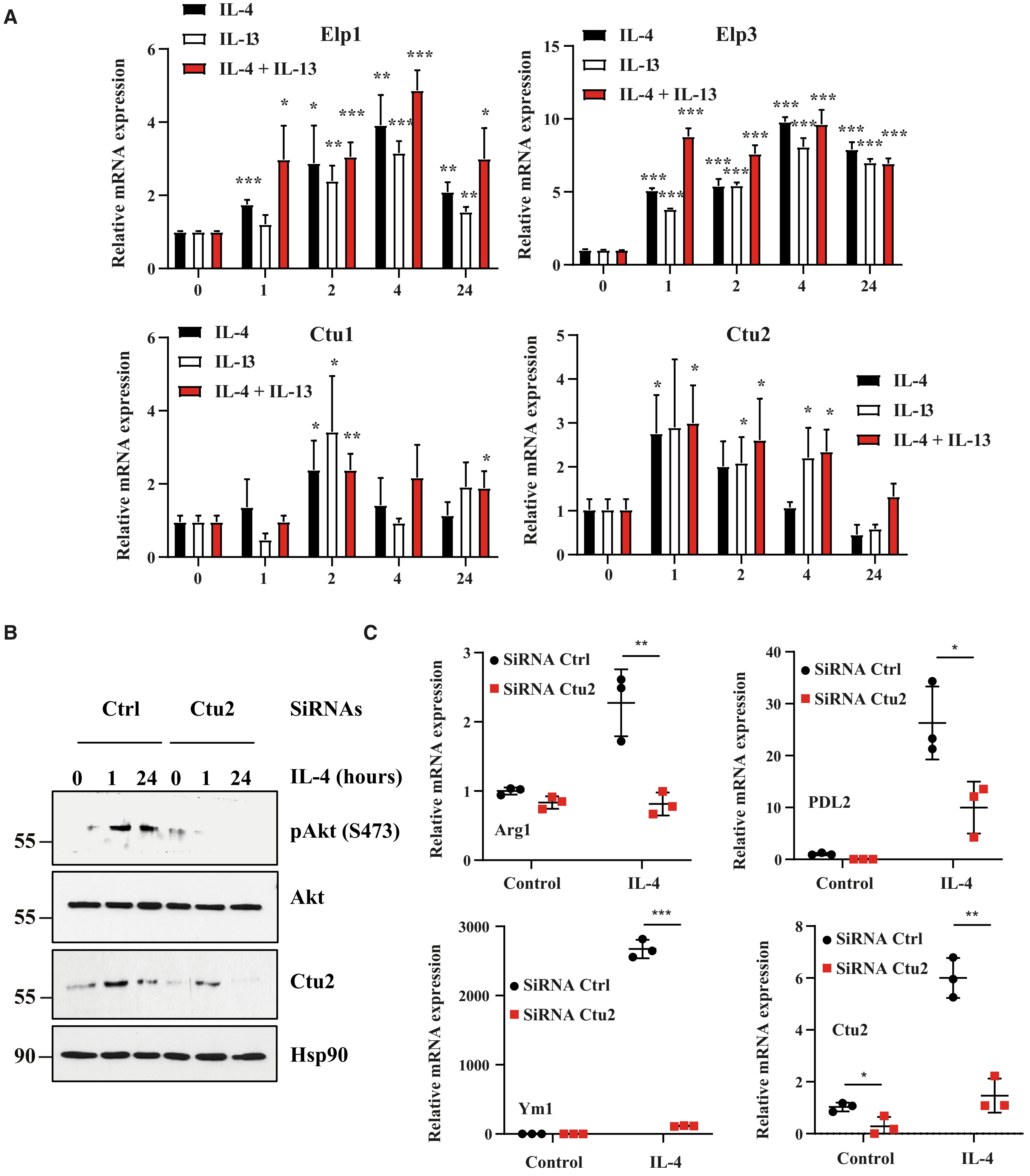

Figure EV2.

 © 2022 The Authors

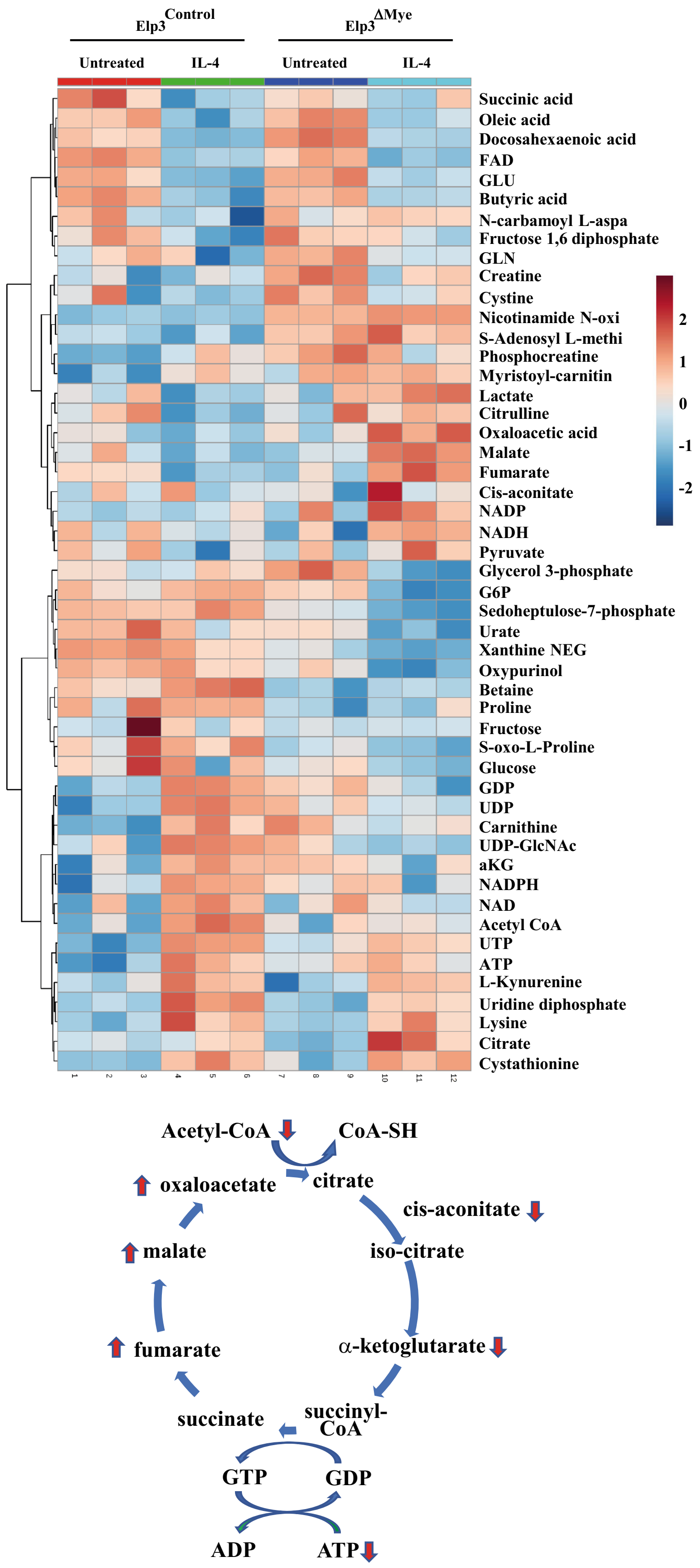

Figure EV3. Metabolic reprogramming by IL-4 relies on Elp3.

Peritoneal macrophages from *Elp3*^{Control} and *Elp3*$^{\Delta Mye}$ mice (n = 3 per genotype) were treated with IL-4 for 24 h *ex-vivo* and the resulting extracts were subjected to Mass Spec analyses to extensively quantify metabolites. At the bottom, defective TCA cycle upon *Elp3* deficiency in IL-4-treated macrophages. Red arrows identify TCA metabolites whose levels change upon *Elp3* deficiency.

© 2022 The Authors

Figure EV4. Elp3 promotes Ric8b expression.

A Elp3 does not regulate Ric8b mRNA levels. mRNAs of BMDMs from the indicated genotypes and stimulated *ex-vivo* with IL-4 or with PBS (control) were subjected to Real-Time PCRs. mRNA levels of Ric8b in BMDMs treated with PBS from $Elp3^{Control}$ mice were set to 1 and levels in other experimental conditions were relative to that after normalization with Gapdh mRNA levels ($n = 3$ mice per genotype (mean ± SD).

B Ric8b promotes LPS-dependent mTORC2 activation. Control or Ric8b-depleted BMDMs were treated or not with IFNγ (50 ng/ml) and LPS (100 ng/ml) for the indicated periods of time and the resulting cell extracts were subjected to WB analyses.

C Increased aggregate formation by IL-4 in macrophages. Flow cytometry of aggresomes with extracts of BMDMs from $Elp3^{Control}$ and $Elp3^{\Delta Mye}$ mice treated with IL-4 (10 ng/ml) for 24 h are illustrated. Experiments were conducted in duplicates.

D Visualization of protein aggregates. Coomassie blue staining of aggregate samples from $Elp3^{Control}$ and $Elp3^{\Delta Mye}$ BMDMs treated with IL-4 or PBS (control; $n = 8$ mice).

E Identification of a proteomic signature of candidates whose expression is increased upon IL-4 stimulation and in BMDMs lacking Elp3 (Venn diagram).

F Identification of proteins enriched in aggregates in macrophages lacking Elp3. Dot plot of protein aggregates from IL-4-treated BMDMs cells lacking or not *Elp3* are shown. Genes enriched in Lys^{AAA}, Gln^{CAA}, and Glu^{GAA} codons are shown in blue. Mrpls are shown in Red.

G The expression of selected Mrpl proteins is controlled by Elp3 in macrophages. Peritoneal macrophages from $Elp3^{Control}$ and $Elp3^{\Delta Mye}$ mice were treated or not with IL-4/IL-13 (10 ng/ml) for the indicated periods of time and cell extracts from resulting cells were subjected to WBs using the indicated antibodies.

H Elp3 does not regulate mRNA levels of Mrpl proteins. PMs from the indicated genotypes were stimulated or not with IL-4/IL-13 (10 ng/ml) for 24 h and mRNA levels of the indicated candidates were quantified by Real-Time PCR. mRNA levels of all candidates in untreated cells from $Elp3^{Control}$ mice were set to 1 and levels in other experimental conditions were relative to that after normalization with Gapdh mRNA levels ($n = 3$ mice, mean ± SD, Student *t*-test, nonsignificant).

I Elp3 controls Mt-co3 expression in macrophages. Protein extracts from PMs of the indicated genotypes treated or not with IL-4/IL-13 (10 ng/ml) for the indicated periods of time were subjected to WB analyses.

Source data are available online for this figure.

 © 2022 The Authors

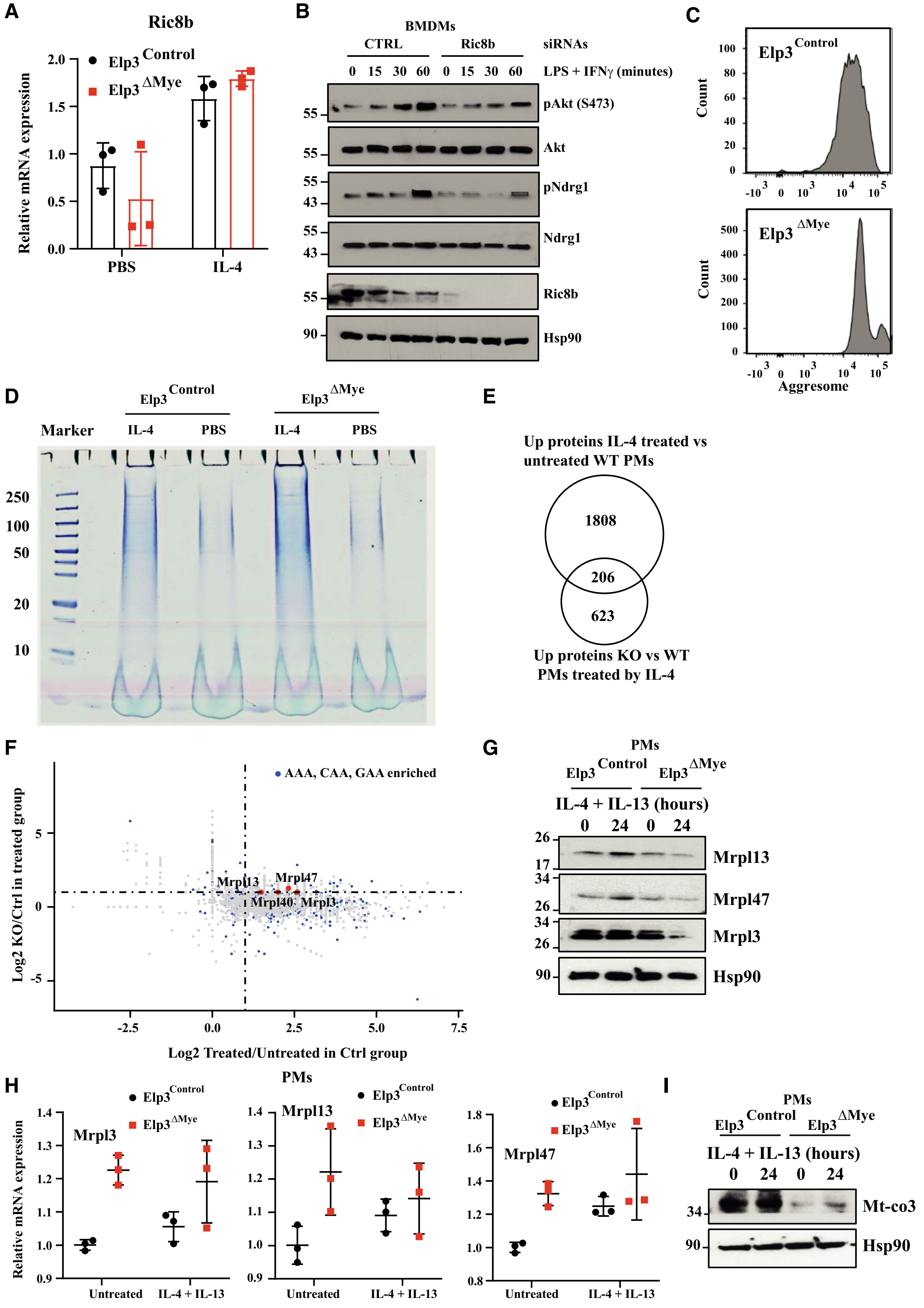
A
Ric8b
Elp3 Control
Elp3 ΔMye
Relative mRNA expression
PBS
IL-4
B
BMDMs
CTRL
Ric8b
siRNAs
0 15 30 60 0 15 30 60
LPS + IFNγ (minutes)
pAkt (S473)
Akt
pNdrg1
Ndrg1
Ric8b
Hsp90
C
Elp3 Control
Elp3 ΔMye
Count
Aggresome
D
Elp3 Control
Elp3 ΔMye
Marker
IL-4
PBS
E
Up proteins IL-4 treated vs untreated WT PMs
1808
206
623
Up proteins KO vs WT PMs treated by IL-4
F
AAA, CAA, GAA enriched
Mrpl13
Mrpl47
Mrpl40
Mrpl3
Log2 KO/Ctrl in treated group
Log2 Treated/Untreated in Ctrl group
G
PMs
Elp3 Control
Elp3 ΔMye
IL-4 + IL-13 (hours)
0 24 0 24
Mrpl13
Mrpl47
Mrpl3
Hsp90
H
PMs
Mrpl3
Mrpl13
Mrpl47
Relative mRNA expression
Untreated
IL-4 + IL-13
I
PMs
Elp3 Control
Elp3 ΔMye
IL-4 + IL-13 (hours)
0 24 0 24
Mt-co3
Hsp90

Figure EV4.

Figure EV5. Elp3 deficiency in peritoneal macrophages does not stabilize Atf4.

A Peritoneal macrophages from the indicated genotypes were stimulated or not with IL-4/IL-13 (10 ng/ml) for the indicated periods of time and the resulting extracts were subjected to WB analyses.

B *Elp3* deficiency does not potentiates Atf4-driven transcription in myeloid cells. Peritoneal macrophages from the indicated genotypes were stimulated or not with IL-4 (10 ng/ml) for 24 h and mRNA levels of the indicated Atf4 target genes were quantified by Real-Time PCR. mRNA levels of all candidates in untreated cells from $Elp3^{Control}$ mice were set to 1 and levels in other experimental conditions were relative to that after normalization with Gapdh mRNA levels (n = 4 mice, mean ± SD, Student t-test, nonsignificant).

Source data are available online for this figure.

© 2022 The Authors

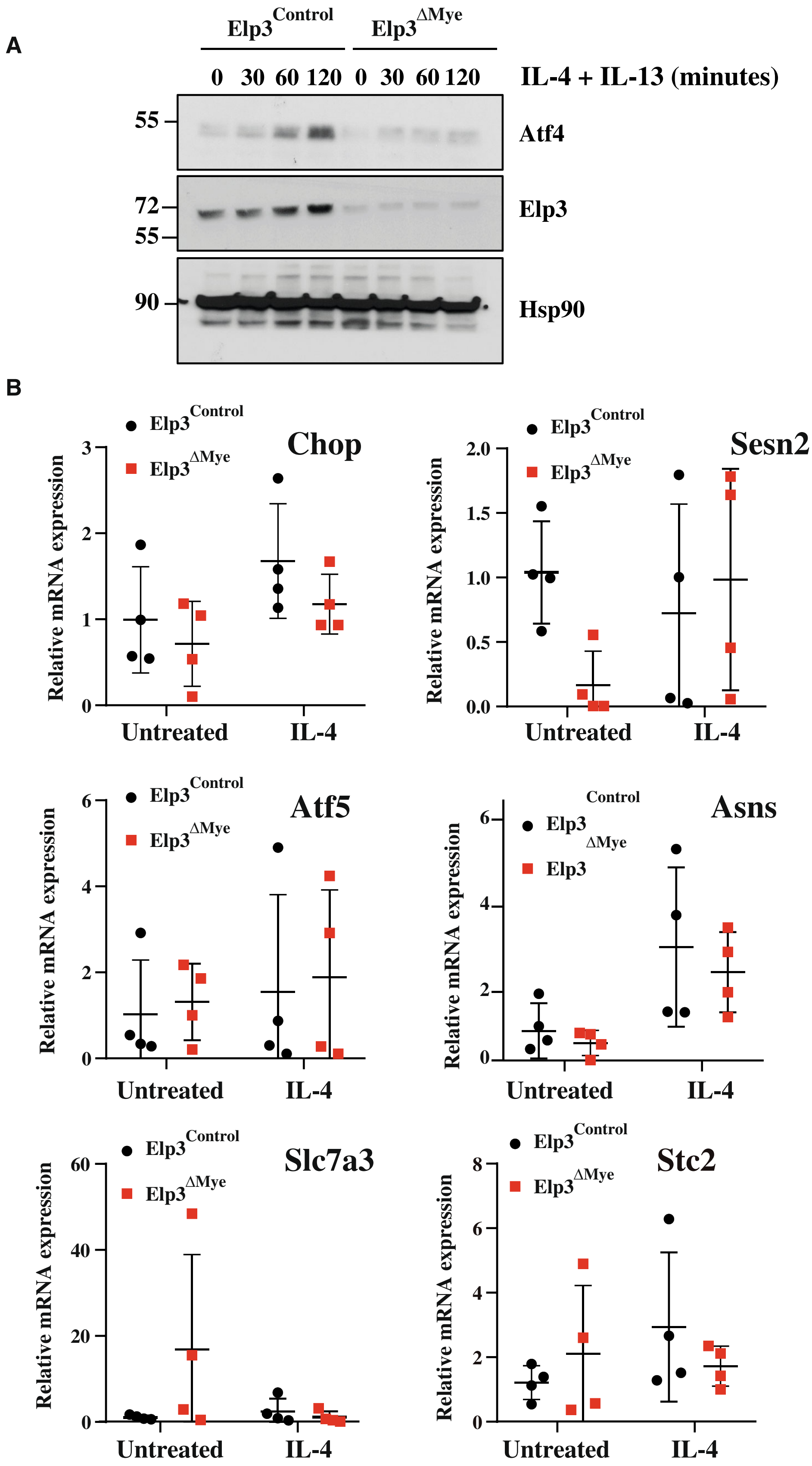
A
Elp3Control
Elp3ΔMye
0 30 60 120 0 30 60 120
IL-4 + IL-13 (minutes)
55
Atf4
72
55
Elp3
90
Hsp90
B
Elp3Control
Elp3ΔMye
Chop
Sesn2
Atf5
Asns
Slc7a3
Stc2
Relative mRNA expression
Untreated
IL-4

Figure EV5.
