# Supplementary material for: Elp3‐mediated codon‐dependent translation promotes mTORC2 activation and regulates macrophage polarization
Source: EMBO J. 2022 Aug 3;41(18):e109353. doi: 10.15252/embj.2021109353 (PMC9475509; doi:10.15252/embj.2021109353)

Figure 3A

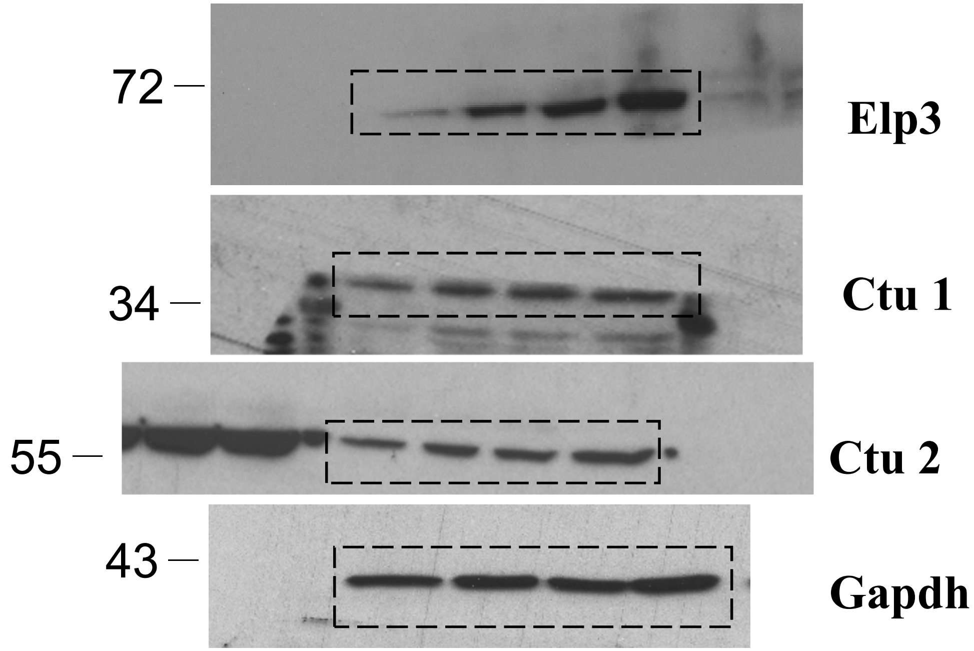

Figure 3B

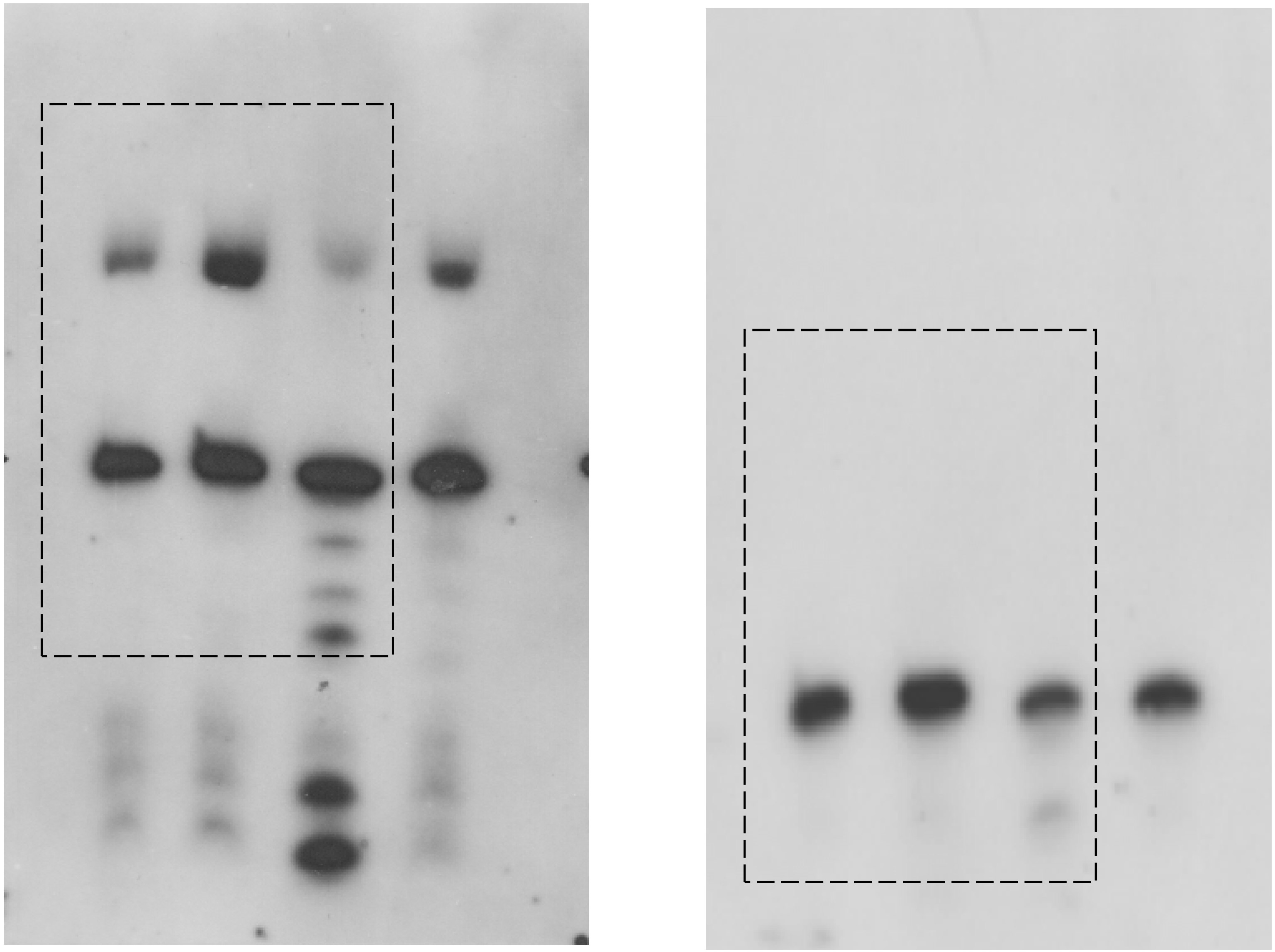

Figure 3C

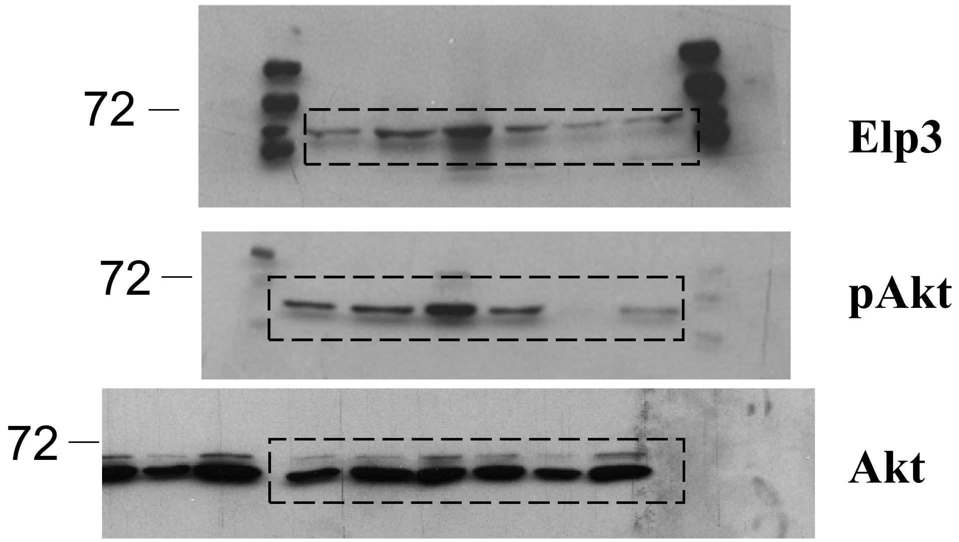

Figure 3E

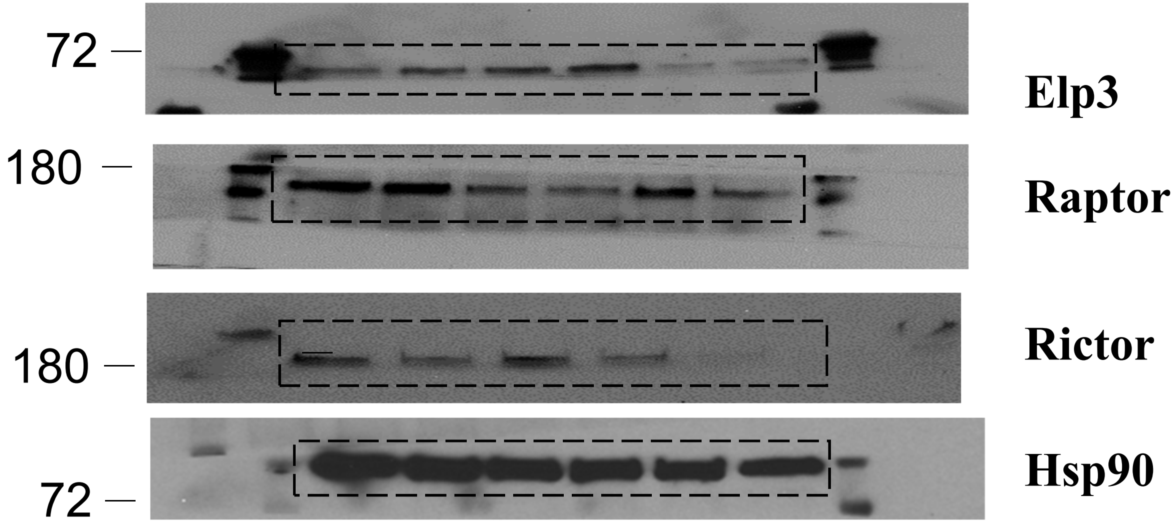

Figure 3F

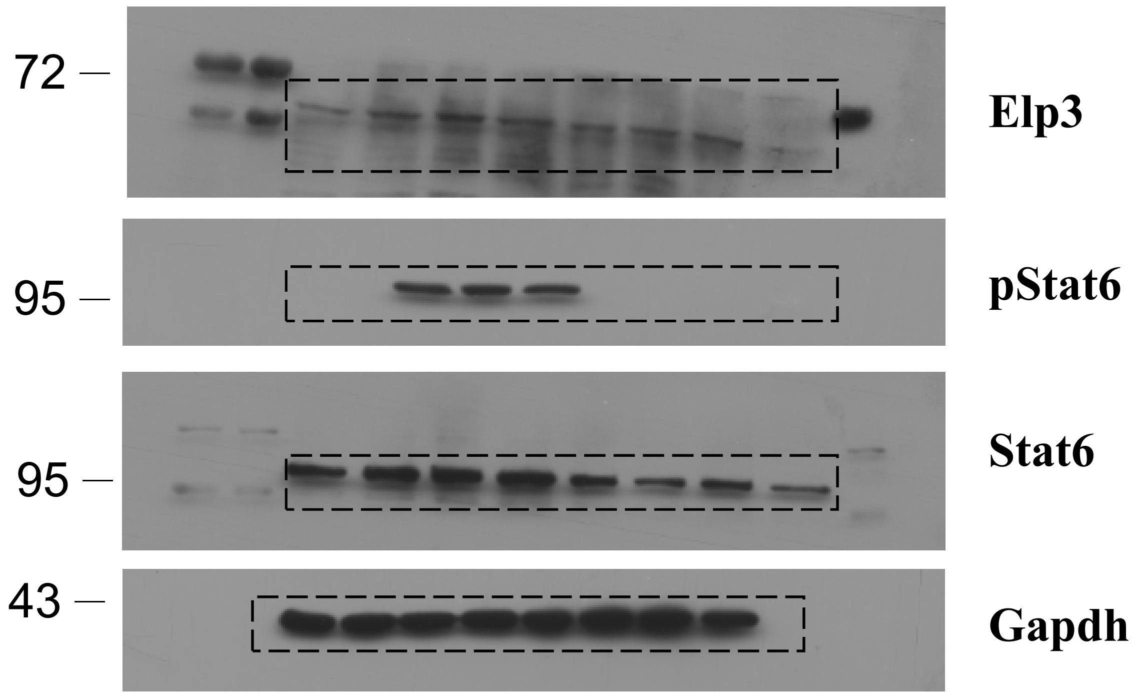

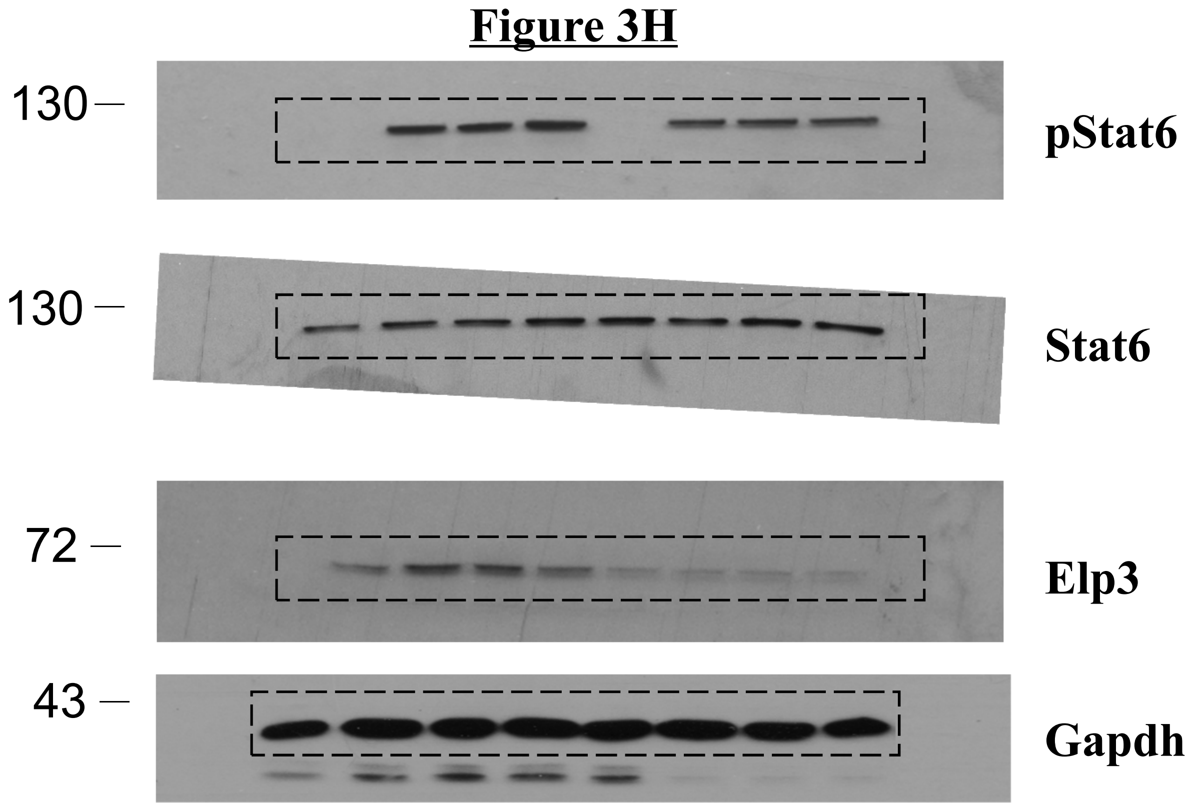
Figure 3H
130
pStat6
130
Stat6
72
Elp3
43
Gapdh

Figure 3I

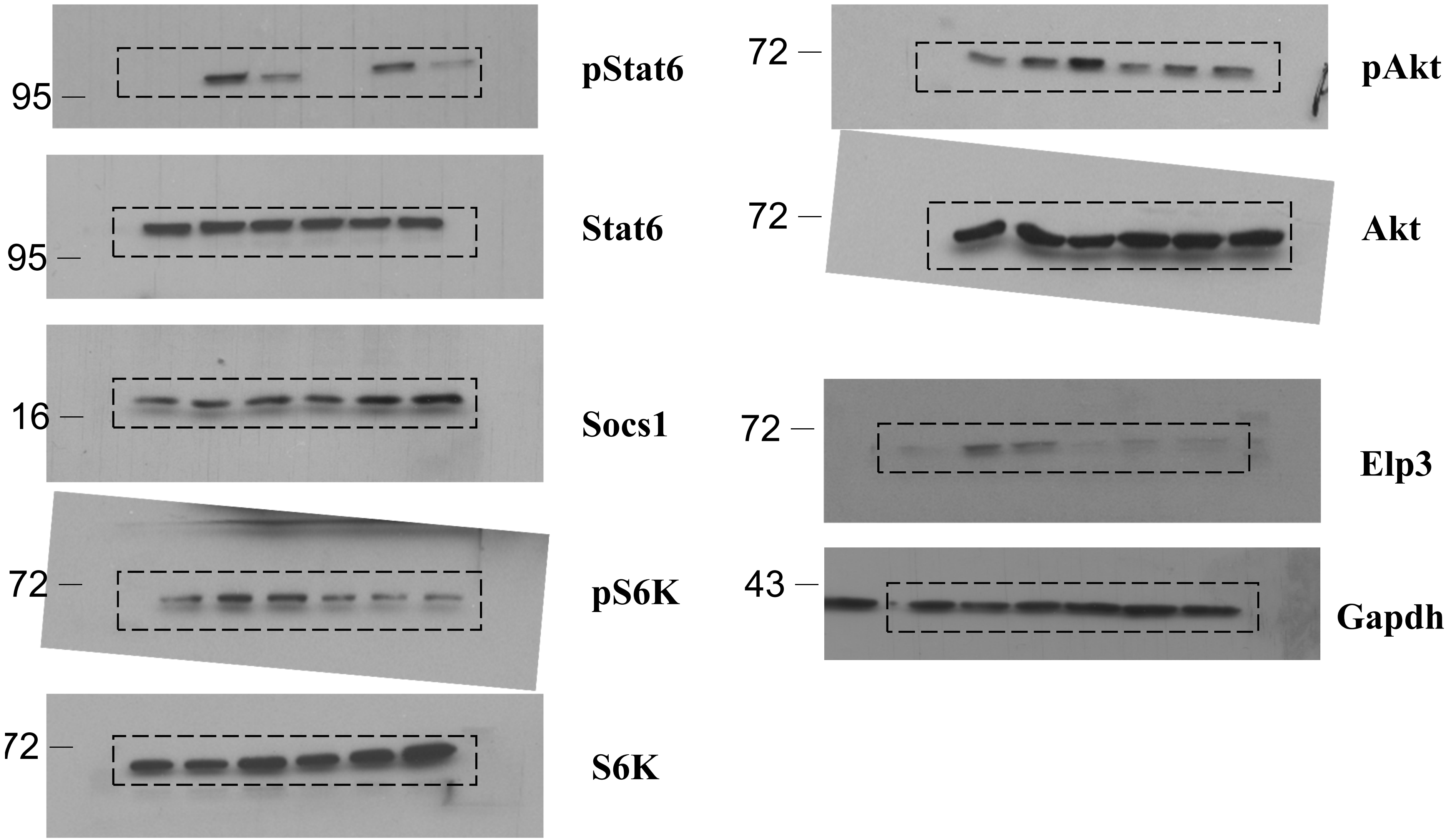

95
pStat6
95
Stat6
16
Socs1
72
pS6K
72
S6K
72
pAkt
72
Akt
72
Elp3
43
Gapdh

Figure 3J

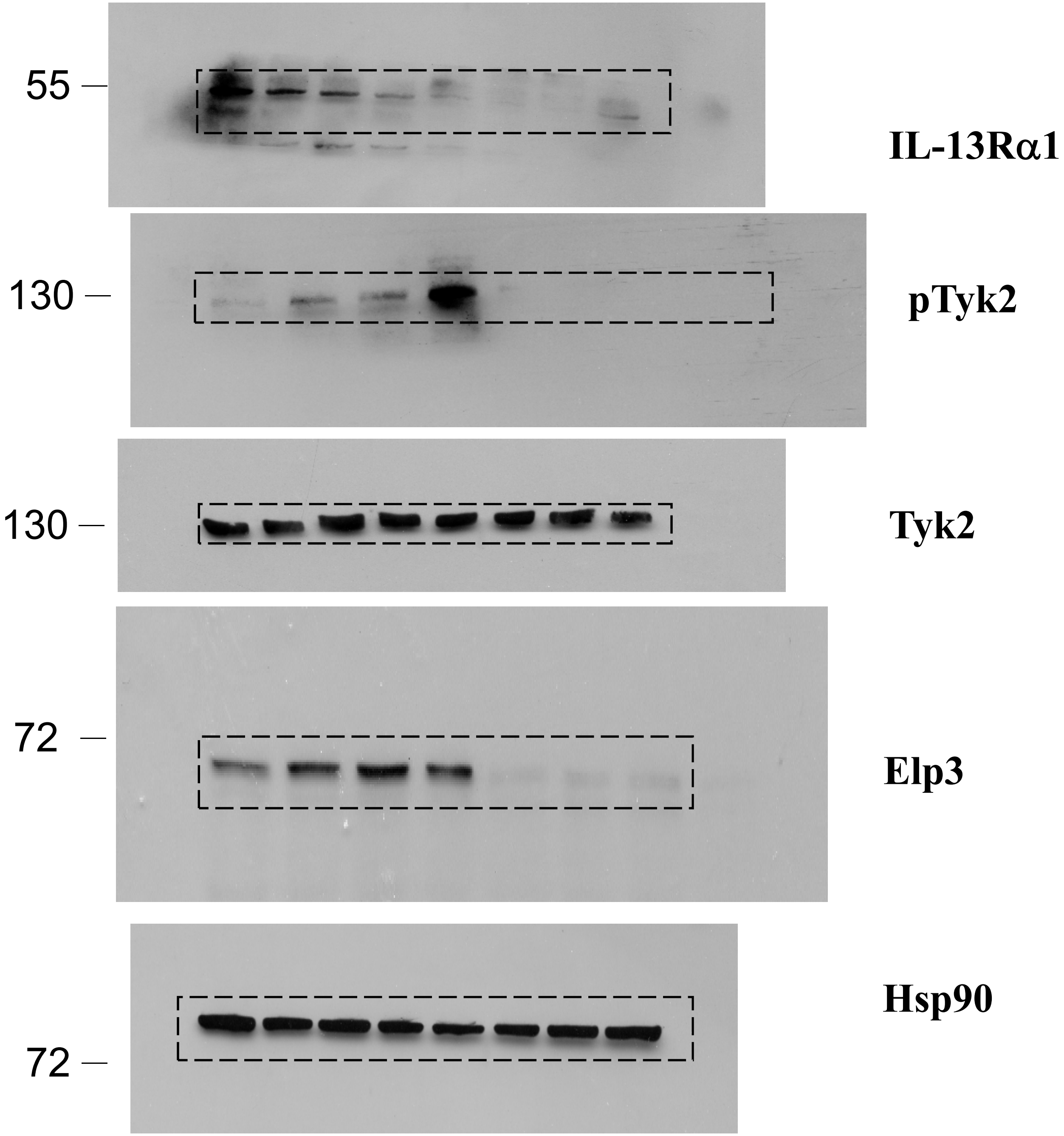

Figure 3L

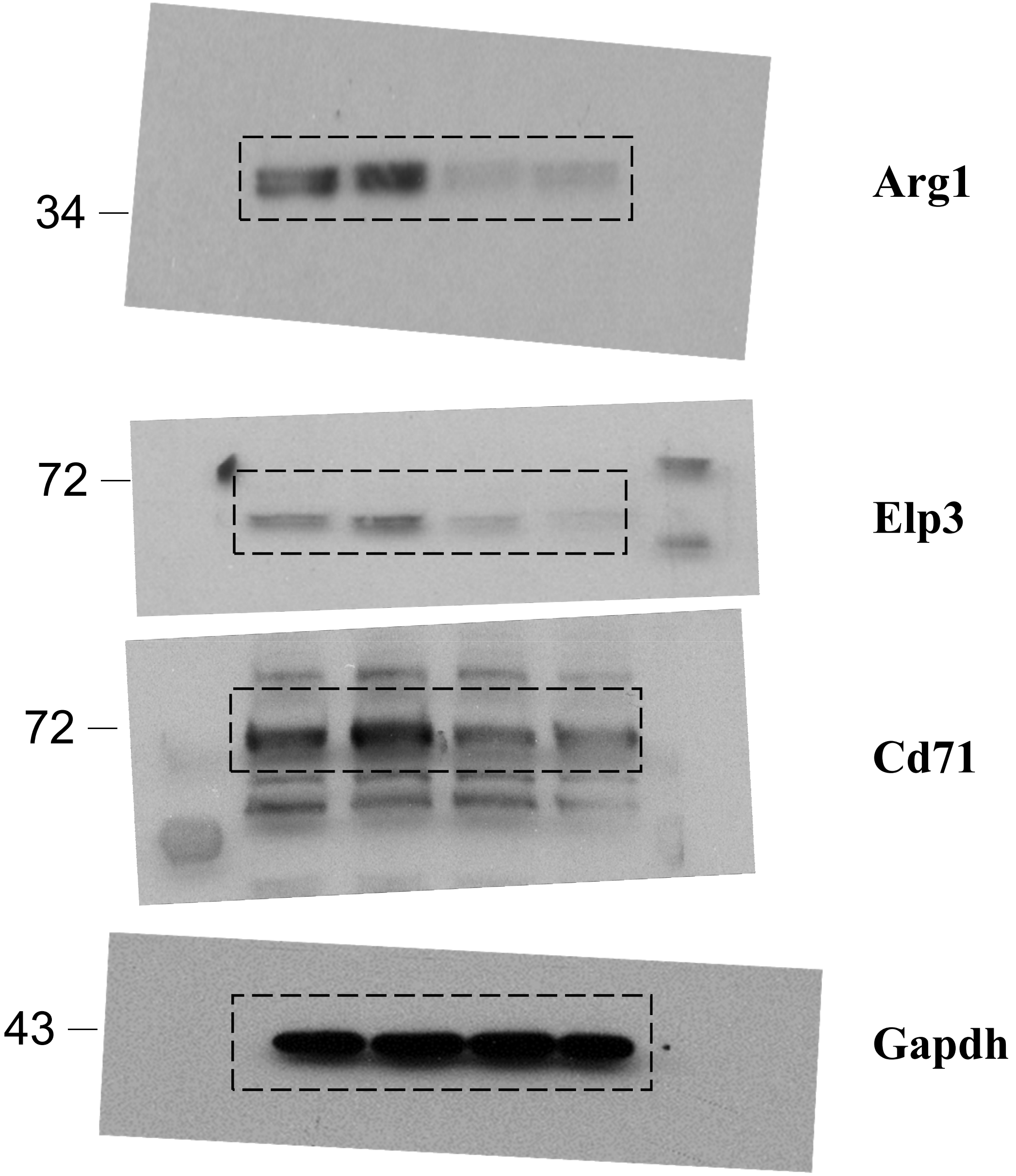

Supplement: Supplementary file 9 — Source Data for Figure 3 [file EMBJ-41-e109353-s007.pdf]
