# Supplementary material for: Elp3‐mediated codon‐dependent translation promotes mTORC2 activation and regulates macrophage polarization
Source: EMBO J. 2022 Aug 3;41(18):e109353. doi: 10.15252/embj.2021109353 (PMC9475509; doi:10.15252/embj.2021109353)
Supplement: Supplementary file 14 [file EMBJ-41-e109353-s014.pdf]

THE EMBO JOURNAL

Elp3-mediated codon-dependent translation promotes mTORC2 activation and regulates macrophage polarization

Dawei Chen, Ivan Nemazanyy, Olivier Peulen, Kateryna Shostak, Xinyi Xu, Seng Chuan Tang, Caroline Wathieu, Silvia Turchetto, Sylvia Tielens, Laurent Nguyen, Pierre Close, Christophe Desmet, Sebastian Klein, Alexandra Florin, Reinhard Buettner, Georgios Petrellis, Benjamin Dewals, and Alain Chariot
DOI: 10.15252/embj.2021109353

Corresponding author(s): Alain Chariot (alain.chariot@uliege.be)

Review Timeline:

Submission Date:	2nd Aug 21
Editorial Decision:	10th Aug 21
Appeal Received:	6th Dec 21
Editorial Decision:	18th Jan 22
Appeal Received:	25th May 22
Editorial Decision:	15th Jun 22
Revision Received:	6th Jul 22
Accepted:	7th Jul 22

Editor: Karin Dumstrei

Transaction Report:

(Note: With the exception of the correction of typographical or spelling errors that could be a source of ambiguity, letters and reports are not edited. Depending on transfer agreements, referee reports obtained elsewhere may or may not be included in this compilation. Referee reports are anonymous unless the Referee chooses to sign their reports.)

1st Editorial Decision **10th Aug 2021**

Dear Prof. Chariot,

Thank you for submitting your manuscript (EMBOJ-2021-109353) to The EMBO Journal. I have now had a chance to read it carefully. In addition, I have also consulted a good expert in the field for advice on the manuscript. I am afraid that we find that the analysis is not well suited for publication in The EMBO Journal and that we therefore have decided not to proceed with full peer-review.

Your analysis reports on the role of the tRNA-modifying enzyme ELP3 on macrophage polarization. The findings show that ELP3 promotes M2 polarization via STAT6 and mTORC2 activation. ELP3 is shown to regulate the expression of the mTORC activator Ric8b. The findings also provide insight into how the expression of ELP3 is regulated. I appreciate that we gain new insight into a function of ELP3 in macrophage polarization. However, we also gain limited further support for if tRNA modifications are altered and how mRNA translation is affected. It is not clear from the present data set if the observed phenotypes are dependent/independent of the tRNA-modifying function of ELP3. We would need further data supporting that the observed phenotypes are indeed cause by altered tRNA modifications in order to consider publication here. This opinion was also shared by the external advisor that I sought advice from.

Should you be able to add such data then I am happy to take another look at the manuscript.

Please note that at the EMBO Journal we subject to external review only those submissions that have a high chance of timely acceptance. I thank you for the opportunity to consider your manuscript for publication here and I am sorry that I can't be more positive on this occasion.

Yours sincerely,

Karin Dumstrei, PhD
Senior Editor
The EMBO Journal

** As a service to authors, EMBO Press provides authors with the possibility to transfer a manuscript that one journal cannot offer to publish to another EMBO publication or the open access journal Life Science Alliance launched in partnership between EMBO Press, Rockefeller University Press and Cold Spring Harbor Laboratory Press. The full manuscript and if applicable, reviewers' reports, are automatically sent to the receiving journal to allow for fast handling and a prompt decision on your manuscript. For more details of this service, and to transfer your manuscript please click on Link Not Available. **

Please do not share this URL as it will give anyone who clicks it access to your account.

Resubmission of Manuscript EMBOJ-2021-109353

Dear Dr. Dumstrei,

We are very happy to resubmit our paper entitled "Elp3 links tRNA modifications to macrophage polarization" to *"The EMBO Journal"*.

Our laboratory has been working for several years on the biological roles played by **U_{34} tRNA modifications** catalyzed by **Elp3**. These chemical tRNA modifications are believed to critically regulate **mRNA translation** but their relevance in health and diseases still remains largely unknown. We previously demonstrated that Elp3-dependent tRNA modifications promote Wnt-driven tumor initiation in the intestine as well as breast cancer metastasis (Ladang et al. *The Journal of Experimental* Medicine, 2015; Delaunay et al. *The Journal of Experimental Medicine*, 2016). We also showed that Elp3 promotes the acquired resistance to B-RAF inhibition in metastatic melanoma, at least by supporting HIF1α synthesis (Rapino et al., *Nature*, 2018). Therefore, U34 tRNA modifications critically regulate tumor development. Our collaborators also demonstrated that U34 tRNA modifications are critical in cortical development as well as in the migration of interneurons during embryogenesis (Laguesse et al. *Developmental Cell*, 2015; Tielens et al. *Cell Research*, 2016). Finally, our collaborators also demonstrated a role of these chemical tRNA modifications in hematopoiesis (Rosu et al. *The Journal of Experimental Medicine*, 2021).

We are now reporting that Elp3-dependent tRNA modifications regulates **macrophage polarization**. This is the very first report in which the roles of U_{34} tRNA modifications in innate immune cells is experimentally addressed. Our work relies on the characterization of several mouse models of diseases in which *Elp3* is genetically inactivated in myeloid cells. We define Ric8b as one candidate whose expression relies on Elp3 and we also provide mechanisms by which Elp3 promotes **mTORC2** activation to support macrophage polarization. We also showed that Elp3 is essential for **mitochondrial functions** in metabolic reprogramming by promoting the expression of some **Mrpl proteins**. Therefore, our work provides key mechanisms by which **mRNA translation regulates the biology of macrophages**.

We previously submitted this manuscript to *EMBO Journal* (Manuscript EMBOJ-2021-109353) and you made some comments on the need to further show that Elp3-dependent tRNA modifications cause the observed phenotype (your e-mail sent to us on August 10th 2021). We experimentally addressed these comments and we now provide a rebuttal letter in which our answers to your comments are described in details (see in the following page).

Rebuttal letter Manuscript EMBOJ-2021-109353

Editorial comment

Your analysis reports on the role of the tRNA-modifying enzyme ELP3 on macrophage polarization. The findings show that ELP3 promotes M2 polarization via STAT6 and mTORC2 activation. ELP3 is shown to regulate the expression of the mTORC activator Ric8b. The findings also provide insight into how the expression of ELP3 is regulated. I appreciate that we gain new insight into a function of ELP3 in macrophage polarization. However, we also gain limited further support for if tRNA modifications are altered and how mRNA translation is affected. It is not clear from the present data set if the observed phenotypes are dependent/independent of the tRNA-modifying function of ELP3. We would need further data supporting that the observed phenotypes are indeed cause by altered tRNA modifications in order to consider publication here.

Our answers

We thank you for the time that you spent in assessing our manuscript. We now provide a revised version in which all your comments were experimentally addressed.

1. *Are tRNA modifications altered ?*

We now show in the newly generated Figure 3B that IL-4/IL-13 increases the pool of thiolated tRNAs while LPS/IFNγ decreases it. This is, to the best of our knowledge, the very first demonstration that some signaling pathways dynamically regulate the pool of chemically-modified tRNAs.

2. *How mRNA translation is affected ?*

We show in several figures that many candidates whose expression is defective in cells lacking Elp3 do NOT show altered mRNA levels (see for example Ric8b in Figure 7A and in Expanded Figure 4A, Mrpl proteins in Figure 8C and in Expanded Figures 6 and 7). Moreover, we also show, using a Puro-PLA assay to detect newly synthesized Ric8b proteins in IL-4-stimulated BMDMs, that Elp3 deficiency interfered with the production of Ric8b proteins (see our newly generated Fig. 7B). Finally, we also demonstrate that Elp3 promotes mRNA translation of Ric8b in a codon-dependent manner (see our newly generated Figure 7C).

3. *Are the observed phenotypes dependent/independent of the tRNA-modifying function of Elp3 ?*

We show that the phenotypes result from the tRNA-modifying function of Elp3 as the deficiency of the thiolases Ctu1/2, which act in the same enzymatic cascade as Elp3, leads to similar consequences on macrophage polarization as Elp3 deficiency (see our Expanded Figures 1 and 2). As Elp3 promotes mTORC2 activation, we cannot rule out the possibility that the stability of some candidates may be regulated by Elp3, as explained in the discussion. It is also possible that some candidates are indirectly regulated at the transcriptional level (see for example some candidates listed in our Heatmap generated with our RNASeq data illustrated in Figure 5D) but our data strongly suggest that Elp3 primarly acts as a tRNA-modifying enzyme.

1st Revision - Editorial Decision **18th Jan 2022**

Dear Alain,

Thank you for submitting your manuscript to The EMBO Journal. Your study has now been seen by three referees and I am afraid that the overall recommendation is not a positive one.

As you can see below, the referees appreciate the reported phenotypes. However, they also find that the analysis adds too limited mechanistic insight and if the phenotypes are linked to altered tRNA modifications. Given this input from good experts in the field, I am afraid that I can't offer to consider publication here.

Should future work allow you to address the raised concerns in full then I can offer to consider a new submission. Please note that for new submissions that we consider the novelty at time of resubmission.

I thank you for the opportunity to consider this manuscript. I am sorry that we cannot be more positive on this occasion.

With best wishes

Karin

Karin Dumstrei, PhD
Senior Editor
The EMBO Journal

****

Referee #1:

In the manuscript by Dawei Chen et al."Elp3 links tRNA modifications to macrophage polarization", the authors showed that the tRNA modifying enzyme Elp3 in myeloid cells promotes FoxO1 phosphorylation by macrophage M1-activating signals, thus limits the production of pro-inflammatory cytokines and attenuates experimental colitis. Induced Elp3 expression promotes both STAT6 and mTORC2 activation and is critical for the IL-4-dependent induction of M2 markers in macrophages. With convincing data, the author demonstrate that Elp3 differentially regulates M1 and M2 macrophage polarization to control intestinal inflammation while promoting tumor development. However, the authors tried to uncover the mechanism of Elp3 regulating macrophage polarization at every aspects, but failed to give an clear mechanistic explanation of the observed cellular and molecular phenotypes.

The logic flow of the manuscript is rather confusing and problematic, re-organizing the data is strongly suggested.

Major Comments:

1. How ELP3 and related complex regulate translation of its target genes, such as showed Ric8b? Fig 7C-F is nice to show that its translation was affected in ELP3 KO, but how?

2. The authors need to show that m5C modification was down in ELP3 KO cells, and better to further show which tRNA's modification were more affected, which may partially explain the target specificity. In other words, why ELP3 KO mainly target genes such as Ric8b, but not other genes? Minimally, similar experiments as shown in fig3B is needed for KO cells.

3. The authors described the dynamic Elp3 expression upon M1 or M2 stimuli, and demonstrated that M2-activating signals upregulate Elp3 expression through a PI3K and STAT6-dependent signaling pathway. What's the molecular mechanism that control Elp3 expression by classical M1-activating signals in macrophages?

4. The Elongator is a complex with multiple subunits (Elp1-Elp6) that promotes transcript elongation and protein translation. Why the authors only choose Elp3 to carry out the following research? What is the expression dynamics of the other subunits upon the M1 and M2 activating signals?

5. The authors need to combine RNA-seq and proteomics to systematically figure out the potential direct targets that were regulated by Elp3 during M1 or M2 stimuli, rather than randomly pick up a gene and demonstrate it.

6. For figure 6,7 and 8, the author explain the mechanism of Elp3 affecting metabolic reprogramming and mitochondrial function. Which one mechanism is most critical? What is the relationship between them? It seems that the author tried to explain the

mechanism of Elp3 regulating M2 macrophage polarization at multiple aspect, but non of them was clearly enough.

7. There are several issues of the Apc+/Min model. 1) Apc+/Min mice not only develop adenomatous polyposis in the small intestine, but also mediate colorectal tumors in large intestine. However, the author seems only count the number of intestinal tumors in the indicated parts of the small intestine, please specify the regions and add the data of colorectal tumors in the figure. 2) TAMs not only have the characteristics of M2 but also share M1 and M2 signature polarization, current studies using Apc+/Min model mainly focus on the M1/M2 balance rather than M2 alone, so maybe it's more suitable for the authors to use the AOM/DSS model or other M2-dominated model to detect the role of Elp3 in modulating M2 function.

Minor points:
1. In Figure 1, the data is not complete and organized well enough. What was the expression changes of Elp3 mRNA in Raw cells upon LPS+IFNγ treatment? How was the protein level of Elp1 in Raw cells upon LPS/IFNγ/LPS+IFNγ treatment? As for lower panel of Figure 1B, the author did not show the Ctu1 and Ctu2 protein level upon LPS treatment. As for Figure 1H, Is there no significant difference of IL6 upon LPS+IFNγ treatment? Why?
2. In Fig 2E and 2F, it is not easy to figure out the differences between Elp3 Control and Elp3 ΔMye mice, please show more representative images.
3. The authors should display the immunofluorescence images at higher magnification meanwhile for Fig 2F and 9D including staining DAPI alone.

4. In Figure 3A, please show the mRNA level of indicated gene upon IL4/IL13/IL4+IL13 treatment. For figure 3B, there is misspell of "IL4 " on the right. For figure 3D, is it shown the Elp3 mRNA level upon IL4 treatment? Or IL4+IL13 treatment? For figure 3G, please supplement mTORC2 CHIP.

5. In Figure 5, the author used BMDMs from Elp3 Control and Elp3 Mye mice treated or not with IL-4 ex-vivo for 2 hours. But the author have not shown the related data upon IL-4 treatment for 2 hours in figure 4. Please explain and supplement experiment at 2 hours.
6. The authors should follow the journal guidelines for using gene and protein names in the manuscript and figures.
7. The sentence "Elp3 expression was detected in RAW 264.7 cells and IFN , but not LPS, severely downregulated Elp3 mRNA and protein levels " should be revised into "Elp3 expression was detected in RAW264.7 cells, and IFN but not LPS, severely downregulated Elp3 mRNA and protein levels ". There are several sentence have this problem in this manuscript.

Referee #2:

In their manuscript "Elp3 links tRNA modifications to macrophage polarization", Chen and co-authors analyze the function of Elongator Elp3 subunit in M1 and M2 macrophage polarization and in the regulation of intestinal inflammation promoting tumor development.

The manuscript is nicely written and the presentation is clear. The authors describe in deep the effect of Elp3 deficiency on macrophage polarization and in response to different stimuli focussing on how the mechanisms are regulated at the level of mRNA translation. Very elegant are the experiments with the Ric8b mutant. Nevertheless, I have some concerns about experimental controls and data interpretation that should be addressed before publication.

Major points:
1) The author should show how Elp3 deficiency affects the levels of U34 tRNA modifications (mcm5, ncm5) in their system, ideally using LC-MS approaches with Elp3ΔMye tRNA. This control is quite important since a lot of data interpretation are based on the assumption that the tRNA modifications are absent or strongly reduced upon Elp3 deficiency. The author should show this also because looking at their Westerns, Elp3 protein in Elp3ΔMye appears to be not completely lost, as can be seen in figures 4d,c, 7a,c, 8c. Maybe this is due to the leakiness of the cre/lox system.

2) Analysis of target tRNA stability upon Elp3 deletion is also missing. This aspect should also be provided to support the interpretation of the data.

3) In figure 1d the authors observe a downregulation of Ctu1, but not Ctu2, both after IFNγ and LPS treatment, but then later they show that Ctu2 deficiency enhances macrophage-driven inflammation in response to LPS and IFNγ (EV figures 1A and 1B). A better discussion of these data should be provided.

4) In figure 2 an untreated control and untreated Elp3ΔMye with DSS should be included as additional control, especially in panels c, d, e, f.

5) A more precise statement should be made for the description of the results of figure 3B. The authors cannot generalize this result to "the pool of thiolated tRNAs", since they used here only a probe against the single tRNAGlnUUG.

6) Figure 7B should be properly label with Elp3Control and Elp3ΔMye. In addition, there is a discrepancy between the time of treatment with 10 μg/ml puromycin (40 minutes in the legend and 5 minutes in the methods). More details need to be added to the methods like the antibodies used in the Duolink PLA assay for a better understanding of the staining procedure and results.

7) For the aggregate isolation it is unclear how the protein loading is normalized in the EV figure 5B. More details should be provided in the legend or in the methods. And how was the classification of U34 sensitive codons in figure 8B was performed? The enrichment of U34 sensitive codons in these selected proteins should be evaluated in the context of all identified proteins in the dataset. Have these proteins the highest content of the U34 translated codons? The authors should clarify this point.

Minor point:
In the abstract "the mitochondrial ribosome large subunits Mrlp3, Mrlp13 and Mrpl47" should be "the mitochondrial ribosome large subunit proteins Mrlp3, Mrlp13 and Mrpl47". As well at the end of the introduction "the production of some mitochondrial ribosome large subunits" should be "the production of some mitochondrial ribosome large subunit proteins". And in the results, "Elp3 promotes the expression of some mitochondrial ribosome large subunit proteins". Also figure 8B should be corrected accordingly.

Referee #3:

In this study, Chen et al demonstrated that tRNA-modifying enzyme Elp3 promotes FoxO1 phosphorylation by M1-activating signals. Elp3 limits the production of pro-inflammatory cytokines to attenuate experimental colitis in mice. In contrast, alternative M2-activating PI3K and STAT6 signals up-regulate Elp3 expression which thereby promotes both STAT6 and mTORC2 activation through the production of Ric8b. Elp3 involves in the transcriptomic and metabolic reprogramming linked to M2 macrophage polarization through the translation of the mitochondrial ribosome large subunits Mplp3, Mrpl13 and Mrpl47. Elp3 also promotes Wnt-driven tumor initiation in the intestine by maintaining a pool of tumor-associated macrophages. This is an interesting work about tRNA modifications in regulating macrophage polarization. However, some experiments are not that solid to draw the conclusion and there are still several deficiencies that need to be addressed.

1. It is surprising that the authors only chose Elp3 to investigate the function in immunology among these Elps. How about the other family members in macrophages?
2. Basically, ELP3 is a tRNA-modifying enzyme, but this manuscript didn't measure any influence on ncm5 or mcm5 or any other RNA modifications. The mechanism seems to be simply related with mTOR consistent with previous literatures, and thereby influence metabolism to affect mitochondrial function. How about the tRNA methylations?
3. Figure 1A-D exhibited only IFNγ but not LPS decreased Elp3 expression in raw cells, but in peritoneal macrophages, it seems LPS also decreased Elp3 in mRNA level but slightly increased in protein level, why?
4. Figure 1H，24h is somehow too long to evaluate the cytokine secretion at mRNA level. Why the expression of these cytokines seem no difference between Elp3 control and ΔMye macrophages when treated with IFNγ but deceased upon LPS combined treatment when compared to LPS treatment only?
5. Figure 4A, the expression of Elp3 decreased upon IL-4 and IL-13 treatment, which was different from previous data (Figure 3A and C). Besides, It seems the expression of Elp3 decreased upon IL-4 with/without IL-13 treatment after 8h to 24h in Figure 3F, Figure 4B and 4D, while accelerated expression was detected in Figure 3A and 3C, why? Moreover, the loading control is not equal.
Minor points:
1. Figure 5D, lack of value bar.
2. Figure 7B, Figure 9B and 9D, lack of scale bar.
3. Figure 7D, a better quality picture may be better to reflect the macrophage accumulation.

** As a service to authors, EMBO Press provides authors with the possibility to transfer a manuscript that one journal cannot offer to publish to another EMBO publication or the open access journal Life Science Alliance launched in partnership between EMBO Press, Rockefeller University Press and Cold Spring Harbor Laboratory Press. The full manuscript and if applicable, reviewers' reports, are automatically sent to the receiving journal to allow for fast handling and a prompt decision on your manuscript. For more details of this service, and to transfer your manuscript please click on Link Not Available. **

Please do not share this URL as it will give anyone who clicks it access to your account.

Resubmission of Manuscript EMBOJ-2021-109353-Q

Dear Dr. Dumstrei,

We are very happy to resubmit our paper now entitled "A codon-dependent translation promotes mTORC2 activation and regulates macrophage polarization" (previously entitled "Elp3 links tRNA modifications to macrophage polarization »). We decided to change the title in order to make it more attractive to the scientific community.

Our laboratory has been working for several years on the biological roles played by **U_{34} tRNA modifications** catalyzed by **Elp3**. These chemical tRNA modifications are believed to critically regulate **mRNA translation** and therefore **translational reprogramming** but their relevance in health and diseases still remains largely unknown. We previously demonstrated that Elp3-dependent tRNA modifications promote Wnt-driven tumor initiation in the intestine as well as breast cancer metastasis (Ladang et al. *The Journal of Experimental* Medicine, 2015; Delaunay et al. *The Journal of Experimental Medicine*, 2016). We also showed that Elp3 promotes the acquired resistance to B-RAF inhibition in metastatic melanoma, at least by supporting HIF1α synthesis (Rapino et al., *Nature*, 2018). Therefore, U34 tRNA modifications critically regulate tumor development. Our collaborators also demonstrated that U34 tRNA modifications are critical in cortical development as well as in the migration of interneurons during embryogenesis (Laguesse et al. *Developmental Cell*, 2015; Tielens et al. *Cell Research*, 2016). Finally, our collaborators also demonstrated a role of these chemical tRNA modifications in hematopoiesis (Rosu et al. *The Journal of Experimental Medicine*, 2021).

We are now reporting that Elp3-dependent tRNA modifications regulates **macrophage polarization**. This is the very first report in which the roles of U_{34} tRNA modifications in innate immune cells is experimentally addressed. Our work relies on the characterization of several mouse models of diseases in which *Elp3* is genetically inactivated in myeloid cells. We define multiple candidates including Ric8b as one candidate whose expression relies on Elp3 and we also provide mechanisms by which Elp3 promotes **mTORC2** activation to support macrophage polarization. We also showed that Elp3 is essential for **mitochondrial functions** in metabolic reprogramming by promoting the expression of some **Mrpl proteins**. Therefore, our work provides key mechanisms by which **mRNA translation regulates the biology of macrophages**.

We previously submitted this manuscript to *EMBO Journal* (Manuscript EMBOJ-2021-109353-Q). We experimentally addressed all comments raised by your reviewers and we now provide a rebuttal letter in which our answers to these comments are described in details.

Referee #1:

In the manuscript by Dawei Chen et al."Elp3 links tRNA modifications to macrophage polarization", the authors showed that the tRNA modifying enzyme Elp3 in myeloid cells promotes FoxO1 phosphorylation by macrophage M1-activating signals, thus limits the production of pro-inflammatory cytokines and attenuates experimental colitis. Induced Elp3 expression promotes both STAT6 and mTORC2 activation and is critical for the IL-4-dependent induction of M2 markers in macrophages. With convincing data, the author demonstrate that Elp3 differentially regulates M1 and M2 macrophage polarization to control intestinal inflammation while promoting tumor development. However, the authors tried to uncover the mechanism of Elp3 regulating macrophage polarization at every aspects, but failed to give an clear mechanistic explanation of the observed cellular and molecular phenotypes.

The logic flow of the manuscript is rather confusing and problematic, re-organizing the data is strongly suggested.

Major Comments:

1. How ELP3 and related complex regulate translation of its target genes, such as showed Ric8b? Fig 7C-F is nice to show that its translation was affected in ELP3 KO, but how?

Our answer:

Studies carried out in yeast (Bauer et al., Cell Reports, 2012, 1(5), 424-433) and more recently in human cells (Rapino et al., Nature, 2018, 558, 605-609; Rapino et al., Nature Communications, 2020, 12(1), 2170) convincingly showed that Elp3 promotes mRNA translation in a codon-dependent manner. This scenario also applies to Ric8b. Indeed, a Ric8b mutant in which Lys^{AAA}, Gln^{CAA}, and Glu^{GAA} codons known to rely on Elp3 to be decoded ("Ric8b WT") where replaced by their cognate synonymous Lys^{AAG}, Gln^{CAG}, and Glu^{GAG} codons which do not require the U34 mcm^5s^2 for their translation ("Ric8b MUT") is not properly expressed in cells lacking Elp3 (Figure 7C). Our revised manuscript now includes new results where we compared our data from both transcriptomic and proteomic analyses (Figures 4E and 4F). These new results indicate that Elp3 regulates the expression of multiple candidates at the protein but not at the transcriptional level. Interestingly, some of these candidates are enriched in Lys^{AAA}, Gln^{CAA}, and Glu^{GAA} codons. Therefore, it is likely that Elp3 regulates macrophage polarization by promoting the mRNA translation of multiple candidates, including Ric8b.

2. The authors need to show that m5C modification was down in ELP3 KO cells, and better to further show which tRNA's modification were more affected, which may partially explain the target specificity. In other words, why ELP3 KO mainly target genes such as Ric8b, but not other genes? Minimally, similar experiments as shown in fig3B is needed for KO cells.

Our answer:

We carried out a new experiment in which all tRNA modifications were quantified in macrophages lacking or not Elp3. These results are illustrated in Figure 1B and show that Elp3 deficiency decreases levels of chemically-modified tRNA harboring the mcm^5s^2 modification, as expected given the key role of Elp3 in this enzymatic cascade. Moreover, we also carried out

additional Northern blots using a variety of probes and extracts from both WT and KO mice, as requested by Reviewer 1. These results are illustrated in the Expanded Figure 1C. Here again, we show that Elp3 deficiency impairs levels of thiolated tRNAs using UUC, UUG and UUU probes. Regarding the specificity of Ric8b, we now show that it is not the only candidate whose mRNA expression is regulated by Elp3 (see Figures 4F and 4G). Yet, we believe that direct Elp3 targets are enriched in defined codons, as explained here before.

3. The authors described the dynamic Elp3 expression upon M1 or M2 stimuli, and demonstrated that M2-activating signals upregulate Elp3 expression through a PI3K and STAT6-dependent signaling pathway. What's the molecular mechanism that control Elp3 expression by classical M1-activating signals in macrophages?

Our answer:

We first assessed mRNA expression of both Elp3 and Elp1 by M1-polarizing signals after 1 hour of stimulation and found that their downregulation was not dramatic (Expanding Figure 1A). Both candidates are more severely downregulated at later time points (up to 6 and 24 hours of stimulation), which indicates that indirect mechanisms are involved. At this point, it is difficult to define any signaling pathway know to be activated by M1 polarizing signals that will ultimately cause Elp3 downregulation. We blocked LPS-dependent ERK1/2 activation using a pharmacological inhibitor and did not find any consequences on LPS-dependent downregulation of Elp3 at late time points (see Figure here after).

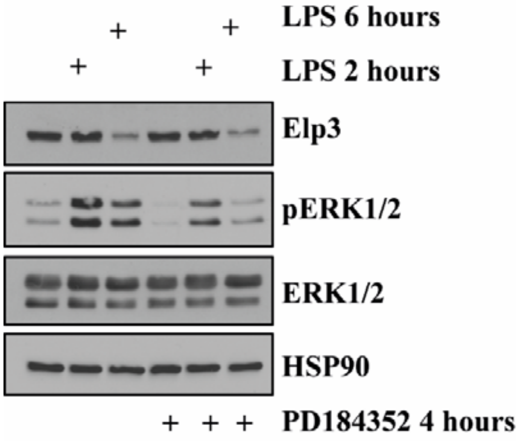

Figure legend : ERK activation is dispensable for LPS-dependent Elp3 downregulation in macrophages. Peritoneal macrophages were pretreated or not with the ERK inhibitor PD184352 (2 µM) for 4 hours and subsequently treated or not with LPS (100 ng/ml) for the indicated periods of time and extracts from the resulting cells were subjected to western blot analyses.

Moreover, Elp3 is not a NF-κB target gene (see the website « NF-κB Target Genes » NF-κB Transcription Factors | Boston University (bu.edu)). To assess whether these M1 polarizing signals cause the destabilization of Elp3 at the protein level, we pre-incubated macrophages with MG132, a proteasome inhibitor and subsequently treated the resulting cells with LPS or with IFNγ. Here again, Elp3 expression was downregulated by both signals but the proteasome was dispensable for these processes as cells pretreated with MG132 and subsequently stimulated with M1 polarizing signals still downregulated Elp3 (Figure here after). Therefore, it is currently unclear how this downregulation occurs.

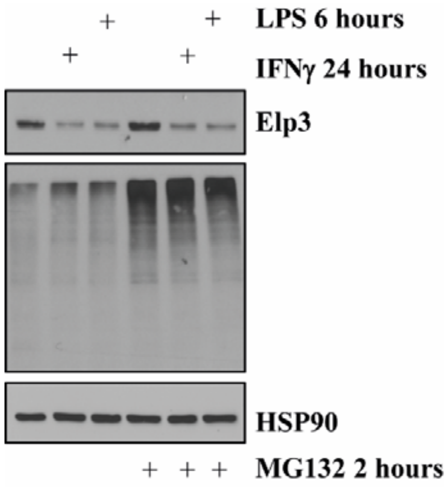

Figure legend. The proteasome is dispensable for the downregulation of Elp3 upon stimulation with M1 polarizing signals. Peritoneal macrophages were unstimulated or pre-incubated with MG132 (10 µM) for 2 hours before being treated or not with LPS (100 ng/ml) or with IFNγ (50 ng/ml) for the indicated periods of time. Extracts from the resulting cells were subjected to western blot analyses. The anti-Ub (Ubiquitin) blot was carried out to prove that the proteasome activity was blocked by MG132, which results in the accumulation of polyubiquitinated candidates.

4. The Elongator is a complex with multiple subunits (Elp1-Elp6) that promotes transcript elongation and protein translation. Why the authors only choose Elp3 to carry out the following research? What is the expression dynamics of the other subunits upon the M1 and M2 activating signals?

Our answer:

We decided to focus on Elp3 as we generated the KO mouse in our laboratory, which is a great experimental tool to assess the biological relevance of these mcm^5s^2 tRNA modifications in vivo. To make the point that our defects are linked to impaired levels of chemically modified tRNAs upon Elp3 deficiency, we also depleted Ctu1/2 (see Figures 1G and Expanded Figures 3B and 3C). So far, we systematically showed that Ctu1/2 deficiency mimicks Elp3 deficiency in multiple experimental models. Regarding other Elongator subunits, let's note that they work in the same enzymatic complex, Elongator, and there is currently no evidence for any subunits having Elongator-independent biological functions. We nevertheless addressed mRNA levels of other Elongator subunits in macrophages treated or not with M1 or M2 polarization signals (see Expanded Figures 1 and 3). We now show that levels of Elp5 remained stable during M1 polarization while both Elp4 and Elp6 levels decreased, mostly at late time points (Expanded Figure 1B). Moreover, IL-4/IL-13 failed to significantly modulate Elp2, Elp4, Elp5 and Elp6 expression (data not shown).

5. The authors need to combine RNA-seq and proteomics to systematically figure out the potential direct targets that were regulated by Elp3 during M1 or M2 stimuli, rather than randomly pick up a gene and demonstrate it.

Our answer:

We carried out the requested analyses (Figures 4E, 4F and 4G) and defined new potential Elp3 targets, in addition to Ric8b. As expected, multiple mRNAs rely on Elp3 to be properly expressed, especially candidates enriched in Lys^{AAA}, Gln^{CAA}, and Glu^{GAA} codons.

6. For figure 6,7 and 8, the author explain the mechanism of Elp3 affecting metabolic reprogramming and mitochondrial function. Which one mechanism is most critical? What is the relationship between them? It seems that the author tried to explain the mechanism of Elp3 regulating M2 macrophage polarization at multiple aspect, but non of them was clearly enough.

Our answer:

At this point, it is not possible to tell which mechanism is the most critical, even if all of them are linked to each others. Our choice is to define and illustrate the complexity of all defects seen upon Elp3 deficiency during macrophage polarization. It is likely that all mechanisms contribute to the observed phenotype, whichs is somehow expected given the fact that Elp3 is critical for the mRNA translation of multiple candidates. We decided to illustrate this complexity rather than focusing on one single mechanism to explain the phenotypical defects. This would have been an oversimplication…Yet, it is clear that Elp3 promotes macrophage polarization, at least through mTORC2 activation, which does not mean that everything goes through this signaling pathway as we also see a defective STAT6 activation in cells lacking Elp3.

Regarding the link between metabolic reprogramming and mitochondrial function, we report that Elp3 deficiency leads to a defective TCA cycle caused by an impaired mitochondrial function (see our functional assays illustrated in Figures 5E, 5F and 5G). Importantly, we show that some mitochondrial large ribosome subunits are not properly expressed in cells lacking Elp3, which could be a mechanism through which Elp3 promotes some mitochondrial function during macrophage polarization, Therefore, we provide a model in which Elp3 promotes the expression of some mitochondrial ribosome large subunits, which is required to support mitochondrial function and the metabolic reprogramming occuring during macrophage polarization.

7. There are several issues of the Apc$^{+/Min}$ model. 1) Apc$^{+/Min}$ mice not only develop adenomatous polyposis in the small intestine, but also mediate colorectal tumors in large intestine. However, the author seems only count the number of intestinal tumors in the indicated parts of the small intestine, please specify the regions and add the data of colorectal tumors in the figure. 2) TAMs not only have the characteristics of M2 but also share M1 and M2 signature polarization, current studies using Apc$^{+/Min}$ model mainly focus on the M1/M2 balance rather than M2 alone, so maybe it's more suitable for the authors to use the AOM/DSS model or other M2-dominated model to detect the role of Elp3 in modulating M2 function.

Our answer:

Apc$^{+/min}$ mice mainly develop adenomas in the small intestine and to a less extent in the distal colon, which is the reason why this experimental model does not perfectly mimick the human disease (please see our previous paper by A. Ladang et al., JEM, 2015, 212, 2057-2075). This phenomemon is also nicely described by Kenneth J. Ritchie and colleagues (PNAS, 2009, 116, 20859-20864). They also report that Apc$^{+/min}$ mice rarely develop colonic tumors in contrast to Apc$^{+/min}$ mice lacking glutathione S-transferase Pi expression. This is the reason why we did not quantify the very few colonic tumors in both genotypes.

We agree with Reviewer 1 that TAMs share some M1 and M2 signatures. As a M2-dominating model, we added more data on mice treated with IL-4c (see our new Figure 6). We show that mice lacking Elp3 expression in myeloid cells failed to produce M2 markers when treated with IL-4c (Figure 6A). Interestingly, we now show that loss of Elp3 impaired IL-4c-induced number of large peritoneal macrophages (LPMs) but did not change the number of small peritoneal macrophages

(Figures 6B and 6C, respectively). To address whether the lack of amplification of LPMs in IL-4c-treated Elp3$^{\Delta Mye}$ mice was due to impaired proliferation, we stained peritoneal cells from both Elp3^{Control} and Elp3$^{\Delta Mye}$ mice for Ki67 and EdU, two markers of cycling cells. Elp3 deficiency decreased the number of both Ki67^{+} LPMs and EdU^{+} PMs (Figures 6D and 6E, respectively). GSEA analyses were conducted and showed prominent enrichment of gene sets related to proliferation, including the mitotic spindle, G2/M checkpoint and E2F targets gene sets, in our RNA-Seq data from IL-4 treated Elp3^{Control} and Elp3$^{\Delta Mye}$ BMDMs (Fig. 6F). Therefore, this is an additional evidence that Elp3 promotes M2 macrophage polarization, at least by supporting macrophage proliferation upon stimulation with IL-4c.

Minor points:

1. **In Figure 1, the data is not complete and organized well enough. What was the expression changes of Elp3 mRNA in Raw cells upon LPS+IFNγ treatment? How was the protein level of Elp1 in Raw cells upon LPS/IFNγ/LPS+IFNγ treatment? As for lower panel of Figure 1B, the author did not show the Ctu1 and Ctu2 protein level upon LPS treatment. As for Figure 1H, Is there no significant difference of IL6 upon LPS+IFNγ treatment? Why?**

Our answer:

Given the multiple panels illustrated in our revised manuscript, we decided to remove our data of expression in RAW 264.7 cells and to exclusively illustrate data with peritoneal macrophages or BMDMs. All western blots and Real-Time PCRs data are illustrated together in the Expanded Figure 1.

Regarding the lack of significant difference of IL-6 upon LPS + IFNγ treatment, we carried out additional experiments with more mice and the resulting data is illustrated in Figure 1E. We now have more IL-6 upon Elp3 deficiency (*, $p<0.05$), which fits with the more severe phenotype seen upon Elp3 deficiency in the experimental colitis model illustrated in Figure 2.

2. **In Fig 2E and 2F, it is not easy to figure out the differences between Elp3^{Control} and Elp3$^{\Delta Mye}$ mice, please show more representative images.**

   Our answer:

   We now provide better images

3. **The authors should display the immunofluorescence images at higher magnification meanwhile for Fig 2F and 9D including staining DAPI alone.**

   Our answer:
   We now provide Figure 2F with the DAPI alone, a scale bar and images at higher magnification for all stainings.

4. **In Figure 3A, please show the mRNA level of indicated gene upon IL4/IL13/IL4+IL13 treatment. For figure 3B, there is misspell of "IL4 " on the right. For figure 3D, is it shown the Elp3 mRNA level upon IL4 treatment? Or IL4+IL13 treatment? For figure 3G, please supplement mTORC2 CHIP.**

Our answer:

Our Real-Time PCR data on Elp1/3 and Ctu1/2 mRNA levels during M2 polarization are illlustrated in Expanded Figure 3. For Figure 3D, it is IL-4 treatment. For Figure 3G, it is unclear why Reviewer 1 would like us to supplement mTORC2 CHIP as mTORC2 is not expected to be recruited on Elp3 promoter.

5. **In Figure 5, the author used BMDMs from $Elp3^{Control}$ and $Elp3^{\Delta Mye}$ mice treated or not with IL-4 ex-vivo for 2 hours. But the author have not shown the related data upon IL-4 treatment for 2 hours in figure 4. Please explain and supplement experiment at 2 hours.**

Our answer:

Data in former Figures 4A, 4B and 4C (now Figures 3H, 3I and 3J, respectively) were concentrating on early events, which is the reason why we showed results obtained with cells treated with M2 polarizing signals from 0, 1 or 24 hours. It is true that we did not show data after 2 hours of stimulation in former Figure 4 as we had no reason to believe that the expression profile would change between 2 and 4 hours of stimulation. We thought that it was more interesting to illustrate data at earlier time points (including after 15 and 30 minutes of stimulation in former Figure 4C, now Figure 3J) in order to make the point that early signaling events are impaired upon IL-4 stimulation in macrophages lacking Elp3.

6. **The authors should follow the journal guidelines for using gene and protein names in the manuscript and figures.**

Our answer:

We wrote names in italic for genes but not for proteins in the revised version.

7. **The sentence "Elp3 expression was detected in RAW 264.7 cells and IFNγ, but not LPS, severely downregulated Elp3 mRNA and protein levels " should be revised into "Elp3 expression was detected in RAW264.7 cells, and IFNγ but not LPS, severely downregulated Elp3 mRNA and protein levels ". There are several sentence have this problem in this manuscript.**

Our answer:

As we removed our data on RAW 264.7 cells, we did not have to make this modification…

Referee #2:

In their manuscript "Elp3 links tRNA modifications to macrophage polarization", Chen and co-authors analyze the function of Elongator Elp3 subunit in M1 and M2 macrophage polarization and in the regulation of intestinal inflammation promoting tumor development.

The manuscript is nicely written and the presentation is clear. The authors describe in deep the effect of Elp3 deficiency on macrophage polarization and in response to different stimuli focussing on how the mechanisms are regulated at the level of mRNA translation. Very elegant are the experiments with the Ric8b mutant. Nevertheless, I have some concerns about experimental controls and data interpretation that should be addressed before publication.

Major points:

1) The author should show how Elp3 deficiency affects the levels of U34 tRNA modifications (mcm5, ncm5) in their system, ideally using LC-MS approaches with Elp3$^{\Delta Mye}$ tRNA. This control is quite important since a lot of data interpretation are based on the assumption that the tRNA modifications are absent or strongly reduced upon Elp3 deficiency. The author should show this also because looking at their Westerns, Elp3 protein in Elp3$^{\Delta Mye}$ appears to be not completely lost, as can be seen in figures 4d,c, 7a,c, 8c. Maybe this is due to the leakiness of the cre/lox system.

Our answer:

We carried out a new experiment in which all tRNA modifications were quantified in macrophages lacking or not Elp3. These results are illustrated in Figure 1B and show that Elp3 deficiency decreases levels of chemically-modified tRNA harboring the mcm^5s^2 modification, as expected given the key role of Elp3 in this enzymatic cascade. Moreover, we also carried out additional Northern blots using a variety of probes and extracts from both WT and KO mice. These results are illustrated in the Expanded Figure 1C. Here again, we show that Elp3 deficiency impairs levels of chemically-modified tRNAs using UUC, UUG and UUU probes.

We agree with Reviewer 2 that Elp3 protein is not totally lost in macrophages from Elp3$^{\Delta Mye}$ mice due to the leakiness of the cre-Lox system. Let's note that this phenomenon was less obvious with the Villin-Cre (see Ladang et al., JEM, 2015, 212, 2057-2075). Another reason may be the fact that ex-vivo cultures of thioglycollate-elicited peritoneal macrophages are not pure as some eosinophils can be found in them, as nicely demonstrated by Alexander V. Misharin and colleagues (Journal of Leukocyte Biology, 2012, 92, 325-331.

2) Analysis of target tRNA stability upon Elp3 deletion is also missing. This aspect should also be provided to support the interpretation of the data.

Our answer:

We did not add this data on tRNA stability as studies carried out by our colleagues in yeast demonstrated that mcm^5s^2 chemical modifications do not influence tRNA stability but only tRNA decoding and translational efficiency.

3) In figure 1d the authors observe a downregulation of Ctu1, but not Ctu2, both after IFNγ and

LPS treatment, but then later they show that Ctu2 deficiency enhances macrophage-driven inflammation in response to LPS and IFNγ (EV figures 1A and 1B). A better discussion of these data should be provided.

Our answer:

We added the following sentence in the discussion : *Although Ctu2 expression is not changed upon stimulation by M1 polarizing signals, Ctu2 deficiency impairs Ctu1 function and consequently the thiolation of some U34 tRNAs. As a result, Ctu2 and Elp3 deficiencies share common consequences on macrophage polarization.* We also mentioned the fact that Ctu1 but not Ctu2 harbors the enzymatic activity in the Introduction. Interfering with Ctu2 levels indirectly impairs Ctu1 function as they both work in the same complex (cf reference by M.Dewez and colleagues that we added in the reference list).

4) In figure 2 an untreated control and untreated Elp3$^{\Delta Mye}$ with DSS should be included as additional control, especially in panels c, d, e, f.

Our answer:

Those requested panels are illustrated in Expanded Figure 2.

5) A more precise statement should be made for the description of the results of figure 3B. The authors cannot generalize this result to "the pool of thiolated tRNAs", since they used here only a probe against the single tRNAGlnUUG.

Our answer:

We now provide additional Northern blots using UUC, UUG and UUU probes and extracts from both WT and KO mice in Expanded Figure 1.

6) Figure 7B should be properly label with Elp3^{Control} and Elp3$^{\Delta Mye}$. In addition, there is a discrepancy between the time of treatment with 10 µg/ml puromycin (40 minutes in the legend and 5 minutes in the methods). More details need to be added to the methods like the antibodies used in the Duolink PLA assay for a better understanding of the staining procedure and results.

Our answer:

We revised the legend and mentioned 5 minutes instead of 40 minutes. We apologize for this mistake. We also add more details in the Methods section for PLA analyses.

7) For the aggregate isolation it is unclear how the protein loading is normalized in the EV figure 5B. More details should be provided in the legend or in the methods. And how was the classification of U34 sensitive codons in figure 8B was performed? The enrichment of U34 sensitive codons in these selected proteins should be evaluated in the context of all identified proteins in the dataset. Have these proteins the highest content of the U34 translated codons? The authors should clarify this point.

Our answer:

For aggregate isolation, the following information was added in the Methods section : *Protein concentration in supernatants was determined with the Bio-Rad Protein Assay. Protein concentration was equalized across samples and aggregates were pelleted from equal amounts of total proteins.*

Regarding the classification of U34 sensitive codons, we added the following information in the methods : *Codon bias analysis, The coding sequence (cds) of all protein-coding Mus musculus genes (genome assembly GRCm38.p6) was downloaded from Ensembl (http://www.ensembl.org). Codon frequency of AAA, CAA, and GAA in the complete cds of every Mouse Genome Informatics–annotated mouse gene was computed using the seqinr and biomaRt packages in R. We next computed a z-score for AAA, CAA, and GAA codon enrichment/ depletion in the cds of proteins found to be downregulated in BMDMs from* $Elp3^{\Delta Mye}$ *mice in our comparative proteomic analysis using the formula: z = (codon frequency of gene – average codon frequency in all mouse cds)/variance of codon frequency in all mouse cds. A heatmap was subsequently generated using the heatmap package in R.*

We also added a new graph in Expanded Figure 6D in which all detected proteins enriched in U34 sensitive codons are plotted (blue dots). Mrpl members listed in Figure 8B were found in aggregates but only some but not all are enriched in U34 sensitive codons.

Minor point:

In the abstract "the mitochondrial ribosome large subunits Mrlp3, Mrlp13 and Mrpl47" should be "the mitochondrial ribosome large subunit proteins Mrlp3, Mrlp13 and Mrpl47". As well at the end of the introduction "the production of some mitochondrial ribosome large subunits" should be "the production of some mitochondrial ribosome large subunit proteins". And in the results, "Elp3 promotes the expression of some mitochondrial ribosome large subunit proteins". Also figure 8B should be corrected accordingly.

<u>Our answer</u>:

These corrections were made.

Referee #3:

In this study, Chen et al demonstrated that tRNA-modifying enzyme Elp3 promotes FoxO1 phosphorylation by M1-activating signals. Elp3 limits the production of pro-inflammatory cytokines to attenuate experimental colitis in mice. In contrast, alternative M2-activating PI3K and STAT6 signals up-regulate Elp3 expression which thereby promotes both STAT6 and mTORC2 activation through the production of Ric8b. Elp3 involves in the transcriptomic and metabolic reprogramming linked to M2 macrophage polarization through the translation of the mitochondrial ribosome large subunits Mrlp3, Mrlp13 and Mrpl47. Elp3 also promotes Wnt-driven tumor initiation in the intestine by maintaining a pool of tumor-associated macrophages. This is an interesting work about tRNA modifications in regulating macrophage polarization. However, some experiments are not that solid to draw the conclusion and there are still several deficiencies that need to be addressed.

1. It is surprising that the authors only chose Elp3 to investigate the function in immunology among these Elps. How about the other family members in macrophages?
2. Basically, ELP3 is a tRNA-modifying enzyme, but this manuscript didn't measure any influence on ncm5 or mcm5 or any other RNA modifications. The mechanism seems to be simply related with mTOR consistent with previous literatures, and thereby influence metabolism to affect mitochondrial function. How about the tRNA methylations?

Our answer:

We decided to focus on Elp3 as we generated the KO mouse in our laboratory, which is a great experimental tool to assess the biological relevance of these mcm^5s^2 tRNA modifications in vivo. To make the point that our defects are linked to impaired levels of chemically modified tRNAs upon Elp3 deficiency, we also depleted Ctu1/2 (see Figures 1G and Expanded Figures 3B and 3C). So far, we systematically showed that Ctu1/2 deficiency mimicks Elp3 deficiency in multiple experimental models. Regarding other Elongator subunits, let's note that they work in the same enzymatic complex, Elongator, and there is currently no evidence for any subunits having Elongator-independent biological functions. We nevertheless addressed mRNA levels of other Elongator subunits in macrophages treated or not with M1 or M2 polarization signals (see Expanded Figures 1 and 3). We also carried out a new experiment in which all tRNA modifications were addressed in cells lacking or not Elp3. We now show that levels of the mcm^5s^2 modification were impaired in cells lacking Elp3, which was expected given the key role of Elp3 in this enzymatic cascade.

Our report describes for the first time that Elp3 promotes mTORC2 activation, at least through the production of Ric8b. This finding has never been reported and explains why mTORC2 and Elp3 deficiencies share common defects in macrophage polarization.

In agreement with data obtained in yeast, Elp3 promotes the mcm^5s^2 modification but not other tRNA methylations (please see our new data illustrated in Figure 1B).

3. Figure 1A-D exhibited only IFNγ but not LPS decreased Elp3 expression in raw cells, but in peritoneal macrophages, it seems LPS also decreased Elp3 in mRNA level but slightly increased in protein level, why?

Our answer:

We agree with Reviewer 3 that data in RAW 264.7 and in peritoneal macrophages are not identical. RAW 264/7 cells are immortalized and this could be a reason for this differential Elp3

expression profile but this is just an hypothesis. As our revised paper now has multiple panels, we decided to remove our expression data of Elongator subunits in RAW 264.7 cells and to exclusively concentrate on data obtained with peritoneal macrophages and with BMDMs. Regarding the decrease of mRNA but not protein levels upon 24 hours of LPS stimulation, we obtained these results multiple times but we do not have any clear mechanism to explain this experimental result. Let's insist on the fact that the downregulation of Elp3 mRNA levels after 24 hours of LPS stimulation, although statistically relevant, is not dramatic. We found that M2 polarization signals more dramatically changed Elp3 expression.

4. Figure 1H, 24h is somehow too long to evaluate the cytokine secretion at mRNA level. Why the expression of these cytokines seem no difference between Elp3^{Control} and $^{\Delta Mye}$ macrophages when treated with IFNγ but decreased upon LPS combined treatment when compared to LPS treatment only?

Our answer:

We agree with Reviewer 3 that 24 hours is a long incubation time in which multiple indirect effects contribute to Elp3 expression but this long incubation time was required to see dramatic changes in Elp3 expression (which is not the case for M2 polarizing signals…). Our new Expanded Figure 1B shows that levels of Elp1/3 barely change after 1 hour of stimulation by LPS and/or IFNγ, which suggest that these M1 polarizing signals do not influence Elp1/3 transcription. This fits with the fact that Elp1/3 are not direct NF-κB target genes, otherwise we would have seen differences after 1 hour of LPS stimulation. Regarding the effects of IFNγ on LPS-dependent signature, we agree with Reviewer 3 that both signals cooperate in macrophage polarization, yet IFNγ can also suppress the induction of some TRL4-activated genes, especially STAT3-dependent genes, as nicely demonstrated by Kyuho Kang and colleagues (Nature Communications 2019, 10, 3320).

5. Figure 4A, the expression of Elp3 decreased upon IL-4 and IL-13 treatment, which was different from previous data (Figure 3A and C). Besides, It seems the expression of Elp3 decreased upon IL-4 with/without IL-13 treatment after 8h to 24h in Figure 3F, Figure 4B and 4D, while accelerated expression was detected in Figure 3A and 3C, why? Moreover, the loading control is not equal.

Our answer:

We thank Reviewer 3 for pointing this issue out and we agree that minor differences in loaded proteins may explain why Elp3 levels appears differentially induced in experiments illustrated in former Figures 3 and 4. It is also fair to say that we are dealing with primary cells and with mice whose capacity to respond to polarizing signals may slightly differ from mice to mice. To summarize this issue, the only discrepancy that we saw is on Elp3 levels in 1 versus 24 hours of stimulation (now Figure 3C versus Figure 4B). For these reasons, we went back to the multiple scans that we generated on these experiments and we now feel comfortable to state that Elp3 levels are more elevated after 24 hours than after 1 hour of stimulation. We added a new scan that better reflects what we saw in multiple experiments. All other parameters in this new Figure 3I remain identical (defective IL-4 dependent Stat6, S6K and Akt phosphorylations upon Elp3 deficiency).

Minor points:

1. Figure 5D, lack of value bar.

Our answer:

The value bar has been added in the revised version.

2. Figure 7B, Figure 9B and 9D, lack of scale bar.
Our answer:

We apologize for this. Scale bars have been added.

3. Figure 7D, a better quality picture may be better to reflect the macrophage accumulation.
Our answer:

We hope that Reviewer 3 will be satisfied with the new picture that we illustrate in the revised version.

2nd Revision - Editorial Decision 15th Jun 2022

Dear Alain,

Thank you for submitting your manuscript to The EMBO Journal. Your study has now been seen by the three referees and their comments are provided below.

As you can see from these comments the referees appreciate the introduced changes and support publication here pending minor changes. Let me know if we need to discuss anything further regarding the last points. Happy to do so via email or video call.

Also, I have asked the editorial assistants and our publisher to do their pre-publication checks on the manuscript and they will get back to me next week with detailed information. I will pass it on to you as soon as I receive it so that you can incorporate it into the revised version.

Congratulations on a nice study - it is almost there!

Best Karin

Karin Dumstrei, PhD
Senior Editor
The EMBO Journal

Use the link below to submit your revision:

https://emboj.msubmit.net/cgi-bin/main.plex

Referee #1:

The authors have addressed most of my previous concerns, with the following minor concerns pending:

1. regarding fig.6/7/8, I understand the complex nature of the ELP3 mechanism, it will be helpful to discuss and try to connect all the explannation in a unified model.
2. regarding fig. 5, I understand that the author want to focus on the early events. It should be point out that the Data in former Figures 5A-5E (now Figures 4A-D, respectively) were made in '2 hours'. Why don't the author treat cell extracts from BMDMs of Elp3Control and Elp3ΔMye mice with IL-4 at much early time point and subject to RNA-Seq experiments, which is more consistent with your data above? The authors need to perform and obtain data more consistently.
3. For Fig.3A, The author demonstrated that IL-4 relies on both Stat6 and mTORC2 to induce Elp3 expression in macrophages. It seems that it was possible for both Stat6 and mTORC2 to recruit on Elp3 promoter in an IL-4-dependent manner, because there is no clear illustration in the main text to analyze the binding site in the Elp3 promoter. Please give more sufficient evidence that mTORC2 is not expected to be recruited on Elp3 promoter.
4. The author may misinterpret the results of Fig 6B-E in the figure legends. For Fig 6B, it only shows "Decreased number of LPMs lacking Elp3 upon stimulation with IL-4c", while the staining of Ki67 and Edu can suggest "The impaired proliferation of large peritoneal macrophages (LPMs) lacking Elp3 during M2 polarization". Please rewrite these figure legends more accurately.
5. Lastly, I haven't seen the genes in italic in the revised version.

Referee #2:

The authors have adequately addressed my major issues and those of the other reviewers, and have improved the manuscript.

Two minor points:

-The distribution of the panels in Fig. 1 should be organized better.

-A quantification of modified/unmodified tRNA in expanded figure 1C is needed to better evaluate the data.

If these are addressed, I support publication in The Embo Journal.

Referee #2:

Minor point：
1. The images in figure 2E are still difficult to distinguish between two groups, although the authors had provided better images. Please show more representative images or higher magnification images.

Referee #1:

The authors have addressed most of my previous concerns, with the following minor concerns pending:

1. Regarding fig.6/7/8, I understand the complex nature of the ELP3 mechanism, it will be helpful to discuss and try to connect all the explanation in a unified model.

Our answer:

We now provide a summary figure in which our major findings are illustrated.

2. Regarding fig. 5, I understand that the author want to focus on the early events. It should be point out that the Data in former Figures 5A-5E (now Figures 4A-D, respectively) were made in '2 hours'. Why don't the author treat cell extracts from BMDMs of Elp3^{Control} and Elp3$^{\Delta Mye}$ mice with IL-4 at much early time point and subject to RNA-Seq experiments, which is more consistent with your data above? The authors need to perform and obtain data more consistently.

Our answer:

The heatmap indeed represents transcriptomic data after 2 hours of stimulation. We selected that time point in order to include both early and late genes induced by IL-4. We may have missed some candidates after one hour only. Note that we did not carry out this analysis after less than one hour of stimulation as we assume that robust gene induction by IL-4 needs at least one hour of stimulation. We also carried out protein analyses at much earlier time points in Figure 3J to assess the activation/phosphorylation of kinases known to be quickly activated by IL-4. Finally, we also assessed protein levels of M2 markers at later time points (24 hours) in Figure 4B, just to make sure that mRNA translation was totally done.

3. For Fig.3A, The author demonstrated that IL-4 relies on both Stat6 and mTORC2 to induce Elp3 expression in macrophages. It seems that it was possible for both Stat6 and mTORC2 to recruit on Elp3 promoter in an IL-4-dependent manner, because there is no clear illustration in the main text to analyze the binding site in the Elp3 promoter. Please give more sufficient evidence that mTORC2 is not expected to be recruited on Elp3 promoter.

Our answer:

We went through the litterature dealing with the biological functions of mTORC2 and did not find any studies reporting the recruitment of mTORC2 to gene promoters. It is nevertheless true that mTORC2, which lacks any DNA-binding site, can be found in the nucleus (see for example the study by Rosner and colleagues, Hum Mol Genet. 2008, 17, 2934-2848). The current model is that mTORC2 may target nuclear transcription factors with potential consequences on their recruitment to gene promoters. Therefore, we reason that the capacity of mTORC2 to regulate Elp3 expression is indirect, i.e. occurs through the phosphorylation of

candidates such as Akt and FoxO1. We now briefly discuss about this issue in the revised version of our manuscript.

4. The author may misinterpret the results of Fig 6B-E in the figure legends. For Fig 6B, it only shows "Decreased number of LPMs lacking Elp3 upon stimulation with IL-4c", while the staining of Ki67 and Edu can suggest "The impaired proliferation of large peritoneal macrophages (LPMs) lacking Elp3 during M2 polarization". Please rewrite these figure legends more accurately.

Our answer:

We thank Reviewer 1 for pointing out this issue. Corrections were made in the legend of Figure 6B in the revised version of our manuscript.

5. Lastly, I haven't seen the genes in italic in the revised version.

Our answer:

Corrections were made in this last version. We thank Reviewer 1 for his/her constructive comments throughout the reviewing process.

Referee #2:

The authors have adequately addressed my major issues and those of the other reviewers, and have improved the manuscript. Two minor points:

-The distribution of the panels in Fig. 1 should be organized better.

Our answer:

We now provide a new version of Figure 1 in which panels were better organized.

-A quantification of modified/unmodified tRNA in expanded figure 1C is needed to better evaluate the data.

Our answer:

These requested quantifications were added in the last version of our manuscript.

If these are addressed, I support publication in The Embo Journal.

Our answer:

We thank Reviewer 2 for his/her constructive comments throughout the reviewing process.

Referee #3:

Minor point：

1. The images in figure 2E are still difficult to distinguish between two groups, although the authors had provided better images. Please show more representative images or higher magnification images.

Our answer:

Better images are provided in our last version. We thank Reviewer 3 for his/her constructive comments throughout the reviewing process.

Quality checks

Please check the distribution of untreated EMP3-dMye in Figure 7B as it looks very similar. Just double check that you entered the proper data.

Our answer:

We checked the original data. The values of y axis are the quotients of two parameters (counts and area). Athough the two similar values of quotients (0.028834 and 0.0334; 0.0648 and 0.072) are similar, the counts and area are quite different.

We can only have 5 keywords - you have now 6

Our answer:

We removed one keyword in our last version.

The conflict of interest statement needs to be updated to Disclosure & Competing Interests Statement - see also our guide to authors

Our answer:

The statement has been updated.

Please make sure that the funding information is entered in to the online submission system as well.

Our answer:

This is done.

The callout to Fig EV6F is missing.

Our answer:

We added this information in the revised version. This is now Fig. EV4H.

The supplementary tables should be labelled as EV Tables 1... Please correct file name and callouts in text.

Our answer:

This is done in the last version.

You have 7 EV figures, but you can only have 5. Can you merge some of them or alternatively you can add them to an appendix. Please note that the appendix needs a ToC. See also guide to authors.

Our answer:

We combined our data into 5 Expanded Figures with corresponding changes both in the text as well as in figure legends.

Heading 'Expanded View Figure Legends' needs adding

- We encourage the publication of source data, particularly for electrophoretic gels and blots, with the aim of making primary data more accessible and transparent to the reader. It would be great if you could provide me with a PDF file per figure that contains the original, uncropped and unprocessed scans of all or key gels used in the figure? The PDF files should be labeled with the appropriate figure/panel number, and should have molecular weight markers; further annotation could be useful but is not essential. The PDF files will be published online with the article as supplementary "Source Data" files.

Our answer:

We now provide this PDF file.

- We include a synopsis of the paper (see http://emboj.embopress.org/). Please provide me with a general summary statement and 3-5 bullet points that capture the key findings of the paper.

Our answer:

Synopsis:

Translational reprogramming occurs during macrophage polarization and involves tRNA modifications. The tRNA-modifying enzyme Elp3 limits M1 but favors M2 macrophage polarization, at least by promoting the translation of Ric8b, a mTORC2 regulator in a codon-dependent manner.

Bullet points

. The tRNA-modifying enzyme Elp3 is downregulated by LPS/IFNγ but induced by IL-4/IL-13

. Elp3 deficiency exacerbates colitis and blocks M2 macrophage polarization in mice.

. Elp3 promotes mTORC2 activation, at least through the mRNA translation of Ric8b in a codon-dependent manner.

. Elp3 promotes metabolic reprogramming linked to M2 polarization, at least through the production of some mitochondrial ribosome large subunit proteins.

- We also need a summary figure for the synopsis. The size should be 550 wide by [200-400] high (pixels). You can also use something from the figures if that is easier.

<u>Our answer:</u>

We are now providing the requested summary figure.

Accepted **7th Jul 2022**

Dear Alain,

Thank you for submitting your revised manuscript to The EMBO Journal. I have now had a chance to look at everything and all looks good.

I am therefore very pleased to accept the MS for publication here.

Congratulations on a nice study

With best wishes

Karin

Karin Dumstrei, PhD
Senior Editor
The EMBO Journal

Please note that it is EMBO Journal policy for the transcript of the editorial process (containing referee reports and your response letter) to be published as an online supplement to each paper. If you do NOT want this, you will need to inform the Editorial Office via email immediately. More information is available here:
https://www.embopress.org/page/journal/14602075/authorguide#transparentprocess

Your manuscript will be processed for publication in the journal by EMBO Press. Manuscripts in the PDF and electronic editions of The EMBO Journal will be copy edited, and you will be provided with page proofs prior to publication. Please note that supplementary information is not included in the proofs.

You will be contacted by Wiley Author Services to complete licensing and payment information. The required 'Page Charges Authorization Form' is available here: https://www.embopress.org/pb-assets/embo-site/tej_apc.pdf - please download and complete the form and return to embopressproduction@wiley.com

Should you be planning a Press Release on your article, please get in contact with embojournal@wiley.com as early as possible, in order to coordinate publication and release dates.

If you have any questions, please do not hesitate to call or email the Editorial Office. Thank you for your contribution to The EMBO Journal.

** Click here to be directed to your login page: https://emboj.msubmit.net

EMBO Press Author Checklist

Corresponding Author Name: Alain CHARIOT
Journal Submitted to: EMBO Journal
Manuscript Number: EMBOJ-2021-109353R1-Q

USEFUL LINKS FOR COMPLETING THIS FORM
The EMBO Journal - Author Guidelines
EMBO Reports - Author Guidelines
Molecular Systems Biology - Author Guidelines
EMBO Molecular Medicine - Author Guidelines

Reporting Checklist for Life Science Articles (updated January 2022)
This checklist is adapted from Materials Design Analysis Reporting (MDAR) Checklist for Authors. MDAR establishes a minimum set of requirements in transparent reporting in the life sciences (see Statement of Task: 10.31222/osf.io/9sm4x). Please follow the journal's guidelines in preparing your manuscript.
Please note that a copy of this checklist will be published alongside your article.

Abridged guidelines for figures
1. Data
The data shown in figures should satisfy the following conditions:
- the data were obtained and processed according to the field's best practice and are presented to reflect the results of the experiments in an accurate and unbiased manner.
- ideally, figure panels should include only measurements that are directly comparable to each other and obtained with the same assay.
- plots include clearly labeled error bars for independent experiments and sample sizes. Unless justified, error bars should not be shown for technical replicates.
- if n<5, the individual data points from each experiment should be plotted. Any statistical test employed should be justified.
- Source Data should be included to report the data underlying figures according to the guidelines set out in the authorship guidelines on Data Presentation.

2. Captions
Each figure caption should contain the following information, for each panel where they are relevant:
- a specification of the experimental system investigated (eg cell line, species name).
- the assay(s) and method(s) used to carry out the reported observations and measurements.
- an explicit mention of the biological and chemical entity(ies) that are being measured.
- an explicit mention of the biological and chemical entity(ies) that are altered/varied/perturbed in a controlled manner.
- the exact sample size (n) for each experimental group/condition, given as a number, not a range;
- a description of the sample collection allowing the reader to understand whether the samples represent technical or biological replicates (including how many animals, litters, cultures, etc.).
- a statement of how many times the experiment shown was independently replicated in the laboratory.
- definitions of statistical methods and measures:
  - common tests, such as t-test (please specify whether paired vs. unpaired), simple χ2 tests, Wilcoxon and Mann-Whitney tests, can be unambiguously identified by name only, but more complex techniques should be described in the methods section;
  - are tests one-sided or two-sided?
  - are there adjustments for multiple comparisons?
  - exact statistical test results, e.g., P values = x but not P values < x;
  - definition of 'center values' as median or average;
  - definition of error bars as s.d. or s.e.m.

Please complete ALL of the questions below.
Select "Not Applicable" only when the requested information is not relevant for your study.

Materials

Newly Created Materials	Information included in the manuscript?	In which section is the information available? (Reagents and Tools Table, Materials and Methods, Figures, Data Availability Section)
New materials and reagents need to be available; do any restrictions apply?	Not Applicable	

Antibodies	Information included in the manuscript?	In which section is the information available? (Reagents and Tools Table, Materials and Methods, Figures, Data Availability Section)
For **antibodies** provide the following information:<br>- Commercial antibodies: RRID (if possible) or supplier name, catalogue number and or/clone number<br>- Non-commercial: RRID or citation	Yes	Supplementary Tables 1 to 3

DNA and RNA sequences	Information included in the manuscript?	In which section is the information available? (Reagents and Tools Table, Materials and Methods, Figures, Data Availability Section)
**Short novel DNA or RNA including primers, probes**: provide the sequences.	Not Applicable	

Cell materials	Information included in the manuscript?	In which section is the information available? (Reagents and Tools Table, Materials and Methods, Figures, Data Availability Section)
**Cell lines:** Provide species information, strain. Provide accession number in repository **OR** supplier name, catalog number, clone number, and/**OR** RRID.	Yes	Methods section
**Primary cultures:** Provide species, strain, sex of origin, genetic modification status.	Yes	Methods section
Report if the cell lines were recently **authenticated** (e.g., by STR profiling) and tested for mycoplasma contamination.	Yes	Methods section

Experimental animals	Information included in the manuscript?	In which section is the information available? (Reagents and Tools Table, Materials and Methods, Figures, Data Availability Section)
**Laboratory animals or Model organisms:** Provide species, strain, sex, age, genetic modification status. Provide accession number in repository **OR** supplier name, catalog number, clone number, **OR** RRID.	Yes	Methods section
**Animal observed in or captured from the field:** Provide species, sex, and age where possible.	Not Applicable	Methods section
Please detail **housing and husbandry conditions**.	Yes	Methods section

Plants and microbes	Information included in the manuscript?	In which section is the information available? (Reagents and Tools Table, Materials and Methods, Figures, Data Availability Section)
**Plants:** provide species and strain, ecotype and cultivar where relevant, unique accession number if available, and source (including location for collected wild specimens).	Not Applicable	
**Microbes:** provide species and strain, unique accession number if available, and source.	Not Applicable	

Human research participants	Information included in the manuscript?	In which section is the information available? (Reagents and Tools Table, Materials and Methods, Figures, Data Availability Section)
If collected and within the bounds of privacy constraints report on age, sex and gender or ethnicity for all study participants.	Not Applicable	

Core facilities	Information included in the manuscript?	In which section is the information available? (Reagents and Tools Table, Materials and Methods, Figures, Data Availability Section)
If your work benefited from core facilities, was their service mentioned in the acknowledgments section?	Yes	Acknowledgements

Design

Study protocol	Information included in the manuscript?	In which section is the information available? (Reagents and Tools Table, Materials and Methods, Figures, Data Availability Section)
If study protocol has been **pre-registered, provide DOI in the manuscript**. For clinical trials, provide the trial registration number **OR** cite DOI.	Not Applicable	
Report the **clinical trial registration number** (at ClinicalTrials.gov or equivalent), where applicable.	Not Applicable	

Laboratory protocol	Information included in the manuscript?	In which section is the information available? (Reagents and Tools Table, Materials and Methods, Figures, Data Availability Section)
Provide DOI OR other citation details if **external detailed step-by-step protocols** are available.	Not Applicable	

Experimental study design and statistics	Information included in the manuscript?	In which section is the information available? (Reagents and Tools Table, Materials and Methods, Figures, Data Availability Section)

Include a statement about **sample size** estimate even if no statistical methods were used.	Not Applicable	
Were any steps taken to minimize the effects of subjective bias when allocating animals/samples to treatment (e.g. **randomization procedure**)? If yes, have they been described?	Yes	Methods section
Include a statement about **blinding** even if no blinding was done.	Yes	Methods section
Describe **inclusion/exclusion criteria** if samples or animals were excluded from the analysis. Were the criteria pre-established?<br><br>If sample or data points were omitted from analysis, report if this was due to attrition or intentional exclusion and provide justification.	Not Applicable	
For every figure, are **statistical tests** justified as appropriate? Do the data meet the assumptions of the tests (e.g., normal distribution)? Describe any methods used to assess it. Is there an estimate of variation within each group of data? Is the variance similar between the groups that are being statistically compared?	Yes	Methods section

Sample definition and in-laboratory replication	Information included in the manuscript?	In which section is the information available?<br>(Reagents and Tools Table, Materials and Methods, Figures, Data Availability Section)
In the figure legends: state number of times the experiment was **replicated** in laboratory.	Yes	Figure legends
In the figure legends: define whether data describe **technical or biological replicates**.	Yes	Figure legends

Ethics

Ethics	Information included in the manuscript?	In which section is the information available?<br>(Reagents and Tools Table, Materials and Methods, Figures, Data Availability Section)
Studies involving **human participants**: State details of **authority granting ethics approval** (IRB or equivalent committee(s), provide reference number for approval.	Not Applicable	
Studies involving **human participants**: Include a statement confirming that **informed consent** was obtained from all subjects and that the experiments conformed to the principles set out in the WMA Declaration of Helsinki and the Department of Health and Human Services Belmont Report.	Not Applicable	
Studies involving **human participants:** For publication of **patient photos**, include a statement confirming that consent to publish was obtained.	Not Applicable	
Studies involving experimental **animals**: State details of **authority granting ethics approval** (IRB or equivalent committee(s), provide reference number for approval. Include a statement of compliance with ethical regulations.	Yes	Methods section
Studies involving **specimen and field samples:** State if relevant **permits** obtained, provide details of authority approving study; if none were required, explain why.	Not Applicable	

Dual Use Research of Concern (DURC)	Information included in the manuscript?	In which section is the information available?<br>(Reagents and Tools Table, Materials and Methods, Figures, Data Availability Section)
Could your study fall under dual use research restrictions? Please check biosecurity documents and list of **select agents and toxins** (CDC): https://www.selectagents.gov/sat/list.htm	Not Applicable	
If you used a select agent, is the security level of the lab appropriate and reported in the manuscript?	Not Applicable	
If a study is subject to dual use research of concern regulations, is the name of the **authority granting approval and reference number** for the regulatory approval provided in the manuscript?	Not Applicable	

Reporting

The MDAR framework recommends adoption of discipline-specific guidelines, established and endorsed through community initiatives. Journals have their own policy about requiring specific guidelines and recommendations to complement MDAR.

Adherence to community standards	Information included in the manuscript?	In which section is the information available?<br>(Reagents and Tools Table, Materials and Methods, Figures, Data Availability Section)
State if relevant guidelines or checklists (e.g., **ICMJE, MIBBI, ARRIVE, PRISMA**) have been followed or provided.	Not Applicable	
For **tumor marker prognostic studies,** we recommend that you follow the **REMARK** reporting guidelines (see link list at top right). See author guidelines, under 'Reporting Guidelines'. Please confirm you have followed these guidelines.	Not Applicable	
For **phase II and III randomized controlled trials**, please refer to the **CONSORT** flow diagram (see link list at top right) and submit the CONSORT checklist (see link list at top right) with your submission. See author guidelines, under 'Reporting Guidelines'. Please confirm you have submitted this list.	Not Applicable	

Data Availability

Data availability	Information included in the manuscript?	In which section is the information available?<br>(Reagents and Tools Table, Materials and Methods, Figures, Data Availability Section)
Have **primary datasets** been deposited according to the journal's guidelines (see 'Data Deposition' section) and the respective accession numbers provided in the Data Availability Section?	Yes	Methods section
Were **human clinical and genomic datasets** deposited in a public access-controlled repository in accordance to ethical obligations to the patients and to the applicable consent agreement?	Not Applicable	
Are **computational models** that are central and integral to a study available without restrictions in a machine-readable form? Were the relevant accession numbers or links provided?	Not Applicable	
If publicly available data were reused, provide the respective **data citations in the reference list**.	Not Applicable	
